# Faecal microbiota transplant ameliorates gut dysbiosis and cognitive deficits in Huntington’s disease mice

**DOI:** 10.1093/braincomms/fcac205

**Published:** 2022-08-12

**Authors:** Carolina Gubert, Jocelyn M Choo, Chloe J Love, Saritha Kodikara, Bethany A Masson, Jamie J M Liew, Yiwen Wang, Geraldine Kong, Vinod K Narayana, Thibault Renoir, Kim Anh Lê Cao, Geraint B Rogers, Anthony J Hannan

**Affiliations:** Florey Institute of Neuroscience and Mental Health, Melbourne Brain Centre, University of Melbourne, Parkville, VIC 3010, Australia; Microbiome and Host Health, South Australian Health and Medical Research Institute, Adelaide, SA 5001, Australia; Infection and Immunity, Flinders Health and Medical Research Institute, College of Medicine and Public Health, Flinders University, Bedford Park, SA 5042, Australia; Florey Institute of Neuroscience and Mental Health, Melbourne Brain Centre, University of Melbourne, Parkville, VIC 3010, Australia; Melbourne Integrative Genomics, School of Mathematics and Statistics, University of Melbourne, Parkville, VIC 3010, Australia; Florey Institute of Neuroscience and Mental Health, Melbourne Brain Centre, University of Melbourne, Parkville, VIC 3010, Australia; Florey Institute of Neuroscience and Mental Health, Melbourne Brain Centre, University of Melbourne, Parkville, VIC 3010, Australia; Melbourne Integrative Genomics, School of Mathematics and Statistics, University of Melbourne, Parkville, VIC 3010, Australia; Florey Institute of Neuroscience and Mental Health, Melbourne Brain Centre, University of Melbourne, Parkville, VIC 3010, Australia; Bio21 Institute and Department of Biochemistry and Molecular Biology, University of Melbourne, Parkville, VIC 3010, Australia; Florey Institute of Neuroscience and Mental Health, Melbourne Brain Centre, University of Melbourne, Parkville, VIC 3010, Australia; Melbourne Integrative Genomics, School of Mathematics and Statistics, University of Melbourne, Parkville, VIC 3010, Australia; Microbiome and Host Health, South Australian Health and Medical Research Institute, Adelaide, SA 5001, Australia; Infection and Immunity, Flinders Health and Medical Research Institute, College of Medicine and Public Health, Flinders University, Bedford Park, SA 5042, Australia; Florey Institute of Neuroscience and Mental Health, Melbourne Brain Centre, University of Melbourne, Parkville, VIC 3010, Australia; Department of Anatomy and Neuroscience, University of Melbourne, Parkville, VIC 3010, Australia

**Keywords:** dementia, gut microbiome, Huntington’s disease, microbiota–gut–brain axis, neurodegenerative disorder

## Abstract

Huntington’s disease is a neurodegenerative disorder involving psychiatric, cognitive and motor symptoms. Huntington’s disease is caused by a tandem-repeat expansion in the huntingtin gene, which is widely expressed throughout the brain and body, including the gastrointestinal system. There are currently no effective disease-modifying treatments available for this fatal disorder. Despite recent evidence of gut microbiome disruption in preclinical and clinical Huntington’s disease, its potential as a target for therapeutic interventions has not been explored. The microbiota–gut–brain axis provides a potential pathway through which changes in the gut could modulate brain function, including cognition. We now show that faecal microbiota transplant (FMT) from wild-type into Huntington’s disease mice positively modulates cognitive outcomes, particularly in females. In Huntington’s disease male mice, we revealed an inefficiency of FMT engraftment, which is potentially due to the more pronounced changes in the structure, composition and instability of the gut microbial community, and the imbalance in acetate and gut immune profiles found in these mice. This study demonstrates a role for gut microbiome modulation in ameliorating cognitive deficits modelling dementia in Huntington’s disease. Our findings pave the way for the development of future therapeutic approaches, including FMT and other forms of gut microbiome modulation, as potential clinical interventions for Huntington’s disease.

## Teaser

Gubert *et al.* report that faecal microbiota transplant from wild-type into Huntington’s disease mice positively modulates cognitive outcomes, particularly in females. They demonstrate sexually dimorphic and progressive gut dysfunction, and microbial disruption, and provide the first evidence supporting microbiota manipulation as a therapeutic intervention for Huntington’s disease, particularly cognitive symptoms.

## Graphical Abstract

**Graphical abstract:**
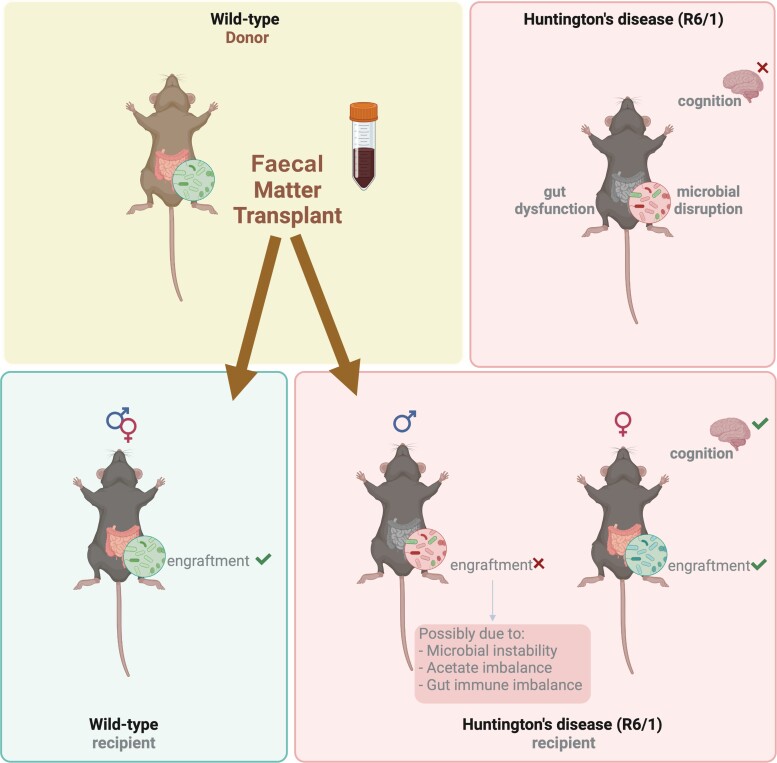

## Introduction

Huntington’s disease is a fatal neurodegenerative disorder for which there are currently no effective disease-modifying treatments.^1^ Huntington’s disease has a complex core symptomology, including motor deficits, cognitive and psychiatric symptoms, with devastating impacts on Huntington’s disease patients and their families.^2^ Huntington’s disease is caused by expansions of trinucleotide (CAG) tandem DNA repeats in the huntingtin (*HTT*) gene.^3^ The mutated HTT protein, containing an expanded polyglutamine tract, is expressed ubiquitously throughout the body, affecting both the brain and the periphery.^4^ Gastrointestinal (GI) dysfunctions are serious complications of Huntington’s disease and can include the presence of constipation, weight loss and nutrient deficiency, as well as impairment in gut structure, permeability and motility.^5–8^ R6/1 transgenic Huntington’s disease mice express the mutant human HTT transgene and provide an excellent preclinical model, exhibiting progressive cognitive, behavioural, cellular and molecular deficits closely modelling clinical Huntington’s disease,^9–11^ including the onset of gut dysfunction at early stages of the disease with the potential to worsen with disease progression.^12^

The community of microorganisms that colonize the gut, and their activity (gut microbiome), have been shown to influence brain function.^13^ Disruption of this microbial ecosystem occurs in various neurodegenerative conditions, including Parkinson’s and Alzheimer’s disease.^14,15^ More recently, gut microbiome disruption has been consistently shown in both preclinical and clinical Huntington’s disease,^8,16–19^ even before the onset of motor symptoms.^16,17^ Importantly, in Huntington’s disease gene expansion carriers (including symptomatic individuals), alterations to the gut microbiota have been demonstrated to be associated with inflammatory status,^19^ cognitive performance and clinical outcomes.^18^

Faecal microbiota transplant (FMT) from healthy donors has been shown to be an effective approach for the treatment of some diseases that affect the GI tract and that have gut microbial disruption as a pathological feature, such as *Clostridium difficile* infection^19^ and active ulcerative colitis,^20^ potentially by restoring the GI tract with protective effects of the commensal microbiota. Recent studies have also investigated FMT as an approach for brain disorders including autism spectrum disorder^21^ and Alzheimer’s disease.^22^

We hypothesize that interventions that ameliorate gut microbiome disruption will, in turn, be therapeutic in Huntington’s disease. In this study, we investigated whether FMT from wild-type (WT) into Huntington’s disease mice will be therapeutic, an approach that has shown promise for some other brain disorders but has never been tested in Huntington’s disease. We assessed FMT impacts on the onset and progression of various aspects of the disease in the R6/1 transgenic mouse model of Huntington’s disease.

## Materials and methods

### Subject details

Male R6/1 hemizygous mice (originally from Jackson Laboratories, USA) after back-crossing onto the CBAxC57Bl/6 background for more than 10 generations (generating the CBAxC57Bl/6 strain background) were crossed with female CBAxC57Bl/6 F1 mice to generate male and female WT and R6/1 (Huntington’s disease) littermates. Males and females were bred and housed separately, and therefore, sex was not added as a factor of the study. Genomic DNA from a tail biopsy was used to characterize genotypes. Due to their coprophagic nature, mice were housed according to genotype, sex and treatment to avoid any sharing of microbiota as ingestion of cage-mate faeces can modulate the gut microbiota (2–5 mice per cage). Mice were housed in open-top cages (34 × 16 × 16 cm) with basic sterilized wood shavings and facial tissues for bedding and nesting materials. Cages are sterilized through a washer at 82°C before use.

All mice had *ad libitum* access to sterilized food and filtered water (through a 0.5 μm filter) and were housed in a room with a 12:12 h light/dark cycle, controlled for temperature (22°C) and humidity (45%). Cages were changed and body weight assessment was performed weekly. All experiments and procedures were approved by The Florey Institute of Neuroscience and Mental Health Ethics Committee and were performed following the research guidelines and regulations of the National Health and Medical Research Council.

### Preparation of donor caecal content for faecal microbiota transplantation

Sex- and age-matched WT littermate mice, naive to behavioural experiments and group housed (with their donor group mates) were used as faecal donors. Donors were culled via cervical dislocation, and caeca were harvested immediately and transferred to 15% glycerol–phosphate-buffered saline (PBS). The subsequent processing was performed under anaerobic conditions (10% CO_2_, 10% H_2_, 80% N_2_), following a previously published protocol with minor modifications.^23^ Caecal contents were removed from caecal tissue, weighed, resuspended in 4× (w/v) anaerobic PBS, and homogenized by vortexing. The resulting suspension was passed through a Falcon 100 μM nylon cell filter (Thermofisher Scientific, Waltham, USA) to obtain the non-fibrous content. The supernatant from all collected caeca was pooled, mixed with an equal volume of 30% anaerobic glycerol–PBS and stored in three Hungate tubes (one tube per day of gavaging to avoid freeze-thaw cycles) at –80°C until required (the solution was prepared in the same week of gavaging, therefore not being stored for more than 7 days). Prior to oral gavage, pooled caecal supernatant was diluted with 2 × volume of anaerobic PBS (pH: 7.2) resulting in a solution of 5 mg/ml of caecal content.

### Experimental models

**Figure 1:**
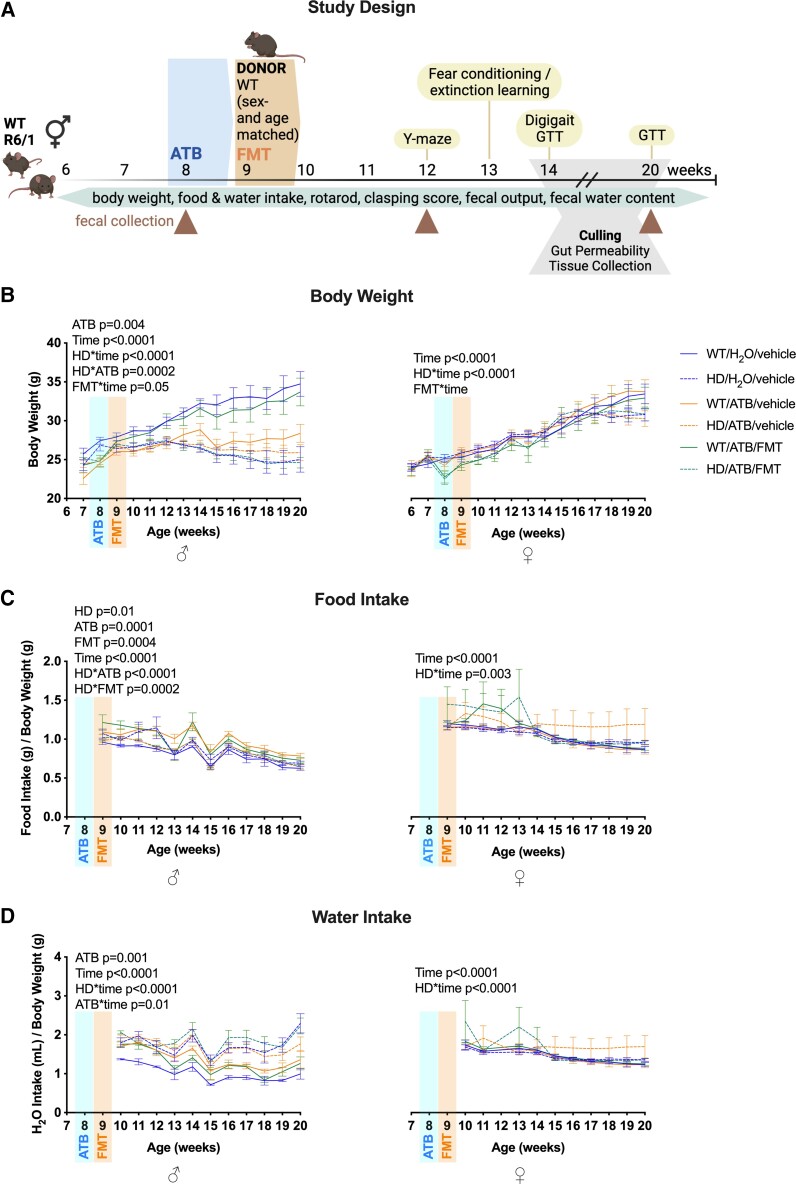
**Effects of ATB and ATB/FMT on the body weight, food and water intake of Huntington’s disease mice.** (**A**) Study design illustrating weekly measurements, motor and GI measurements and behavioural testing, as well as ATB intervention at 8 weeks and FMT intervention at 9 weeks. (**B**) Body weight (g) in WT and Huntington’s disease mice with ATB and ATB/FMT interventions from 7 to 20 weeks of age (males *n* = 8–26, females *n* = 6–14). (**C**) Food intake in WT and Huntington’s disease mice with ATB and ATB/FMT interventions from 9 to 20 weeks of age as gram-to-gram ratio (males *n* = 3–6 cages, females *n* = 2–7 cages; 2–3 mice per cage). (**D**) Water intake in WT and Huntington’s disease mice with ATB and ATB/FMT interventions from 10 to 20 weeks of age as millilitre-to-gram ratio (males *n* = 4–5 cages, females *n* = 2–7 cages; 2–3 mice per cage). Data and the line graphs represent mean ± SEM. (*B*–*D*) LMM with time as fixed effect. WT, wild-type; ATB, antibiotics; FMT, faecal microbiota transplant; LMM, linear mixed model.

To prepare the host for the subsequent FMT,^23^ antibiotics (ATBs) were used to induce a disruption of the gut microbiota.^24^ Mice were randomly assigned into either control (vehicle/vehicle), ATB-only (ATB/vehicle) or ATB/FMT groups (Fig. 1A). The ATB group had *ad libitum* access to a non-absorbable ATB cocktail of ertapenem sodium, vancomycin hydrochloride (both from Glentham Life Sciences) and neomycin sulphate (Sigma-Aldrich) in sterile water at a ratio of 1 mg/ml, for 7 days (at Week 8 of age), following a previously published protocol.^24^ The vehicle of ATB intervention was drinking water. Non-absorbable ATBs have a relatively small degree of systemic absorption, decreasing possible off-target effects. The FMT intervention consisted of oral administration of 150 µl of the prepared 5 g/ml of donor caeca content solution, for 3 days with 2 days of spacing (at 9 weeks of age). The vehicle control for the FMT intervention was oral administration of 7.5% glycerol–PBS. Plastic needles were used to minimize the effect of the gavage on the mice. The control group received neither ATB nor FMT, only the respective vehicle solution (drinking water and 7.5% glycerol–PBS).

### Food and water intake

Food and water intake were assessed from 6 to 20 weeks of age (Fig. 1A). To account for individual weight variability, intake was normalized to body weight and results represent gram of food per gram of body weight and millilitre of water per gram of body weight.

### Motor testing

#### Rotarod

Rotarod testing was used as an indicator of motor coordination.^25^ The Rotarod (Ugo Basile, Varese, Italy) consists of a motorized rotating cylinder that is divided into five compartments. One mouse is placed into each compartment, and the rotating cylinder accelerates at a constant speed of 4 rpm before the cylinder gradually accelerates to 40 rpm over 300 s. The time taken for the mice to fall onto the lever below is recorded as an indicator of motor coordination. Mice were tested weekly from 7 to 20 weeks of age after habituation at Week 6 (Fig. 1A). Habituation involved placing them on the cylinder at a constant speed of 4 rpm, before accelerating to 40 rpm across 300 s. Mice that clung to the rotating rod or changed orientation during the test, not turning back and/or getting back to the upright walk for three consecutive turns, were removed from the apparatus, and the maximum score was assigned.

#### Clasping

Mice were assessed weekly for clasping as video a phenotypic characteristic specific to Huntington’s disease transgenic mice (relative to their WT littermate controls) independent of motor coordination^11^ from 7 to 20 weeks of age (Fig. 1A). Clasping is identified by the retraction of paws during tail suspension and appears independent of deficits in motor coordination and locomotion. Mice are suspended by their tails for 30 s per session, while observing the clasping action of the paws during this time. Mice were scored on a 5-point scale from 0 to 4, with a score of zero indicating no clasping and a score of 1–4 indicating the number of clasping paws, with the highest score recorded. The experimenter was blinded to experimental groups during scoring.

#### Digigait

At 14 weeks of age, mice were assessed using Digigait (Mouse Specifics Inc., Boston, MA, USA) for the assessment of gait and locomotion^26^ (Fig. 1A). Mice are placed inside a plexiglass container on top of a transparent treadmill and allowed to habituate for 1 min. Mounted below the belt is a digital video camera that records the mice’s paws during treadmill locomotion. The treadmill was accelerated to 15 cm/s. Once the mouse was walking consistently for 2–5 s, 4–10 footsteps (or 3 s walking) were recorded and analysed, after which the mouse was removed from the testing chamber and returned to its home cage.

Analysis software was used to determine when individual paws are in contact with the belt of the treadmill to calculate several gait parameters. The gait parameters analysed were propelling time (the duration between maximum paw contact to the start of the swing phase) and braking time (the duration between the beginning of the swing phase and returning to maximum paw contact with the belt). The propel-to-brake ratio was then calculated.

### Behavioural testing of cognitive function

#### Y-maze

For cognitive assessments, we used Y-maze as a short-term spatial learning and memory test^27^ (Fig. 3A) at 12 weeks of age. The Y-maze is composed of three arms (10 cm wide, 30 cm long and 17 cm high) with visual cues at the end of each arm. Mice were housed in the testing room for at least 1 h before the test (for all behavioural tests). In the initial trial, one arm of the maze was closed (novel arm) using a divider, and mice were placed in another arm (home arm) and allowed to explore the home and another available arm (familiar arm) for 10 min. After the initial trial, mice were placed in a holding cage for 1 h and following this interval the mice were returned to the Y-maze for the second test trial, where they had free access to all three arms of the maze for 5 min. Tracking of animal movements was performed using Topscan Lite (CleverSys Inc., Reston, VA, USA) tracking software, and the time spent exploring the novel arm was recorded as a measure of short-term memory.

#### Fear conditioning and extinction learning

At 13 weeks of age, the mice underwent fear conditioning to assess their learning capabilities in concurrence with extinction learning, testing longer-term associative cognitive function^28^ (Fig. 3C). Fear conditioning and extinction learning were conducted as previously described.^28,29^ The mice were placed individually into fear conditioning chambers (Med Associates Inc., Fairfax, VT, USA) with stainless steel rod floors. The chambers contained either a patterned background and paper towel bedding or plain background and clean bedding, the same type used in the home cages, to create different environments. For fear conditioning, a conditioned stimulus (CS) of an auditory tone (80 dB, 5000 Hz, 10 s) was paired with an unconditioned stimulus (US) of a foot shock (electric shock, 0.6 mA, 1 s). The mice were habituated for 2 min, while their freezing (locomotor immobility) was measured (this period was considered the conditioning baseline), then the mice received six paired CS–US with the tone lasting 10 s and co-terminating with a 1 s foot shock. The inter-trial interval was 110 s. Freezing was analysed during the 9 s before the onset of the foot shock to ensure the freezing was a conditioned response to the tone. Following the last presentation of the tone, the mice were left within the chamber for 2 min before being returned to their home cages.

The following day their memory of the CS was tested in a different environmental context within the experimental chamber, to ensure the chamber environment was not paired with the tone in the CS. Mice were allowed to habituate in the chamber for 2 min, while their freezing measurement was analysed (this period was considered the extinction baseline) before being exposed to 45 presentations of the 10 s tone without the shock with an inter-trial interval of 10 s. Percentage freezing during the 10 s tone presentation was analysed and presented as averages of nine blocks of five CSs to track extinction learning. The recorded freezing of the mice during fear conditioning and extinction learning was analysed using VideoFreeze (Med Associates Inc.).

### GI measures

#### Faecal output and faecal water content

We analysed faecal water content as a measure of GI water absorption and faecal output and gut transit time markers of gut function and motility.^17^ At 12 weeks of age, mice were single-housed in a sterile cage for 1 h and the number of excreted pellets was recorded as faecal output. These pellets were collected, and the total weight was recorded before being dried at 95°C for >3 h.^17^ The difference between the initial faeces weight and the dry weight was recorded as a percentage and taken as the faecal water content.

#### Gut transit time

At 12 weeks of age, non-fasting mice were gavaged with non-absorbable carmine red dye (Sigma-Aldrich), prepared as a 6% (w/v) dilution in 0.5% methylcellulose (Sigma-Aldrich), autoclaved and filtered before administration. Mice were single-housed, and the time taken from gavage to the first appearance of carmine red was recorded as the GI transit time.

#### Gut permeability

At 14 and 20 weeks of age, mice were fasted for 4 h, then orally gavaged with 150 μl of 4 kDa fluorescein isothiocyanate (FITC) dextran (Sigma-Aldrich) dissolved in PBS to a concentration of 100 mg/ml to measure intestinal epithelial barrier permeability.^30^ Blood was collected via cardiac puncture 4 h after administration and immediately transferred to an EDTA collection tube and centrifuged at 1000 × g for 10 min. Plasma was then collected, and fluorescence was quantified at an excitation wavelength of 485 nm and an emission wavelength of 528 nm (PHERAstar *FSX*, Millipore). FITC-Dextran serially diluted in PBS was used to calculate a standard curve.

#### Macroscopic measures

Gut macroscopy, including caecum and colon measurements, was assessed as an indicator of general gut health.^7^ At 14 and 20 weeks of age, mice were euthanized by cervical dislocation; the intestines were removed and placed on a non-absorbent surface, and the length of the caecum and colon was measured using a ruler. The caecum was then weighed and normalized to body weight (g).

### Short-chain fatty acid and branched-chain fatty acid extraction and analysis

Mouse plasma samples were taken at 14 and 20 weeks of age and stored at –80°C prior to analysis. The extraction of short-chain fatty acids (SCFAs) and branched-chain fatty acids (BCFAs) was performed using a modified protocol.^31^ Briefly, 20 µl of plasma was suspended in 380 µl of water:acetonitrile (1:1, v/v) containing 4 µM of 4-methylvaleric acid (product # 277827, Sigma-Aldrich, Australia) as internal standard. Samples were vortexed for 30 s and then mixed at 950 rpm for 10 min at 4°C with a thermomixer (Eppendorf, Macquarie Park, Australia). Samples were centrifuged at 16 200 × g (Beckman Coulter Microfuge 22R refrigerated microcentrifuge) for 5 min at 4°C, and the supernatant was transferred to fresh LoBind Eppendorf tubes. Stock solutions of all the SCFAs and BCFAs were freshly and individually prepared in 100% aqueous acetonitrile with 1 mM concentration. This solution was further diluted to have concentrations of 50 to 0.1 µM and the resulting solutions were used as the calibrators. The calibrators (40 µl) and sample supernatant (40 µl) were mixed with 20 µl of 200 mM 3-nitrophenylhydrazine (Product # N21804, Sigma-Aldrich) in 50% aqueous acetonitrile and 20 µl of 120 mM 1-Ethyl-3-(3-dime-thylaminopropyl) carbodiimide (EDC, Product # E7750, Sigma-Aldrich) in 50% aqueous acetonitrile with 6% pyridine solution. The mixture was reacted at 40°C for 30 min in a thermomixer at 950 rpm. The reaction was stopped using 20 µl of 200 mM quinic acid (Product # 46944, Sigma-Aldrich) dissolved in acetonitrile:water (1:1, v/v) by further incubating the mixture at 40°C for 30 min in a thermomixer at 950 rpm. Finally, the samples were then spiked with 20 µl of 10 µM isotope-labelled internal standard mix prepared in accordance with the previously described protocol.^31^ The samples were further diluted to 2 ml with 15% acetonitrile and 1 µl were then injected for LC-MS/MS analysis. Derivatized SCFAs and BCFAs were processed and detected by Metabolomics Australia (Bio21 Institute, Melbourne, VIC, Australia) using the conditions previously described^1,2^ using Agilent 1290 liquid chromatography (LC) system and Agilent Triple quadrupole 6490 mass spectrometer (Agilent Technologies, Mulgrave, Australia).

### Gut immune profile

Proximal colon samples from 14 and 20 weeks of age were stored at –80°C prior to analysis. Samples were sonicated in 300 µl cell lysis buffer (Invitrogen) and refrozen. The thawed suspension was spun for 5 min at 3000 rpm, and the supernatant was assayed for cytokines as per the manufacturer’s recommendations. The ProcartaPlex multiplex Immunoassay (Invitrogen) was used to determine the Th17 panel of cytokines (IFNγ, IL-17A, IL-17E, IL-1β, IL-21, IL-22, IL-6, IL-7R and TNF-α) using Luminex® 200 System. The DC protein assay kit (Bio-Rad laboratories, USA) was used to determine the protein content. Groups that had less than four samples above the limit of the detection were excluded from the analysis. Results were normalized to supernatant protein concentration and are expressed as pg/mg of protein.

### Faecal DNA extraction and 16S rRNA sequencing

Faecal samples were collected at 8 (pre-interventions), 12 (3 weeks post-ATB, 2 weeks post-FMT) and 20 (11 weeks post-ATB, 10 weeks post-FMT) weeks of age (Fig. 1A). Mice were placed in sterile individual cages for faecal pellet collection. Due to COVID-19 pandemic restrictions (lockdown) at the time, we could not collect faecal samples at 8 weeks of age from the female cohorts that underwent the full experimental design and received all the interventions. Faecal pellets from an extra cohort of 8-week-old WT and Huntington’s disease females were added to the study instead. Fresh faecal pellets were placed into 1.5 ml Eppendorf tubes aseptically, immediately frozen with dry ice, followed by storage at –80°C prior to analysis (up to 6 months with no freeze-thaw cycle). Faecal pellets underwent DNA extraction by a combination of mechanical and chemical lysis method with using a DNeasy PowerLyzer PowerSoil kit (QIAGEN, Hilden, Germany, according to the manufacturer’s instructions with modifications as previously described).^23^

Extracted DNA was used for amplicon sequencing of the V4 hypervariable region (515–806 bp) of the 16S rRNA gene on a Miseq Illumina platform as previously described.^32^ Paired-end sequencing (2 × 300 bp) of indexed amplicon libraries were performed using a MiSeq Reagent Kit v3 and MiSeq system (Illumina Inc., San Diego, USA) at the South Australian Genomics Centre (SAGC), Adelaide, Australia. Microbiota profiling was performed based on a subsampling depth of 9604 or 12 777 sequence reads for the male or female cohort, respectively.

### Bioinformatics analysis

Paired-end 16S rRNA gene sequence reads were analysed using QIIME2 (v2.0).^33^ Briefly, the DADA2 workflow was used for denoising, quality filtering, chimaera removal and merging of paired-end sequence reads.^34^ Taxonomic assignment of amplicon sequence variants (ASVs) was performed against the V4 hypervariable region sequences of the SILVA 132 16S rRNA reference database clustered at 97% similarity.^35^ Alpha diversity (observed species, Faith’s phylogenetic diversity) and weighted UniFrac distances were computed using QIIME2. Genus-level relative abundances were used for downstream analysis.

### Statistical analysis for 16S rRNA sequencing

Non-parametric analyses were performed using the Mann–Whitney test to compare two groups, or the Kruskal–Wallis test with *post hoc* Dunn’s test for three or more groups. Microbiota composition differences between groups were assessed based on weighted UniFrac distances and analysed using permutational ANOVA (PERMANOVA). Correction of multiple testing was performed using the false discovery rate (FDR) method. Sample ordination and analysis were performed using GraphPad Prism 9 or R (package vegan 2.5-7).

### Statistical analysis

GraphPad Prism (version 9.2.0) was used to plot the graphs, and the data analysis was performed using R studio (version 1.3.1093). Data are presented as the mean ± standard error of the mean (SEM). Linear mixed models (LMMs) were used to analyse the data separately for males and females. Cumulative LMM with Laplace approximation was used for clasping scores that was measured on an ordinal scale. For covariates declared significant at the 0.05 significance level, we performed a *post hoc* pairwise comparisons (emmeans R package) (Supplementary Tables 1–34).

In the LMM cage, effects were specified as random effects in LMM due to the potential effect of coprophagy. For single time point variables (e.g. brain weight), genotype and treatment (no treatment, ATB, ATB/FMT) were specified as fixed effects. For Y-maze, arm type (novel, familiar) was added as fixed effect. For repeated measurement variables (e.g. body weight), time was also added as a fixed effect with the analysis being conducted from Week 8, when interventions started. For contextual fear conditioning (CFC), baseline CFC conditioning and baseline CFC extinction were added as fixed effects, respectively. We considered all two-way interactions between fixed effects when there were at least four samples in each group combination, otherwise only the main effects were considered (as was the case for males for the acetate variable in Week 14).

### Data availability

All data, including 16S rRNA amplicon sequencing data, will be made fully available upon publication. Raw sequence data are deposited in the public repository Sequence Read Archive under the BioProject accession number PRJNA795813. The data analysis R code is available at https://github.com/SarithaKodikara/Fecal-microbiota-transplant-therapeutic-effect-in-a-Huntington-s-disease-mouse-model.

## Results

### ATB/faecal microbiota transplant did not improve motor deficits and related outcomes in Huntington’s disease mice

Impairment in motor performance, decreased body and brain weight and increased water intake are the most consistent markers of preclinical Huntington’s disease progression (Fig. 1A). Our study recapitulated all these phenotypic characteristics in the R6/1 transgenic mouse model of Huntington’s disease. When compared with WT, both male and female Huntington’s disease mice showed a decreased body weight over time (*P* < 0.0001) (Fig. 1B), despite an increasing food intake in Huntington’s disease males (*P* = 0.01) and Huntington’s disease females (*P* = 0.003) over time (Fig. 1C). In males, ATB decreased body weight (*P* < 0.0001). In Huntington’s disease mice, ATB increased body weight (*P* = 0.0002) and decreased food intake (*P* < 0.0001). ATB/FMT increased body weight in females over time (*P* = 0.005), and there was a trend to increase body weight in males over time (*P* = 0.052), as well as a significant increase in food intake in males (*P* = 0.0004). Conversely, ATB/FMT induced a decreased food intake (*P* = 0.0002). Both male and female Huntington’s disease mice showed an increase in water intake over time (*P* < 0.0001 and *P* < 0.0001, respectively), while ATB increased the water intake compared with the no treatment (no FMT nor ATB) mice (*P* = 0.01, in males only) over time (Fig. 1D).

**Figure 2:**
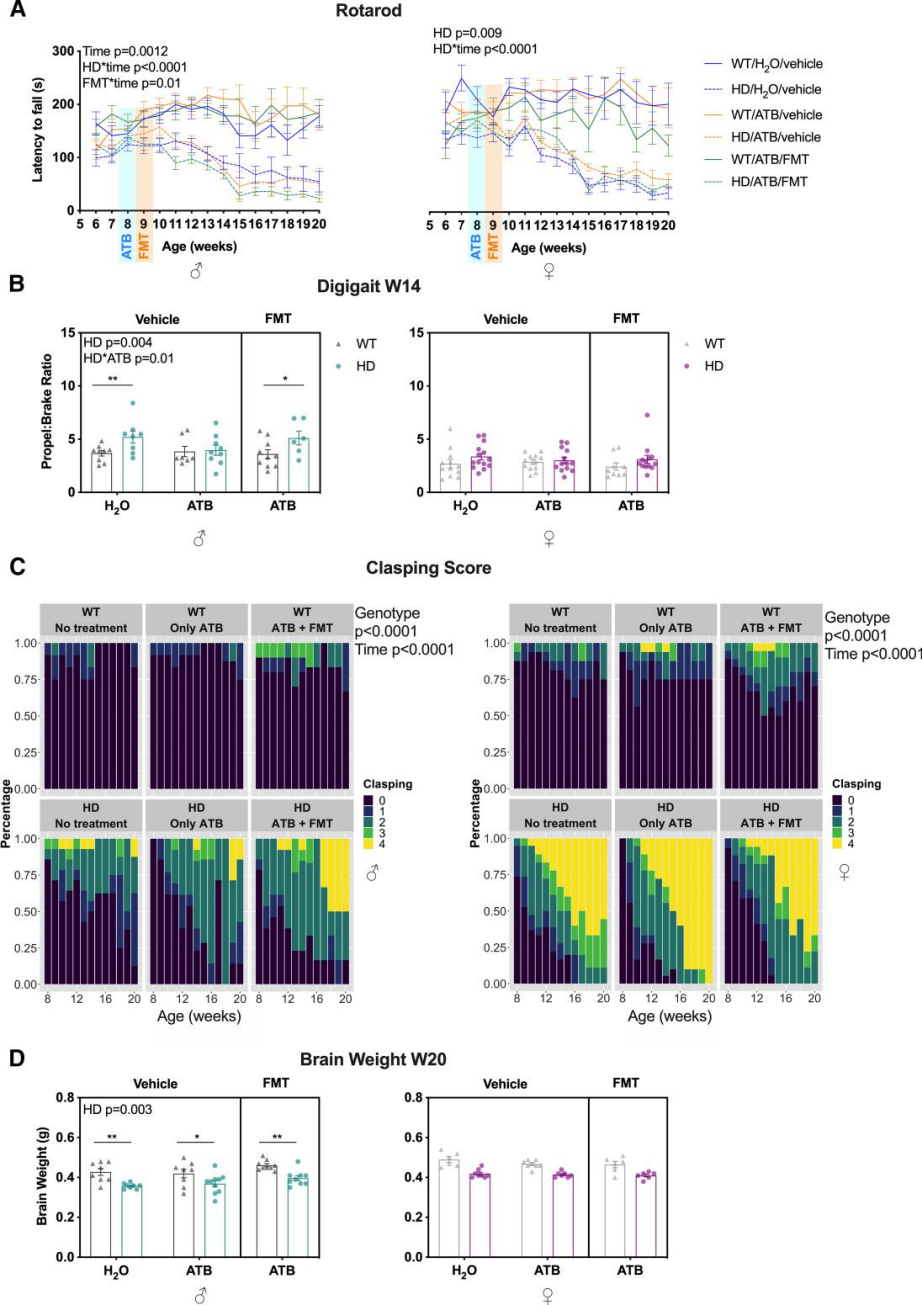
**Effects of ATB and ATB/FMT on the phenotypic expression of Huntington’s disease mice.** (**A**) Latency to fall (s) off rotarod in WT and Huntington’s disease mice with ATB and ATB/FMT interventions from 6 to 20 weeks of age (males *n* = 8–26, females *n* = 6–14). (**B**) Digigait propel:brake ratio in WT and Huntington’s disease mice with ATB and ATB/FMT interventions at 14 weeks of age (males *n* = 6–10, females *n* = 9–14). Each data point represents a mouse. (**C**) Clasping scores in WT and Huntington’s disease mice with ATB and ATB/FMT interventions from 8 to 20 weeks of age (males *n* = 8–19, females *n* = 6–14). (**D**) Brain weight (g) at 20 weeks of age for WT and Huntington’s disease mice with ATB and ATB/FMT interventions (males *n* = 8–10, females *n* = 6–8). Each data point represents a mouse. Data and the line graphs represent mean ± SEM. (**A**) LMM with time as fixed effect; (**B** and **D**) LMM followed by *post hoc* pairwise comparisons (emmeans) *P < 0.05, **P < 0.01; (**C**) cumulative LMM with Laplace approximation. WT, wild-type; ATB, antibiotics; FMT, faecal microbiota transplant; LMM, linear mixed model.

Both male and female Huntington’s disease mice showed an impaired motor performance on the rotarod over time (*P* < 0.0001 for both) (Fig. 2A). For both sexes, we observed an overall increase in clasping scores, a marker of Huntington’s disease progression, as the mice aged (Fig. 2C), as well as an increase in clasping score for Huntington’s disease mice compared with WT (*P* < 0.0001 for both) (Fig. 2C). Apart from an effect of ATB/FMT in males inducing an unexpected general decrease in the latency to fall off the rotarod over time (*P* = 0.0116), ATB and ATB/FMT were not able to modulate either rotarod or clasping performance. DigiGait analysis (assessment of gait and locomotion) in males only revealed an increase in propel:brake ratio in Huntington’s disease mice (*P* = 0.004) and a decrease in ATB-treated Huntington’s disease mice (*P* = 0.01) (Fig. 2B). Finally, at 20 weeks of age, a late stage in the disease, we observed a decrease in brain weight as a measure of gross neurodegeneration in Huntington’s disease males only (*P* = 0.003), with no effect of ATB or ATB/FMT (Fig. 2D).

### Faecal microbiota transplant modulated cognitive outcomes in Huntington’s disease

**Figure 3:**
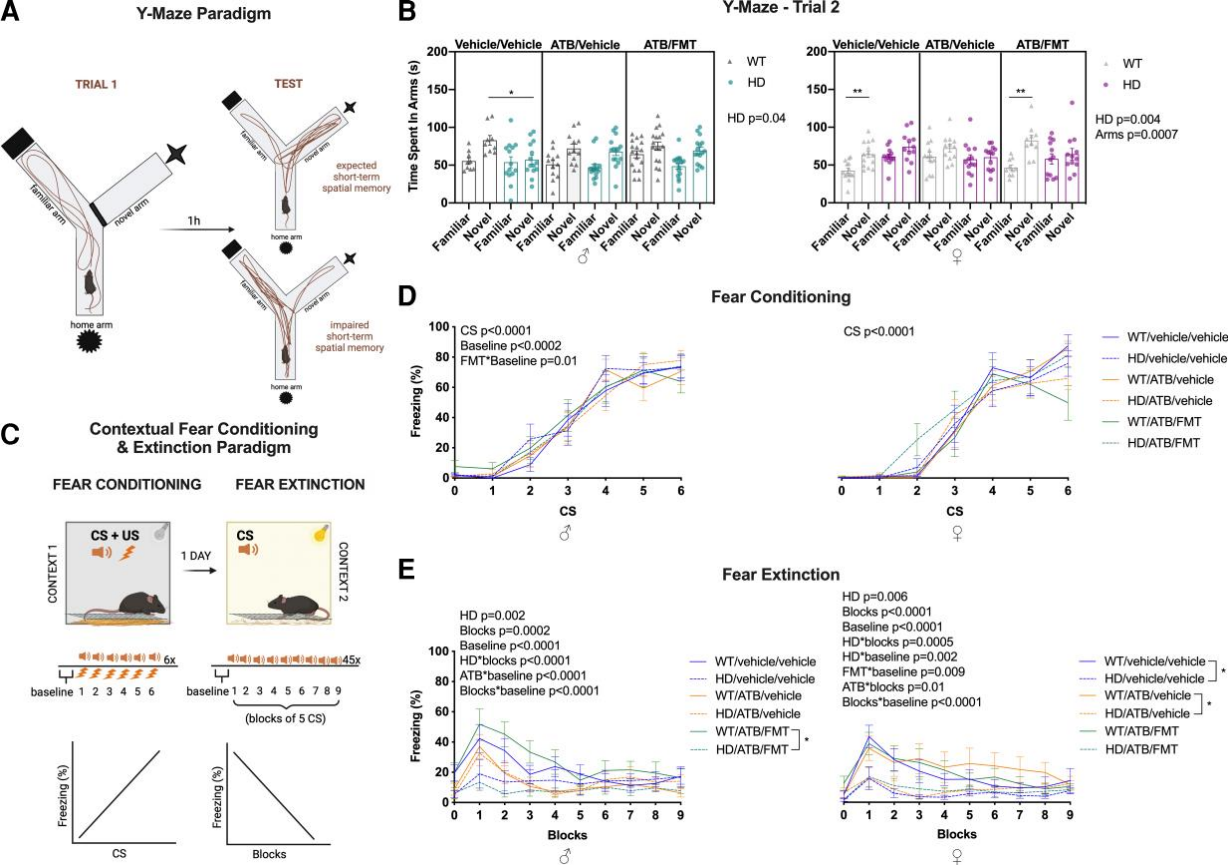
**Effects of ATB and ATB/FMT on the cognitive measures in WT and Huntington’s disease mice.** (**A**) Diagrammatic representation of the Y-Maze trial and testing paradigms. (**B**) Time spent in each of the Y-maze arms during the second trial in WT and Huntington’s disease mice with ATB and ATB/FMT interventions at 12 weeks of age. Each data point represents a mouse. (**C**) Diagrammatic representation of the CFC and extinction paradigm. (**D**) Freeze percentage during fear conditioning in WT and Huntington’s disease mice with ATB and ATB/FMT interventions (each data entry represents a mouse: males *n* = 11–15, females *n* = 10–13). (**E**) Percentage of freezing during fear extinction in WT and Huntington’s disease mice with ATB and ATB/FMT interventions (each data entry represents a mouse: males *n* = 7–16, females *n* = 10–13). Data and the line graphs represent mean ± SEM. (**B**) LMM followed by *post hoc* pairwise comparisons (emmeans) *P < 0.05, **P < 0.01; (**D** and **E**) LMM with time as fixed effect. WT, wild-type; ATBs, antibiotics; FMT, faecal microbiota transplant; LMM, linear mixed model.

Short-term spatial learning and memory were assessed based on Y-maze performance (Fig. 3A). As expected, both male and female Huntington’s disease mice performed worse on this cognitive test when compared with WT (*P* = 0.04 and *P* = 0.004, respectively) (Fig. 3B). Interestingly in females, *post hoc* testing indicated that in WT, the difference observed between arms in the control group is not present in the ATB group and appears again after the ATB/FMT treatment (Supplementary Table 1). On the other hand, ATB/FMT did not modulate Huntington’s disease performance in this test.

We also conducted contextual fear conditioning testing, by assessing the freezing response, or the absence of movement apart from respiration, as an indicator of cognitive function (Fig. 3C). In the conditioning trial we observed an effect of the CS in both males and females (*P* < 0.0001) indicating that the CS and US pairing induces conditioning learning (Fig. 3D). Furthermore, in males only, we saw an effect at the baseline time point (*P* = 0.0002), as well as an interaction between the baseline and ATB/FMT, overall decreasing the percentage of freezing, suggesting a positive effect of FMT on basal anxiety. At the extinction trial, we saw a decrease in the percentage of freezing in both males and females by time (*P* = 0.0002 and *P* < 0.0001, respectively), indicating that in general, the extinction occurred in the test (Fig. 3E). As previously reported, we found a decrease in extinction in both Huntington’s disease males (*P* < 0.0001) and females (*P* = 0.0005) over time compared with WT. We also observed an effect at the baseline time point in both males and females (*P* < 0.0001) with an increased percentage of freezing, while an interaction demonstrated a decrease in freezing over time in both males and females (*P* < 0.0001 and *P* = 0.01, respectively). Also, in female Huntington’s disease mice only, we observed a decrease in freezing behaviour at the baseline time point (*P* = 0.0005) and an interaction between ATB/FMT and the baseline (*P* = 0.009). ATB in females increased the percentage of freezing over time (*P* = 0.01). *Post hoc* analysis indicated that in males, there was a decrease in extinction in Huntington’s disease mice subjected to ATB/FMT when compared with WT mice subjected to ATB/FMT (Supplementary Table 2). In females, on the other hand, *post hoc* analysis indicated a decrease in extinction in Huntington’s disease mice in the control group and in the ATB group but not between the mice subjected to the ATB/FMT intervention (Supplementary Table 3).

### Gut dysfunction is a characteristic of the Huntington’s disease phenotype and ATB/faecal microbiota transplant did not modulate its progression

**Figure 4:**
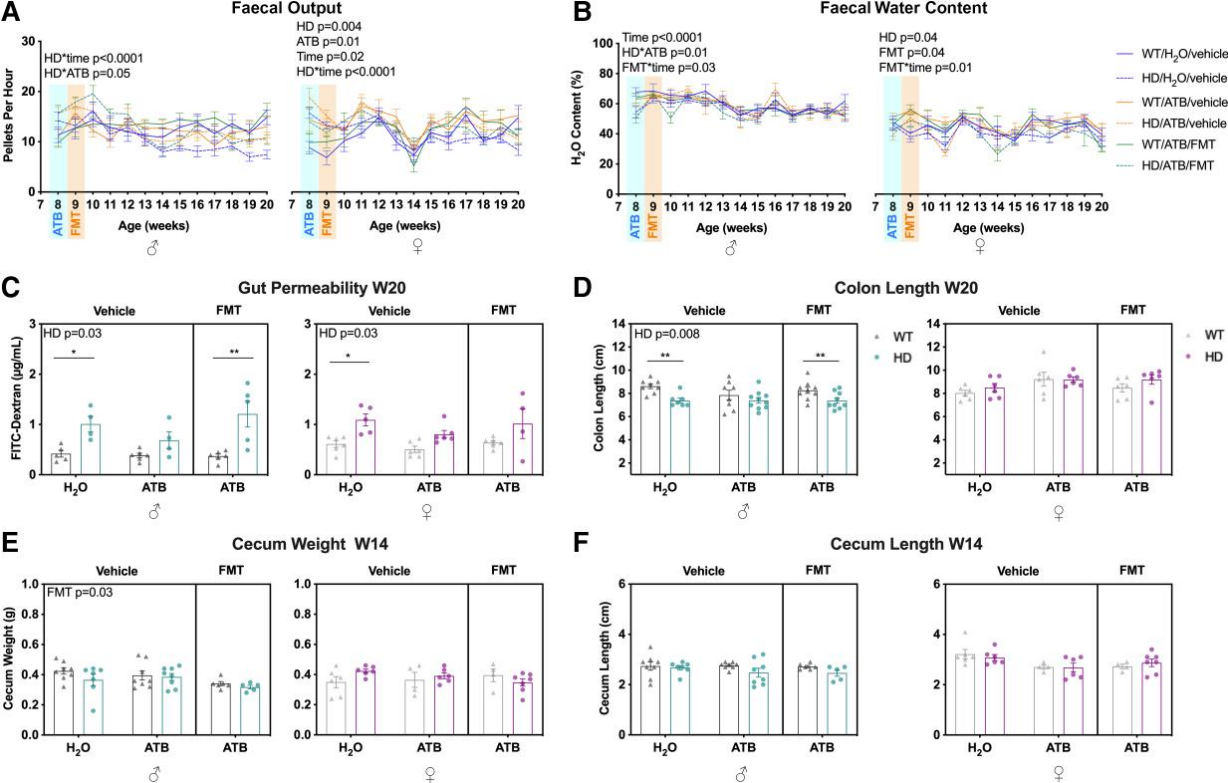
**Effects of ATB and ATB/FMT on GI measures in WT and Huntington’s disease mice at 14 and 20 weeks of age.** (**A**) Faecal output as pellet per hour in WT and Huntington’s disease mice with ATB and ATB/FMT interventions from 8 to 20 weeks of age (males *n* = 8–10, females *n* = 6–8). (**B**) Percentage faecal water content in WT and Huntington’s disease mice with ATB and ATB/FMT interventions from 9 to 20 weeks of age (males *n* = 7–11, females *n* = 5–8). (**C**) Gut permeability at 20 weeks of age measure using FITC-dextran (μg/ml) in WT and Huntington’s disease mice with ATB and ATB/FMT interventions (*n* = 4–6 for both males and females). (**D**) Colon length (cm) in WT and Huntington’s disease mice with ATB and ATB/FMT interventions at 20 weeks of age (males *n* = 7–10, females *n* = 6). (**E**) Caecum weight in (g) in WT and Huntington’s disease mice with ATB and ATB/FMT interventions at 14 weeks of age (males *n* = 5–8, females *n* = 4–7). (**F**) Caecum length (cm) at 14 weeks of age for WT and Huntington’s disease mice with ATB and ATB/FMT interventions (males *n* = 5–8, females *n* = 4–7). Each data point represents a mouse. Data and the line graphs represent mean ± SEM. (**A** and **B**) LMM with time as fixed effect; (**C**–**F**) LMM followed by *post hoc* pairwise comparisons (emmeans) *P < 0.05, **P < 0.01; WT, wild-type; ATB, antibiotics; FMT, faecal microbiota transplant; LMM, linear mixed model.

In the present study, we have investigated the gut structure and function of adult-onset R6/1 Huntington’s disease mice over time, including at early (14 weeks of age) and late (20 weeks of age) stages of the disease, while assessing the effect of ATB and ATB/FMT on these outcomes. Despite observing an increased food and water intake in Huntington’s disease mice compared with WT, both male and female Huntington’s disease mice showed a decreased faecal output, an indirect measurement of constipation as they aged (*P* < 0.0001, for both). In males we saw a trend towards ATB increasing faecal output in Huntington’s disease only (*P* = 0.056), while ATB increased the faecal output of both WT and Huntington’s disease females (*P* = 0.01) (Fig. 4A). Huntington’s disease females showed an overall decrease in faecal water content (*P* = 0.04). ATB increased the faecal water content in Huntington’s disease males (*P* < 0.0140) while ATB/FMT over time induced a decrease in faecal water content in both males (*P* = 0.03) and females (*P* = 0.01) (Fig. 4B). While the faecal output and faecal water content results indicate constipation in the Huntington’s disease mice, when gut transit time was assessed, apart from a trend of ATB/FMT to induce a decrease in the gut transit time in females (*P* = 0.054), no differences were observed at Week 14 or 20 for either sex (Supplementary Fig. 1A and B). Since a leaky gut has been identified in R6/2 Huntington’s disease mice,^8^ we investigated the intestinal permeability in our R6/1 Huntington’s disease model. No differences at the early disease stage (Week 14) in Huntington’s disease mice were observed, when compared with WT (Supplementary Fig. 1C), consistent with our recent findings.^12^ However, an increase in gut permeability at the late disease stage (Week 20) was detected in both male and female Huntington’s disease mice compared with WT littermate controls (*P* = 0.02 and *P* = 0.03, respectively) (Fig. 4C). ATB and ATB/FMT interventions were not able to modulate this outcome.

We investigated the gut macroscopic structure of the Huntington’s disease mice and found no genotype differences at 14 weeks of age (Fig. 4E and F, Supplementary Fig. 1D), indicating no gut gross structural changes at this early stage of the disease. However, at the late disease stage (20 weeks of age), we did observe a decreased colon length in Huntington’s disease males compared with WT littermate controls (*P* = 0.008) (Fig. 4D). Apart from male WT and Huntington’s disease mice showing a decrease in caecum weight induced by ABT/FMT treatment (*P* = 0.03) (Fig. 4E), we did not see any short-term or long-term effect of ATB or ATB/FMT on other gut outcomes, including caecum and colon length at Week 14 and caecum weight and length at Week 20 (Fig. 4F, Supplementary Fig. 1D–F). Altogether, these results indicate the presence of gut dysfunction in the late stage of the Huntington’s disease phenotype, as well as sexual dimorphism, with Huntington’s disease males being slightly more affected. Moreover, neither ATB nor FMT resulted in significant gut-related modulation.

### Huntington’s disease males develop gut microbiome disruption including abnormally high microbiome instability at an early stage of the disease

In determining the potential for early-stage prevention of gut microbiome disruption (via FMT intervention) prior to the onset of gut dysfunction, the gut microbiota of male and female Huntington’s disease mice were characterized at 8 weeks of age, using faecal 16S rRNA sequencing and bioinformatics. Reductions in faecal total bacterial load (Supplementary Fig. 2A), and changes to the structure and composition of the gut microbial community were observed for Huntington’s disease males but not in females. Specifically, alpha diversity measures of microbial richness (observed species, *P* = 0.002) (Supplementary Fig. 2B) and diversity were reduced (Faith’s phylogenetic diversity, *P* = 0.007) (Supplementary Fig. 2C), while the composition of the gut microbiota was significantly altered in Huntington’s disease males compared with WT littermate controls (PERMANOVA *P* = 0.0002) (Supplementary Fig. 2D). In contrast, gut microbiota alterations were less marked in Huntington’s disease females compared with WT. Specifically, alpha diversity indices of microbial richness (*P* = 0.613) and Faith’s phylogenetic diversity (*P* = 0.779), as well as the gut microbiota composition (PERMANOVA *P* = 0.634), were similar between Huntington’s disease and WT females.

Homogeneity of the microbiota composition among Huntington’s disease male and female mice at the early stage of disease (Week 8) was significantly lower compared with those of WT littermate controls (PERMDISP *P* = 0.042 and *P* = 0.034, respectively). These results were consistent with significantly larger microbiota variation within Huntington’s disease mice compared with the WT mice at Week 8 (males, *P* < 0.0001 and females, *P* = 0.046, respectively) (Supplementary Fig. 3), with a higher magnitude of variation observed for Huntington’s disease males [median (IQR) = 0.23 (0.14, 0.27)] compared with females [0.16 (0.09, 0.24)].

### Huntington’s disease male mice show a lack of engraftment of WT gut microbiota following faecal microbiota transplant intervention

**Figure 5:**
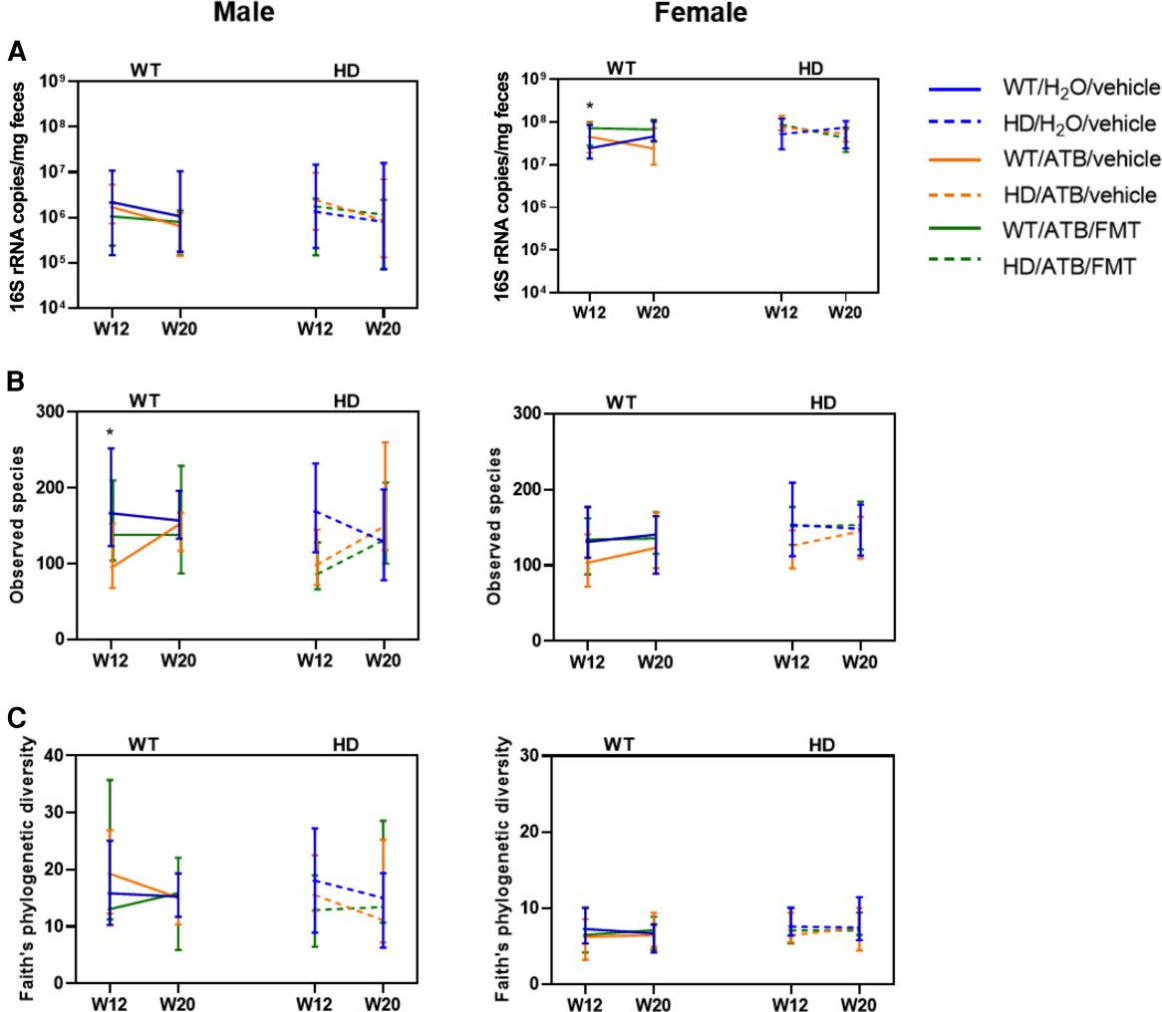
**Microbiome profiling reveals changes in gut microbiota over time.** Changes in (**A**) faecal total bacterial load, (**B**) microbial richness (observed species) and (**C**) microbial diversity (Faith’s phylogenetic diversity) between Week 12 and Week 20 across WT (each data entry represents a mouse: males *n* = 8, females *n* = 8) and Huntington’s disease groups (each data entry represents a mouse: males *n* = 8, females *n* = 7). The line graph represents the median and the error bars represent the interquartile ranges. Statistical analysis was performed using the Kruskal–Wallis test, followed by *post hoc* analysis using the Dunn’s test with FDR correction on multiple comparisons. Significance was determined based on FDR *P* < 0.05, as denoted by the asterisk. Legend for statistical comparisons (*): total bacterial load, W12: WT/H_2_O versus WT/ATB, FDR *P* = 0.039; observed species, W12: WT/H_2_O versus WT/ATB, FDR *P* = 0.005. WT, wild-type; ATB, antibiotics; FMT, faecal microbiota transplant.

FMT intervention was performed during the early stages of the disease to determine whether colonization of WT gut microbiota in Huntington’s disease mice could delay the onset of disease and associated phenotypic features. Donor microbiota engraftment was subsequently determined by faecal microbiota analysis of WT and Huntington’s disease groups at Week 12 and Week 20. For both the WT and Huntington’s disease genotypes in males, faecal bacterial load of ATB or FMT mice was similar to those receiving the vehicle by Week 12 and Week 20 (Mann–Whitney test, *P* > 0.05) (Fig. 5A). In females, faecal bacterial load at Week 12 was significantly higher in WT and Huntington’s disease mice that received FMT (*P* = 0.039 and *P* = 0.002, respectively) when compared with the levels in the untreated WT group (Fig. 5A). Similar to males, the levels of faecal bacterial load in females at 20 weeks of age were similar to those receiving the vehicle.

**Figure 6:**
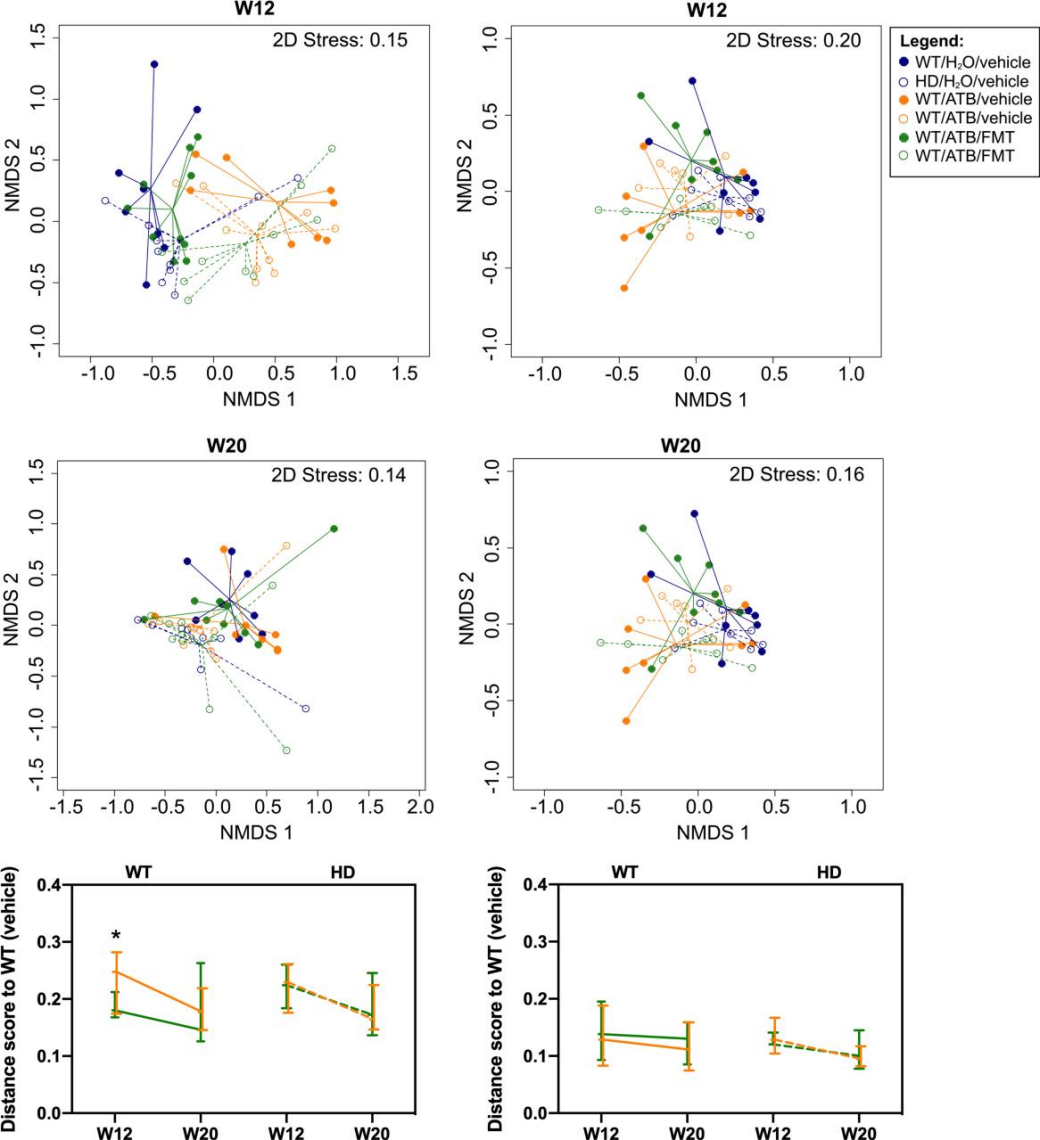
**Microbiota composition of WT and Huntington’s disease groups at Week 12 and Week 20.** (**A**) Non-metric multi-dimensional scaling plot depicting the ordination of samples of WT or Huntington’s disease mice receiving the vehicle, ATB or ATB/FMT at Week 12 and Week 20. Compositional distances between samples were determined based on weighted Unifrac distance scores. The solid and dotted lines connect the samples to the group centroid for the WT and Huntington’s disease groups, respectively, and the colour denotes the intervention groups. (**B**) Compositional differences of ATB and ATB/FMT groups compared with the untreated WT mice at Week 12 and at Week 20. Larger distance scores indicate larger compositional difference to the WT receiving the vehicle. The line graph represents the median and the error bars represent the interquartile ranges. Sample numbers: Week 12 males [vehicle *n* = 8 (WT), *n* = 8 (Huntington’s disease); ATB *n* = 9 (WT), *n* = 10 (Huntington’s disease); ATB/FMT *n* = 9 (WT), *n* = 10 (Huntington’s disease)], Week 20 males [vehicle *n* = 8 (WT), *n* = 8 (Huntington’s disease); ATB *n* = 8 (WT), *n* = 10 (Huntington’s disease); ATB/FMT *n* = 9 (WT), *n* = 10 (Huntington’s disease)], Week 12 females [vehicle *n* = 8 (WT), *n* = 8 (Huntington’s disease); ATB *n* = 8 (WT), *n* = 8 (Huntington’s disease); ATB/FMT *n* = 8 (WT), *n* = 8 (Huntington’s disease)], Week 20 females [vehicle *n* = 6 (WT), *n* = 8 (Huntington’s disease); ATB *n* = 8 (WT), *n* = 7 (Huntington’s disease); ATB/FMT *n* = 6 (WT), *n* = 6 (Huntington’s disease)]. Statistical comparison between groups was performed using a permutational ANOVA (PERMANOVA), with significance determined based on *P* < 0.05 as denoted by the asterisk. Each data entry represents a mouse. Legend for statistical comparisons (*): W12: WT/H2O versus WT/ATB, PERMANOVA *P* = 0.004; W12: WT/ATB versus WT/FMT, PERMANOVA *P* = 0.0006. WT, wild-type; ATBs, antibiotics; FMT, faecal microbiota transplant.

For both WT and Huntington’s disease males that received ATBs, reductions in the number of species observed (*P* = 0.005 and *P* = 0.007, respectively) (Fig. 5B) and compositional alterations (PERMANOVA *P* = 0.004 for both WT and Huntington’s disease genotypes) (Fig. 6A) were observed at 12 weeks of age when compared with those that received the vehicle. FMT reversed these effects in WT males (microbial richness, *P* = 0.366; composition, PERMANOVA *P* = 0.445) but not in Huntington’s disease males (microbial richness, *P* = 0.0009; composition, PERMANOVA *P* = 0.004) (Figs 5B and 6B). At 20 weeks of age, microbial alpha diversity of Huntington’s disease mice that received ATBs or FMT did not significantly differ from the WT mice (Fig. 5B and C), and compositional differences associated with gut dysbiosis almost disappeared (ATB *P* = 0.049; FMT *P* = 0.040) (Fig. 6A). In contrast, FMT was able to fully restore the gut microbiome composition of ATB-treated female Huntington’s disease mice to resemble that of the untreated WT mice (*P* > 0.05). Together, these results suggest that WT gut microbiota engraftment in Huntington’s disease mice was more effectively established in females compared with males.

### Huntington’s disease males have increased plasma acetate and colon IL-7R levels while female Huntington’s disease mice show a decrease in colon IFNγ levels

**Figure 7:**
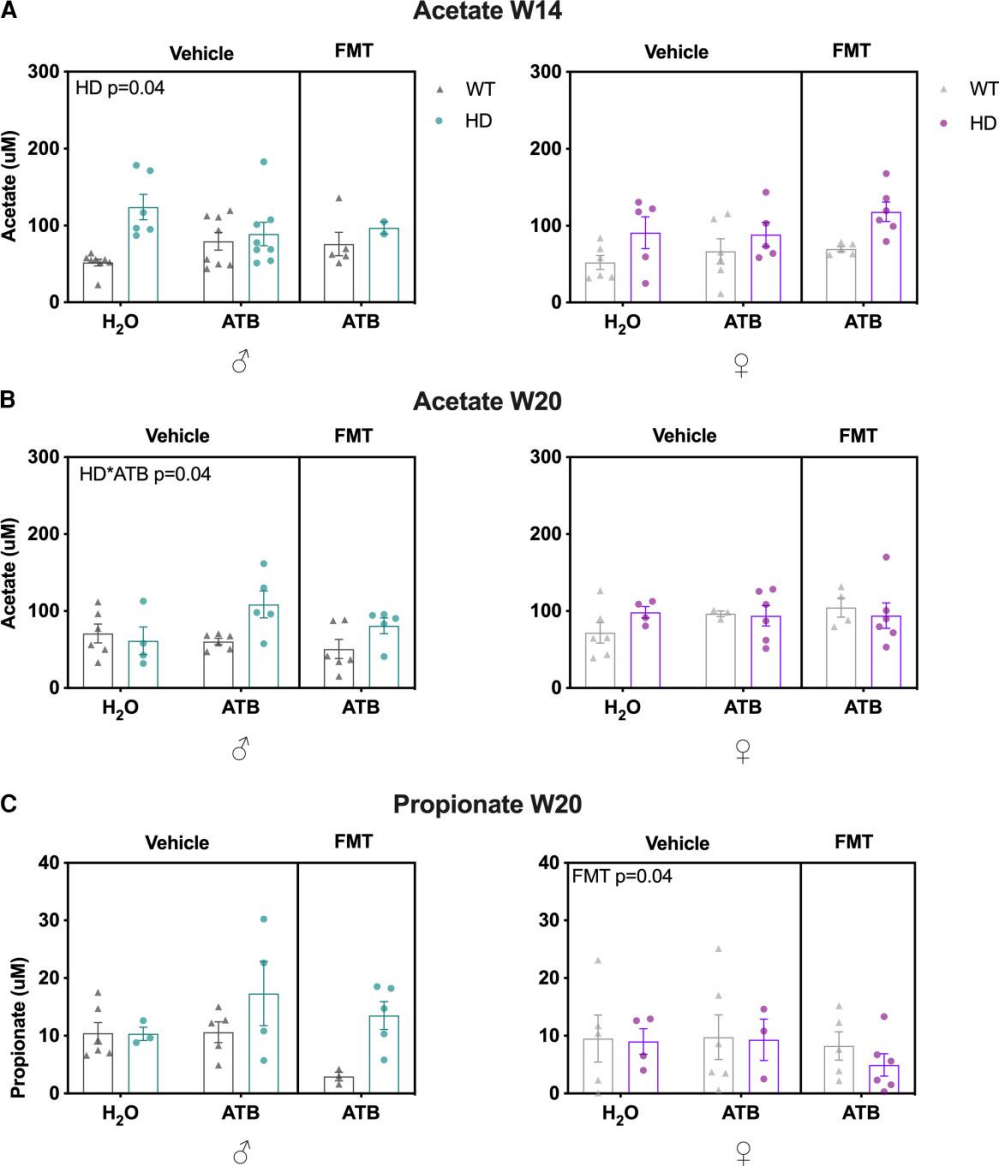
**Effects of ATB and ATB/FMT on SCFA concentrations in WT and Huntington’s disease mice at Week 14 and Week 20.** Acetate concentrations (μM) in WT and Huntington’s disease mice with ATB and ATB/FMT interventions at (**A**) 14 weeks of age (males *n* = 2–8, females *n* = 3–6) and (**B**) at 20 weeks of age (males *n* = 4–6, females *n* = 5–6). (**B**) Propionate concentrations (μM) in WT and Huntington’s disease mice with ATB and ATB/FMT interventions at 20 weeks of age (males *n* = 3–6, females *n* = 3–6). Each data point represents a mouse. Data represent mean ± SEM. (**A**–**C**) LMM. SCFAs, short-chain fatty acids; WT, wild-type; ATB, antibiotic; FMT, faecal microbiota transplant; LMM, linear mixed model.

We assessed the plasma levels of SCFAs and BCFAs, which are known to be produced by gut microbiota, and found an increase in acetate levels in Huntington’s disease males at 14 weeks of age compared with WT mice (*P* = 0.04) (Fig. 7A). At 20 weeks of age, we saw an increase in acetate levels induced by ATB in Huntington’s disease males (*P* = 0.04) (Fig. 7B) and an effect of ATB/FMT on propionate levels in females (*P* = 0.04) (Fig. 7C). We did not see any further significant difference in any other SCFA or BCFA levels (Supplementary Tables 23–26).

**Figure 8:**
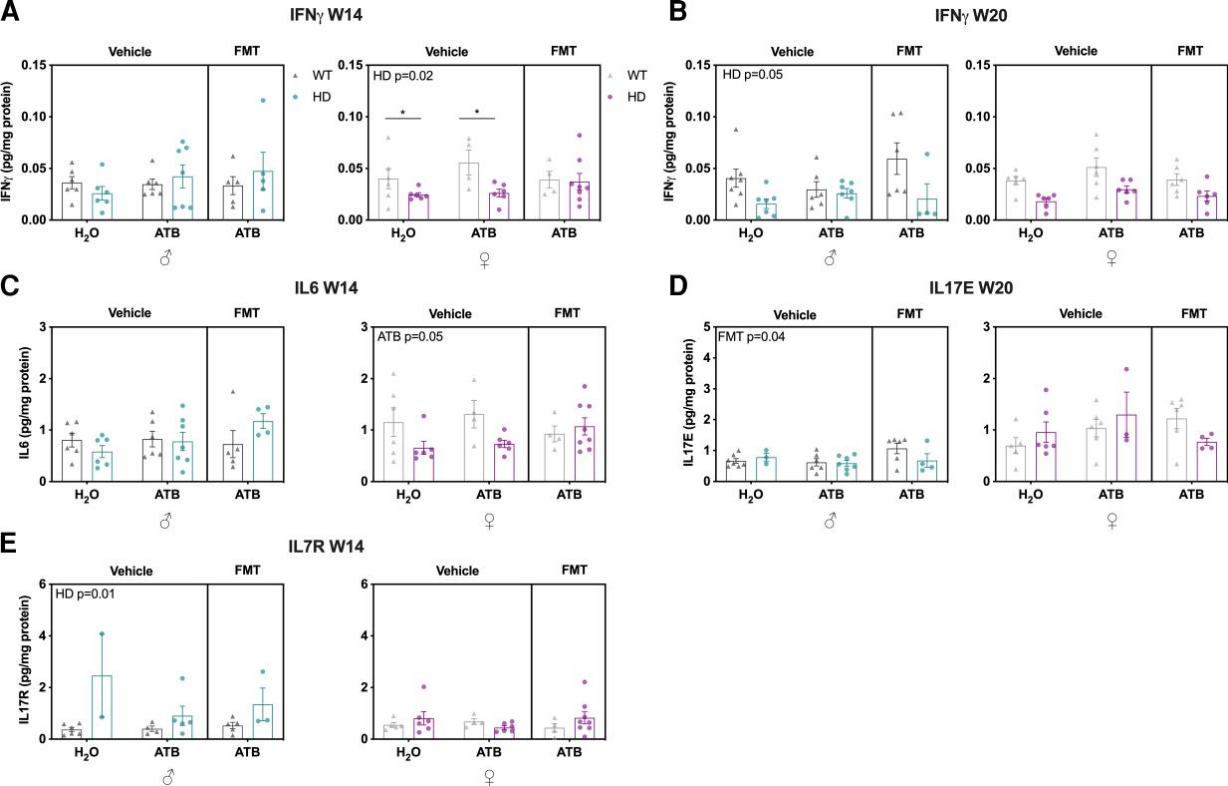
**Effects of ATB and ATB/FMT on gut inflammation in WT and Huntington’s disease mice at 14 and 20 weeks of age.** IFNγ (pg/mg protein) in WT and Huntington’s disease mice with ATB and ATB/FMT interventions at (**A**) 14 weeks of age (males *n* = 5–7, females *n* = 4–8) and (**B**) at 20 weeks of age (males *n* = 4–7, females *n* = 5–6). (**C**) IL6 (pg/mg protein) in WT and Huntington’s disease mice with ATB and ATB/FMT interventions at 14 weeks of age (males *n* = 4–7, females *n* = 4–8). (**D**) IL-17E (pg/mg protein) in WT and Huntington’s disease mice with ATB and ATB/FMT interventions at 20 weeks of age (males *n* = 2–7, females *n* = 3–6). (**E**) IL-7R (pg/mg protein) in WT and Huntington’s disease mice with ATB and ATB/FMT interventions at 14 weeks of age (males *n* = 2–6, females *n* = 4–8). Each data point represents a mouse. Data represent mean ± SEM. (**A**–**E**) LMM followed by *post hoc* pairwise comparisons (emmeans) *P < 0.05. WT, wild-type; ATBs, antibiotics; FMT, faecal microbiota transplant; LMM, linear mixed model.

We assessed the gut immune status by analysing cytokines levels in the proximal colon. Interestingly, at 14 weeks of age, we found a decrease in IFNγ levels in female Huntington’s disease mice (*P* = 0.02) (Fig. 8A) and, at 20 weeks of age, we saw a trend for male Huntington’s disease mice to have decreased IFNγ levels (*P* = 0.059) (Fig. 8B). In females, we found a trend towards a decrease of IL6 levels induced by ATB at 14 weeks of age (*P* = 0.059) (Fig. 8C). However, at 14 weeks of age in males, FMT increased IL-17E levels (*P* = 0.04) (Fig. 8D), and IL-7R was found to be increased in Huntington’s disease mice (*P* = 0.01) (Fig. 8E). No significant differences in levels of any other cytokine were observed (Supplementary Tables 27–34).

## Discussion

This is the first study to investigate the therapeutic potential of FMT as a means to shift the recently discovered pathological Huntington’s disease gut microbiome characteristics (dysbiosis) towards a healthy profile. FMT modulated cognitive outcomes in Huntington’s disease, an effect that was more pronounced in females. This study reveals an inefficiency of engraftment of the WT microbiota in Huntington’s disease male mice and an associated sexually dimorphic disruption and instability of the gut microbial community, circulating SCFA levels and gut immune profiles in Huntington’s disease. We report, for the first time, significant gut dysfunction in the late stage of the Huntington’s disease phenotype. Sexual dimorphism was also observed for this trait, with Huntington’s disease males being more affected. Our findings, together with those of others, suggest that Huntington’s disease is not just a brain disorder but rather is a whole-body disease with pathological features including sexually dimorphic and progressive gut dysfunction and microbial disruption. These findings will inform future microbiota-targeted therapies for Huntington’s disease, to address both central and peripheral symptoms of this fatal disease.

The onset of cognitive symptoms in Huntington’s disease is a driving factor in the progressive decline in quality of life.^36^ Improvement in learning and memory has also been one of the key targets for the gut microbial interventions for brain disorders.^13,37,38^ Corroborating previous findings,^9^ we have found reduced novel arm preference in Huntington’s disease mice in the Y-maze cognitive test. GI inflammation and gut microbiome disruption have been shown to impair cognitive performance in the Y-maze,^39^ and in our study, ATB in female WT mice inhibited cognitive performance in the Y-maze, which was resolved after the FMT intervention. We have also replicated previous findings showing that Huntington’s disease mice exhibit decreased freezing in the extinction trial of the fear conditioning test, indicating impaired fear learning and memory.^40^ Recent findings have implicated the gut microbiota in fear memory and impaired extinction learning with ATB-treated and germ-free mice showing impaired extinction learning.^41^ We observed similar results in females with the ATB treatment. In males, we found a decrease in extinction induced by ATB/FMT in Huntington’s disease mice when compared with WT mice. However, in females, the FMT intervention was able to fully rescue the cognitive deficits observed in Huntington’s disease mice. Altogether, these results reflect the sexually dimorphic FMT engraftment observed in Huntington’s disease mice and support the hypothesis that interventions that ameliorate gut microbiome disruption (dysbiosis) could in turn be therapeutic for this disease.

Successful engraftment of the gut microbiota is dependent on many factors, including genetic background, composition of the gut microbial community, inflammation status, housing environment, as well as diet of the donor and recipient individuals.^42^ All variables besides the genetic and microbiota differences between our donor WT and recipient Huntington’s disease mice, were carefully controlled and kept consistent in the present study. We have also followed the most recent guidelines for reporting and performing animal FMT^43^ and have used established protocols for both the ATB gut preparation^24^ and intervention.^23^ FMT engraftment was successful in both WT males and females, whereas a lower fidelity of engraftment and subsequently, lack of phenotype modulation was observed in Huntington’s disease mice, particularly in Huntington’s disease males. These results suggest the importance of further understanding complex donor-recipient dynamics in Huntington’s disease for the optimization of future clinical intervention strategies associated with modulating microbial–host relationships.

The stability of the gut microbiome is critical to host health, in which complex interactions between microbial species increases resilience of the microbial community to perturbation.^44,45^ An unstable state involving increased heterogeneity in microbiome composition over time, which has been associated with numerous negative health outcomes,^46^ was observed in the present study, particularly in Huntington’s disease males, extending our previous study in unmanipulated R6/1 Huntington’s disease mice.^16^ Notably, in the present study, we observed that the instability of the Huntington’s disease gut microbiome was concurrent with its resistance to FMT engraftment. Given that the host genetics greatly influences the physical structure of the gut, which then determines the chemical and physical environment inhabited by the gut microbiome,^47^ one possible explanation is that the expression of mutant HTT (via the human Huntington’s disease transgene) by the Huntington’s disease mice altered the gut mucosal lining and overall architecture, thus exerting stochastic effects on microbiome composition while impeding the colonization of microbes adapted to a healthy gut. This hypothesis should be further investigated.

Male Huntington’s disease mice showed increased levels of acetate (a key SCFA) in plasma at early and late stages of the disease. The major source of circulating SCFAs is the fermentation of indigestible carbohydrates by intestinal bacteria,^48^ and SCFAs are considered key regulators of the microbiota–gut–brain crosstalk.^49^ While increases in SCFA levels are usually associated with beneficial outcomes, increased plasma acetate levels have been shown in multiple sclerosis patients compared with controls, with levels correlating with greater disability and increased T helper 17 (T_H_17) + cells.^50^ Interestingly, acetate is considered to be an important metabolite for host resistance to bacterial infection, having a pivotal role in the pro- and anti-inflammatory balance, with high acetate promoting superior immune control.^51,52^ The gut immune profile is crucial for the host gut microbiota composition,^53^ and it has been recently shown to be a key modulatory factor for the efficacy of FMT in *C. difficile* infection.^54^ We have found increased IL-7R colon levels in Huntington’s disease males, while Huntington’s disease females showed a decrease in IFNγ colon levels. Intestinal IL-7R signalling from the T_H_17 cell subset is present in inflamed colon tissue, closely related to inflammatory bowel disease.^55^ IFNγ mediates the recognition and response to bacteria,^56^ both key players in the host bacterial defence and in shaping the gut microbial community. The imbalance of the acetate levels and intestinal immunity observed in male and female Huntington’s disease mice (a more pro-inflammatory and antimicrobial defence profile in males) could also be related to the sexual dimorphism observed in the FMT engraftment in Huntington’s disease. Further studies are needed to test this hypothesis. Nevertheless, our results raise opportunities to improve FMT efficacy for therapeutic intervention for both sexes in Huntington’s disease by focusing on modulating gut microbial stability, acetate levels and immune profiles.

In conclusion, our results demonstrate a potential role for gut microbiota in modulating cognitive outcomes in Huntington’s disease, presenting opportunities to pursue novel microbiota-targeted therapies for this devastating disease in future preclinical studies and clinical trials.

## Supplementary Material

fcac205_Supplementary_Data

## Abbreviations

ATB =: antibiotic
BCFA =: branched-chain fatty acid
CFC =: contextual fear conditioning
CS =: conditioned stimulus
FITC =: fluorescein isothiocyanate
FMT =: faecal microbiota transplant
GI =: gastrointestinal
HTT =: huntingtin
LMMs =: linear mixed models
SCFA =: short-chain fatty acid
US =: unconditioned stimulus
WT =: wild-type
