# Supplementary material for: Faecal microbiota transplant ameliorates gut dysbiosis and cognitive deficits in Huntington’s disease mice
Source: Brain Commun. 2022 Aug 12;4(4):fcac205. doi: 10.1093/braincomms/fcac205 (PMC9400176; doi:10.1093/braincomms/fcac205)
Supplement: fcac205_Supplementary_Data [file fcac205_supplementary_data.zip › Supplementary figure legends.docx]

**Supplementary Figure Legends**

**Supplementary Figure 1** **Effects of ATB and ATB/FMT on gastrointestinal measures in WT and HD mice at 14 and 20 weeks of age.** Gut transit time (min) in WT and HD mice with ATB and ATB/FMT interventions at (**A**) 14 weeks of age (males n=7-8, females n=4-7) and (**B**) at 20 weeks of age (males n=7-8, females n=6-8). (**C**) FITC-Dextran (µg/mL) concentrations in WT and HD mice with ATB and ATB/FMT interventions at 14 weeks of age (males n=5-8, females n=4-6). (**D**) Colon length (cm) in WT and HD mice with ATB and ATB/FMT interventions at 14 weeks of age (males n=5-8, females n=4-7). (**E**) Cecum weight (g) in WT and HD mice with ATB and ATB/FMT interventions at 20 weeks of age (males n=7-10, females n=6). (**F**) Cecum length (cm) in WT and HD mice with ATB and ATB/FMT interventions at 20 weeks of age (males n=8-10, females n=6-7). Each data point represents a mouse. Data represent mean ± SEM. Panels A-F LMM. HD, Huntington’s disease; WT, wild-type; ATB, antibiotics; FMT, fecal microbiota transplant; LMM, linear mixed model.

**Supplementary Figure 2 Comparison of the gut microbiota of WT and HD mice in the early stages of disease at week 8.** (**A**) Fecal bacterial load determined based on quantitative PCR of the 16S rRNA gene, (**B**) microbial richness based on observed species and (**C**) microbial diversity based on Faith’s phylogenetic diversity between WT (each data point represents a mouse: males n=26, females n=8) and HD (each data point represents a mouse: males n=31, females n=7) males and females at week 8. **(D)** Non-metric multi-dimensional scaling plot of WT and HD male and female mice samples ordinated based on weighted Unifrac distances at week 8. The solid and dotted lines denote the connection of WT and HD samples to the respective group centroid. HD, Huntington’s disease; WT, wild-type; ATB, antibiotics; FMT, fecal microbiota transplant; PCR, polymerase chain reaction.

**Supplementary Figure 3** **Within-group weighted Unifrac distance to assess the variation in microbiota composition within WT and HD mice at week 8** (males n=8, females n=11). Microbiota composition was computed based on the weighted Unifrac distances. Higher weighted Unifrac distances denote larger within-group microbiota variation. Each data point represents the compositional distance score between two mice. HD, Huntington’s Disease; WT, wild-type.
