# Supplementary material for: Faecal microbiota transplant ameliorates gut dysbiosis and cognitive deficits in Huntington’s disease mice
Source: Brain Commun. 2022 Aug 12;4(4):fcac205. doi: 10.1093/braincomms/fcac205 (PMC9400176; doi:10.1093/braincomms/fcac205)
Supplement: fcac205_Supplementary_Data [file fcac205_supplementary_data.zip › Supplementary Tables.pdf]

Supplementary Table 1: Post-hoc results for genotype and arm given Treatment in Ymaze in female

contrast	Treatment	estimate	SE	df	t.ratio	p.value
WT Novel - HD Novel	No treatment	18.6328	6.1519	32	3.0288	**0.0237**
WT Novel - WT Familiar	No treatment	22.3397	6.2664	69	3.5650	**0.0036**
WT Novel - HD Familiar	No treatment	32.2559	7.4644	32	4.3213	**0.0008**
HD Novel - WT Familiar	No treatment	3.7069	7.4644	32	0.4966	0.9593
HD Novel - HD Familiar	No treatment	13.6231	6.0176	69	2.2639	0.1167
WT Familiar - HD Familiar	No treatment	9.9163	6.1519	32	1.6119	0.3865
WT Novel - HD Novel	Only ATB	12.1654	6.2319	32	1.9521	0.2273
WT Novel - WT Familiar	Only ATB	11.4686	6.2929	69	1.8225	0.2717
WT Novel - HD Familiar	Only ATB	14.9175	7.6010	32	1.9626	0.2232
HD Novel - WT Familiar	Only ATB	-0.6968	7.6010	32	-0.0917	0.9997
HD Novel - HD Familiar	Only ATB	2.7521	6.1654	69	0.4464	0.9701
WT Familiar - HD Familiar	Only ATB	3.4489	6.2319	32	0.5534	0.9449
WT Novel - HD Novel	ATB_FMT	7.6466	6.5573	32	1.1661	0.6521
WT Novel - WT Familiar	ATB_FMT	24.4781	6.6793	69	3.6647	**0.0027**
WT Novel - HD Familiar	ATB_FMT	23.4082	8.1203	32	2.8827	**0.0336**
HD Novel - WT Familiar	ATB_FMT	16.8315	8.1203	32	2.0728	0.1837
HD Novel - HD Familiar	ATB_FMT	15.7616	6.4032	69	2.4615	0.0752
WT Familiar - HD Familiar	ATB_FMT	-1.0699	6.5573	32	-0.1632	0.9984

Supplementary Table 2: Post-hoc results for genotype given Treatment in CFC Extinction in male

contrast	Treatment	estimate	SE	df	t.ratio	p.value
WT - HD	No treatment	0.8993	4.0707	41	0.2209	0.8263
WT - HD	Only ATB	1.6746	4.0536	41	0.4131	0.6817
WT - HD	ATB_FMT	10.5431	4.2334	41	2.4905	**0.0169**

Supplementary Table 3: Post-hoc results for genotype given Treatment in CFC Extinction in female

contrast	Treatment	estimate	SE	df	t.ratio	p.value
WT - HD	No treatment	9.8921	4.3878	31	2.2544	**0.0314**
WT - HD	Only ATB	14.8152	4.2791	31	3.4622	**0.0016**
WT - HD	ATB_FMT	6.4316	4.8933	31	1.3144	0.1984

Supplementary Table 4: Post-hoc results for genotype given Treatment in Rotarod in female

contrast	Treatment	estimate	SE	df	t.ratio	p.value
WT - HD	No treatment	119.7505	17.4210	32	6.8739	**0e+00**
WT - HD	Only ATB	99.4652	17.3779	32	5.7237	**0e+00**
WT - HD	ATB_FMT	70.4652	18.4903	32	3.8109	**6e-04**

Supplementary Table 5: Post-hoc results for genotype * Treatment in Fecal Water Content in female

contrast	estimate	SE	df	t.ratio	p.value
WT No treatment - HD No treatment	4.1612	2.0162	15	2.0639	0.3548
WT No treatment - WT Only ATB	-2.9555	2.0162	15	-1.4659	0.6892
WT No treatment - HD Only ATB	1.4598	2.0630	15	0.7076	0.9781
WT No treatment - WT ATB_FMT	-1.5672	2.1601	15	-0.7255	0.9756
WT No treatment - HD ATB_FMT	3.9833	2.1600	15	1.8441	0.4693
HD No treatment - WT Only ATB	-7.1167	1.8666	15	-3.8126	**0.0173**
HD No treatment - HD Only ATB	-2.7014	1.9171	15	-1.4091	0.7215
HD No treatment - WT ATB_FMT	-5.7284	2.0212	15	-2.8341	0.1055
HD No treatment - HD ATB_FMT	-0.1779	2.0211	15	-0.0880	1.0000
WT Only ATB - HD Only ATB	4.4153	1.9171	15	2.3031	0.2518
WT Only ATB - WT ATB_FMT	1.3883	2.0212	15	0.6869	0.9807
WT Only ATB - HD ATB_FMT	6.9388	2.0211	15	3.4331	**0.0355**
HD Only ATB - WT ATB_FMT	-3.0270	2.0680	15	-1.4637	0.6904
HD Only ATB - HD ATB_FMT	2.5235	2.0679	15	1.2203	0.8206
WT ATB_FMT - HD ATB_FMT	5.5505	2.1647	15	2.5641	0.1668

Supplementary Table 6: Post-hoc results Post-hoc results for genotype*Treatment given Time in Fecal Output in female

contrast	Time	estimate	SE	df	t.ratio	p.value
WT No treatment - HD No treatment	1	-4.0863	1.3321	15	-3.0675	0.0697
WT No treatment - WT Only ATB	1	-3.6540	1.3785	15	-2.6508	0.1445
WT No treatment - HD Only ATB	1	-6.3756	1.5162	15	-4.2050	**0.0081**
WT No treatment - WT ATB_FMT	1	-0.5487	1.4697	15	-0.3734	0.9988
WT No treatment - HD ATB_FMT	1	-4.6543	1.5832	15	-2.9397	0.0876
HD No treatment - WT Only ATB	1	0.4323	1.3892	15	0.3112	0.9995
HD No treatment - HD Only ATB	1	-2.2894	1.3122	15	-1.7447	0.5258
HD No treatment - WT ATB_FMT	1	3.5375	1.4865	15	2.3797	0.2239
HD No treatment - HD ATB_FMT	1	-0.5680	1.3963	15	-0.4068	0.9983
WT Only ATB - HD Only ATB	1	-2.7216	1.2634	15	-2.1543	0.3130
WT Only ATB - WT ATB_FMT	1	3.1053	1.3906	15	2.2330	0.2794
WT Only ATB - HD ATB_FMT	1	-1.0003	1.4890	15	-0.6718	0.9825
HD Only ATB - WT ATB_FMT	1	5.8269	1.5130	15	3.8511	**0.0160**
HD Only ATB - HD ATB_FMT	1	1.7214	1.4020	15	1.2278	0.8170
WT ATB_FMT - HD ATB_FMT	1	-4.1055	1.4088	15	-2.9142	0.0917
WT No treatment - HD No treatment	2	-3.4982	1.2968	15	-2.6975	0.1335
WT No treatment - WT Only ATB	2	-3.5466	1.3301	15	-2.6665	0.1407
WT No treatment - HD Only ATB	2	-5.6801	1.4338	15	-3.9617	**0.0130**
WT No treatment - WT ATB_FMT	2	-0.6496	1.4190	15	-0.4578	0.9970
WT No treatment - HD ATB_FMT	2	-4.1670	1.5015	15	-2.7753	0.1169
HD No treatment - WT Only ATB	2	-0.0484	1.3136	15	-0.0369	1.0000
HD No treatment - HD Only ATB	2	-2.1820	1.2612	15	-1.7300	0.5343
HD No treatment - WT ATB_FMT	2	2.8486	1.4086	15	2.0223	0.3751
HD No treatment - HD ATB_FMT	2	-0.6688	1.3430	15	-0.4980	0.9955
WT Only ATB - HD Only ATB	2	-2.1335	1.2261	15	-1.7400	0.5285
WT Only ATB - WT ATB_FMT	2	2.8970	1.3388	15	2.1638	0.3088
WT Only ATB - HD ATB_FMT	2	-0.6204	1.4104	15	-0.4399	0.9975
HD Only ATB - WT ATB_FMT	2	5.0305	1.4314	15	3.5143	**0.0304**

Supplementary Table 6: Post-hoc results Post-hoc results for genotype*Treatment given Time in Fecal Output in female *(continued)*

contrast	Time	estimate	SE	df	t.ratio	p.value
HD Only ATB - HD ATB_FMT	2	1.5131	1.3507	15	1.1203	0.8656
WT ATB_FMT - HD ATB_FMT	2	-3.5174	1.3755	15	-2.5572	0.1687
WT No treatment - HD No treatment	3	-2.9101	1.2673	15	-2.2963	0.2543
WT No treatment - WT Only ATB	3	-3.4392	1.2891	15	-2.6679	0.1404
WT No treatment - HD Only ATB	3	-4.9846	1.3626	15	-3.6582	**0.0232**
WT No treatment - WT ATB_FMT	3	-0.7504	1.3762	15	-0.5453	0.9931
WT No treatment - HD ATB_FMT	3	-3.6798	1.4311	15	-2.5712	0.1648
HD No treatment - WT Only ATB	3	-0.5291	1.2483	15	-0.4239	0.9979
HD No treatment - HD Only ATB	3	-2.0746	1.2180	15	-1.7033	0.5499
HD No treatment - WT ATB_FMT	3	2.1596	1.3414	15	1.6100	0.6048
HD No treatment - HD ATB_FMT	3	-0.7697	1.2976	15	-0.5931	0.9900
WT Only ATB - HD Only ATB	3	-1.5454	1.1948	15	-1.2935	0.7841
WT Only ATB - WT ATB_FMT	3	2.6888	1.2949	15	2.0764	0.3488
WT Only ATB - HD ATB_FMT	3	-0.2406	1.3426	15	-0.1792	1.0000
HD Only ATB - WT ATB_FMT	3	4.2342	1.3610	15	3.1110	0.0644
HD Only ATB - HD ATB_FMT	3	1.3049	1.3071	15	0.9983	0.9114
WT ATB_FMT - HD ATB_FMT	3	-2.9293	1.3476	15	-2.1737	0.3045
WT No treatment - HD No treatment	4	-2.3220	1.2438	15	-1.8669	0.4567
WT No treatment - WT Only ATB	4	-3.3318	1.2563	15	-2.6520	0.1442
WT No treatment - HD Only ATB	4	-4.2892	1.3046	15	-3.2878	**0.0465**
WT No treatment - WT ATB_FMT	4	-0.8513	1.3420	15	-0.6343	0.9864
WT No treatment - HD ATB_FMT	4	-3.1925	1.3739	15	-2.3237	0.2440
HD No treatment - WT Only ATB	4	-1.0098	1.1951	15	-0.8450	0.9539
HD No treatment - HD Only ATB	4	-1.9672	1.1832	15	-1.6625	0.5738
HD No treatment - WT ATB_FMT	4	1.4707	1.2868	15	1.1429	0.8559
HD No treatment - HD ATB_FMT	4	-0.8705	1.2613	15	-0.6902	0.9803
WT Only ATB - HD Only ATB	4	-0.9573	1.1699	15	-0.8183	0.9595
WT Only ATB - WT ATB_FMT	4	2.4805	1.2597	15	1.9691	0.4021
WT Only ATB - HD ATB_FMT	4	0.1393	1.2875	15	0.1082	1.0000
HD Only ATB - WT ATB_FMT	4	3.4379	1.3037	15	2.6371	0.1478
HD Only ATB - HD ATB_FMT	4	1.0966	1.2722	15	0.8620	0.9500
WT ATB_FMT - HD ATB_FMT	4	-2.3412	1.3256	15	-1.7662	0.5134
WT No treatment - HD No treatment	5	-1.7339	1.2267	15	-1.4134	0.7191
WT No treatment - WT Only ATB	5	-3.2244	1.2324	15	-2.6164	0.1530
WT No treatment - HD Only ATB	5	-3.5937	1.2615	15	-2.8487	0.1029
WT No treatment - WT ATB_FMT	5	-0.9521	1.3170	15	-0.7230	0.9760
WT No treatment - HD ATB_FMT	5	-2.7053	1.3315	15	-2.0317	0.3705
HD No treatment - WT Only ATB	5	-1.4905	1.1555	15	-1.2899	0.7860
HD No treatment - HD Only ATB	5	-1.8598	1.1578	15	-1.6063	0.6069
HD No treatment - WT ATB_FMT	5	0.7817	1.2463	15	0.6273	0.9871
HD No treatment - HD ATB_FMT	5	-0.9714	1.2346	15	-0.7868	0.9656
WT Only ATB - HD Only ATB	5	-0.3692	1.1517	15	-0.3206	0.9994
WT Only ATB - WT ATB_FMT	5	2.2723	1.2339	15	1.8415	0.4707
WT Only ATB - HD ATB_FMT	5	0.5191	1.2466	15	0.4165	0.9981
HD Only ATB - WT ATB_FMT	5	2.6415	1.2611	15	2.0947	0.3402
HD Only ATB - HD ATB_FMT	5	0.8884	1.2467	15	0.7126	0.9774
WT ATB_FMT - HD ATB_FMT	5	-1.7531	1.3096	15	-1.3387	0.7603
WT No treatment - HD No treatment	6	-1.1458	1.2164	15	-0.9420	0.9290
WT No treatment - WT Only ATB	6	-3.1170	1.2178	15	-2.5595	0.1680

Supplementary Table 6: Post-hoc results Post-hoc results for genotype*Treatment given Time in Fecal Output in female *(continued)*

contrast	Time	estimate	SE	df	t.ratio	p.value
WT No treatment - HD Only ATB	6	-2.8982	1.2349	15	-2.3468	0.2355
WT No treatment - WT ATB_FMT	6	-1.0530	1.3018	15	-0.8089	0.9614
WT No treatment - HD ATB_FMT	6	-2.2180	1.3055	15	-1.6990	0.5524
HD No treatment - WT Only ATB	6	-1.9712	1.1312	15	-1.7426	0.5270
HD No treatment - HD Only ATB	6	-1.7524	1.1422	15	-1.5342	0.6495
HD No treatment - WT ATB_FMT	6	0.0928	1.2213	15	0.0760	1.0000
HD No treatment - HD ATB_FMT	6	-1.0722	1.2184	15	-0.8800	0.9456
WT Only ATB - HD Only ATB	6	0.2188	1.1407	15	0.1919	1.0000
WT Only ATB - WT ATB_FMT	6	2.0640	1.2182	15	1.6943	0.5551
WT Only ATB - HD ATB_FMT	6	0.8990	1.2214	15	0.7360	0.9740
HD Only ATB - WT ATB_FMT	6	1.8452	1.2348	15	1.4943	0.6728
HD Only ATB - HD ATB_FMT	6	0.6801	1.2312	15	0.5524	0.9927
WT ATB_FMT - HD ATB_FMT	6	-1.1650	1.2999	15	-0.8962	0.9416
WT No treatment - HD No treatment	7	-0.5577	1.2129	15	-0.4598	0.9969
WT No treatment - WT Only ATB	7	-3.0096	1.2129	15	-2.4813	0.1908
WT No treatment - HD Only ATB	7	-2.2027	1.2259	15	-1.7967	0.4959
WT No treatment - WT ATB_FMT	7	-1.1538	1.2966	15	-0.8899	0.9432
WT No treatment - HD ATB_FMT	7	-1.7308	1.2966	15	-1.3348	0.7623
HD No treatment - WT Only ATB	7	-2.4519	1.1229	15	-2.1835	0.3002
HD No treatment - HD Only ATB	7	-1.6450	1.1370	15	-1.4468	0.7002
HD No treatment - WT ATB_FMT	7	-0.5962	1.2129	15	-0.4915	0.9958
HD No treatment - HD ATB_FMT	7	-1.1731	1.2129	15	-0.9672	0.9214
WT Only ATB - HD Only ATB	7	0.8069	1.1370	15	0.7097	0.9778
WT Only ATB - WT ATB_FMT	7	1.8558	1.2129	15	1.5300	0.6519
WT Only ATB - HD ATB_FMT	7	1.2788	1.2129	15	1.0544	0.8916
HD Only ATB - WT ATB_FMT	7	1.0488	1.2259	15	0.8555	0.9515
HD Only ATB - HD ATB_FMT	7	0.4719	1.2259	15	0.3849	0.9987
WT ATB_FMT - HD ATB_FMT	7	-0.5769	1.2966	15	-0.4449	0.9973
WT No treatment - HD No treatment	8	0.0304	1.2164	15	0.0250	1.0000
WT No treatment - WT Only ATB	8	-2.9022	1.2178	15	-2.3832	0.2227
WT No treatment - HD Only ATB	8	-1.5072	1.2349	15	-1.2205	0.8205
WT No treatment - WT ATB_FMT	8	-1.2547	1.3018	15	-0.9638	0.9224
WT No treatment - HD ATB_FMT	8	-1.2435	1.3055	15	-0.9526	0.9259
HD No treatment - WT Only ATB	8	-2.9326	1.1312	15	-2.5925	0.1592
HD No treatment - HD Only ATB	8	-1.5376	1.1422	15	-1.3461	0.7563
HD No treatment - WT ATB_FMT	8	-1.2851	1.2213	15	-1.0522	0.8925
HD No treatment - HD ATB_FMT	8	-1.2739	1.2184	15	-1.0456	0.8949
WT Only ATB - HD Only ATB	8	1.3950	1.1407	15	1.2230	0.8193
WT Only ATB - WT ATB_FMT	8	1.6475	1.2182	15	1.3524	0.7528
WT Only ATB - HD ATB_FMT	8	1.6587	1.2214	15	1.3580	0.7498
HD Only ATB - WT ATB_FMT	8	0.2525	1.2348	15	0.2045	0.9999
HD Only ATB - HD ATB_FMT	8	0.2637	1.2312	15	0.2141	0.9999
WT ATB_FMT - HD ATB_FMT	8	0.0112	1.2999	15	0.0086	1.0000
WT No treatment - HD No treatment	9	0.6185	1.2267	15	0.5042	0.9952
WT No treatment - WT Only ATB	9	-2.7948	1.2324	15	-2.2678	0.2654
WT No treatment - HD Only ATB	9	-0.8117	1.2615	15	-0.6434	0.9855
WT No treatment - WT ATB_FMT	9	-1.3555	1.3170	15	-1.0293	0.9008
WT No treatment - HD ATB_FMT	9	-0.7563	1.3315	15	-0.5680	0.9917
HD No treatment - WT Only ATB	9	-3.4133	1.1555	15	-2.9539	0.0855

Supplementary Table 6: Post-hoc results Post-hoc results for genotype*Treatment given Time in Fecal Output in female *(continued)*

contrast	Time	estimate	SE	df	t.ratio	p.value
HD No treatment - HD Only ATB	9	-1.4302	1.1578	15	-1.2353	0.8134
HD No treatment - WT ATB_FMT	9	-1.9740	1.2463	15	-1.5840	0.6202
HD No treatment - HD ATB_FMT	9	-1.3748	1.2346	15	-1.1135	0.8684
WT Only ATB - HD Only ATB	9	1.9831	1.1517	15	1.7219	0.5390
WT Only ATB - WT ATB_FMT	9	1.4393	1.2339	15	1.1664	0.8456
WT Only ATB - HD ATB_FMT	9	2.0385	1.2466	15	1.6353	0.5898
HD Only ATB - WT ATB_FMT	9	-0.5439	1.2611	15	-0.4313	0.9977
HD Only ATB - HD ATB_FMT	9	0.0554	1.2467	15	0.0444	1.0000
WT ATB_FMT - HD ATB_FMT	9	0.5993	1.3096	15	0.4576	0.9970
WT No treatment - HD No treatment	10	1.2066	1.2438	15	0.9701	0.9205
WT No treatment - WT Only ATB	10	-2.6874	1.2563	15	-2.1391	0.3198
WT No treatment - HD Only ATB	10	-0.1162	1.3046	15	-0.0891	1.0000
WT No treatment - WT ATB_FMT	10	-1.4564	1.3420	15	-1.0852	0.8798
WT No treatment - HD ATB_FMT	10	-0.2690	1.3739	15	-0.1958	1.0000
HD No treatment - WT Only ATB	10	-3.8940	1.1951	15	-3.2584	**0.0491**
HD No treatment - HD Only ATB	10	-1.3228	1.1832	15	-1.1179	0.8665
HD No treatment - WT ATB_FMT	10	-2.6630	1.2868	15	-2.0695	0.3521
HD No treatment - HD ATB_FMT	10	-1.4756	1.2613	15	-1.1700	0.8440
WT Only ATB - HD Only ATB	10	2.5712	1.1699	15	2.1979	0.2941
WT Only ATB - WT ATB_FMT	10	1.2310	1.2597	15	0.9772	0.9183
WT Only ATB - HD ATB_FMT	10	2.4184	1.2875	15	1.8784	0.4504
HD Only ATB - WT ATB_FMT	10	-1.3402	1.3037	15	-1.0280	0.9012
HD Only ATB - HD ATB_FMT	10	-0.1528	1.2722	15	-0.1201	1.0000
WT ATB_FMT - HD ATB_FMT	10	1.1874	1.3256	15	0.8957	0.9417
WT No treatment - HD No treatment	11	1.7947	1.2673	15	1.4162	0.7175
WT No treatment - WT Only ATB	11	-2.5800	1.2891	15	-2.0014	0.3856
WT No treatment - HD Only ATB	11	0.5793	1.3626	15	0.4252	0.9979
WT No treatment - WT ATB_FMT	11	-1.5572	1.3762	15	-1.1315	0.8608
WT No treatment - HD ATB_FMT	11	0.2182	1.4311	15	0.1525	1.0000
HD No treatment - WT Only ATB	11	-4.3747	1.2483	15	-3.5046	**0.0310**
HD No treatment - HD Only ATB	11	-1.2154	1.2180	15	-0.9979	0.9116
HD No treatment - WT ATB_FMT	11	-3.3519	1.3414	15	-2.4988	0.1855
HD No treatment - HD ATB_FMT	11	-1.5765	1.2976	15	-1.2149	0.8232
WT Only ATB - HD Only ATB	11	3.1593	1.1948	15	2.6442	0.1461
WT Only ATB - WT ATB_FMT	11	1.0228	1.2949	15	0.7898	0.9650
WT Only ATB - HD ATB_FMT	11	2.7982	1.3426	15	2.0842	0.3451
HD Only ATB - WT ATB_FMT	11	-2.1366	1.3610	15	-1.5698	0.6285
HD Only ATB - HD ATB_FMT	11	-0.3611	1.3071	15	-0.2762	0.9997
WT ATB_FMT - HD ATB_FMT	11	1.7755	1.3476	15	1.3175	0.7716
WT No treatment - HD No treatment	12	2.3828	1.2968	15	1.8374	0.4730
WT No treatment - WT Only ATB	12	-2.4726	1.3301	15	-1.8590	0.4610
WT No treatment - HD Only ATB	12	1.2748	1.4338	15	0.8891	0.9434
WT No treatment - WT ATB_FMT	12	-1.6581	1.4190	15	-1.1685	0.8447
WT No treatment - HD ATB_FMT	12	0.7055	1.5015	15	0.4698	0.9966
HD No treatment - WT Only ATB	12	-4.8554	1.3136	15	-3.6964	**0.0216**
HD No treatment - HD Only ATB	12	-1.1080	1.2612	15	-0.8785	0.9460
HD No treatment - WT ATB_FMT	12	-4.0409	1.4086	15	-2.8688	0.0993
HD No treatment - HD ATB_FMT	12	-1.6773	1.3430	15	-1.2490	0.8066
WT Only ATB - HD Only ATB	12	3.7474	1.2261	15	3.0563	0.0711

Supplementary Table 6: Post-hoc results Post-hoc results for genotype*Treatment given Time in Fecal Output in female *(continued)*

contrast	Time	estimate	SE	df	t.ratio	p.value
WT Only ATB - WT ATB_FMT	12	0.8145	1.3388	15	0.6084	0.9887
WT Only ATB - HD ATB_FMT	12	3.1781	1.4104	15	2.2534	0.2711
HD Only ATB - WT ATB_FMT	12	-2.9329	1.4314	15	-2.0489	0.3620
HD Only ATB - HD ATB_FMT	12	-0.5693	1.3507	15	-0.4215	0.9979
WT ATB_FMT - HD ATB_FMT	12	2.3636	1.3755	15	1.7183	0.5411
WT No treatment - HD No treatment	13	2.9709	1.3321	15	2.2302	0.2805
WT No treatment - WT Only ATB	13	-2.3652	1.3785	15	-1.7158	0.5425
WT No treatment - HD Only ATB	13	1.9703	1.5162	15	1.2995	0.7810
WT No treatment - WT ATB_FMT	13	-1.7589	1.4697	15	-1.1968	0.8317
WT No treatment - HD ATB_FMT	13	1.1927	1.5832	15	0.7533	0.9713
HD No treatment - WT Only ATB	13	-5.3361	1.3892	15	-3.8412	**0.0164**
HD No treatment - HD Only ATB	13	-1.0006	1.3122	15	-0.7625	0.9698
HD No treatment - WT ATB_FMT	13	-4.7298	1.4865	15	-3.1818	0.0566
HD No treatment - HD ATB_FMT	13	-1.7782	1.3963	15	-1.2735	0.7944
WT Only ATB - HD Only ATB	13	4.3355	1.2634	15	3.4317	**0.0356**
WT Only ATB - WT ATB_FMT	13	0.6063	1.3906	15	0.4360	0.9976
WT Only ATB - HD ATB_FMT	13	3.5579	1.4890	15	2.3895	0.2205
HD Only ATB - WT ATB_FMT	13	-3.7293	1.5130	15	-2.4648	0.1959
HD Only ATB - HD ATB_FMT	13	-0.7776	1.4020	15	-0.5546	0.9926
WT ATB_FMT - HD ATB_FMT	13	2.9517	1.4088	15	2.0952	0.3399

Supplementary Table 7: Post-hoc results for genotype given Treatment in FITC

contrast	Treatment	estimate	SE	df	t.ratio	p.value
WT - HD	No treatment	-0.4804	0.2031	11	-2.3648	**0.0375**
WT - HD	Only ATB	-0.2980	0.1995	11	-1.4936	0.1634
WT - HD	ATB_FMT	-0.3774	0.2230	11	-1.6921	0.1187

Supplementary Table 8: Post-hoc results for Treatment given genotype in Propionate

contrast	genotype	estimate	SE	df	t.ratio	p.value
No treatment - Only ATB	WT	0.0569	5.0083	9	0.0114	0.9999
No treatment - ATB_FMT	WT	-10.8384	4.5975	9	-2.3575	0.0980
Only ATB - ATB_FMT	WT	-10.8953	5.4139	9	-2.0125	0.1648
No treatment - Only ATB	HD	-6.9653	4.5975	9	-1.5150	0.3293
No treatment - ATB_FMT	HD	-4.7370	4.5975	9	-1.0303	0.5775
Only ATB - ATB_FMT	HD	2.2283	4.1121	9	0.5419	0.8530

Supplementary Table 9: Post-hoc results for genotype given Treatment in IFNg

contrast	Treatment	estimate	SE	df	t.ratio	p.value
WT - HD	No treatment	0.0200	0.0076	12	2.6196	**0.0224**
WT - HD	Only ATB	0.0215	0.0076	12	2.8133	**0.0157**
WT - HD	ATB_FMT	0.0158	0.0076	12	2.0701	0.0607

Supplementary Table 10: Post-hoc results for genotype *Treatment in body weight in male

contrast	estimate	SE	df	t.ratio	p.value
WT No treatment - HD No treatment	5.5503	0.9342	71	5.9415	**0.0000**
WT No treatment - WT Only ATB	2.8106	1.0413	1297	2.6992	0.0760
WT No treatment - HD Only ATB	3.9178	0.9354	71	4.1886	**0.0011**
WT No treatment - WT ATB_FMT	0.5025	1.0205	71	0.4924	0.9963
WT No treatment - HD ATB_FMT	4.9871	1.0245	71	4.8679	**0.0001**
HD No treatment - WT Only ATB	-2.7396	0.9361	71	-2.9266	0.0503
HD No treatment - HD Only ATB	-1.6325	0.5282	1297	-3.0908	**0.0249**
HD No treatment - WT ATB_FMT	-5.0478	0.9141	71	-5.5224	**0.0000**
HD No treatment - HD ATB_FMT	-0.5632	0.9119	71	-0.6176	0.9894
WT Only ATB - HD Only ATB	1.1072	0.9336	71	1.1859	0.8421
WT Only ATB - WT ATB_FMT	-2.3082	1.0205	71	-2.2617	0.2236
WT Only ATB - HD ATB_FMT	2.1765	1.0245	71	2.1244	0.2869
HD Only ATB - WT ATB_FMT	-3.4153	0.9129	71	-3.7411	**0.0048**
HD Only ATB - HD ATB_FMT	1.0693	0.9113	71	1.1734	0.8480
WT ATB_FMT - HD ATB_FMT	4.4846	0.9988	71	4.4900	**0.0004**

Supplementary Table 11: Post-hoc results for genotype * Treatment in Weight Gain in male

contrast	estimate	SE	df	t.ratio	p.value
WT No treatment - HD No treatment	17.6839	3.5853	23	4.9324	**0.0007**
WT No treatment - WT Only ATB	7.2766	4.0791	682	1.7839	0.4769
WT No treatment - HD Only ATB	13.3880	3.5875	23	3.7319	**0.0123**
WT No treatment - WT ATB_FMT	2.1142	3.8698	23	0.5463	0.9935
WT No treatment - HD ATB_FMT	21.0392	3.8809	23	5.4212	**0.0002**
HD No treatment - WT Only ATB	-10.4073	3.5853	23	-2.9028	0.0759
HD No treatment - HD Only ATB	-4.2958	1.8509	682	-2.3209	0.1870
HD No treatment - WT ATB_FMT	-15.5697	3.3452	23	-4.6544	**0.0014**
HD No treatment - HD ATB_FMT	3.3553	3.3580	23	0.9992	0.9135
WT Only ATB - HD Only ATB	6.1114	3.5875	23	1.7035	0.5432
WT Only ATB - WT ATB_FMT	-5.1624	3.8698	23	-1.3340	0.7637
WT Only ATB - HD ATB_FMT	13.7626	3.8809	23	3.5463	**0.0188**
HD Only ATB - WT ATB_FMT	-11.2738	3.3476	23	-3.3678	**0.0281**
HD Only ATB - HD ATB_FMT	7.6512	3.3604	23	2.2769	0.2434
WT ATB_FMT - HD ATB_FMT	18.9250	3.6602	23	5.1704	**0.0004**

Supplementary Table 12: Post-hoc results for genotype * Treatment in Fecal Water Content in male

contrast	estimate	SE	df	t.ratio	p.value
WT No treatment - HD No treatment	15.6830	8.5175	23	1.8413	0.4608
WT No treatment - WT Only ATB	0.6866	9.7349	677	0.0705	1.0000
WT No treatment - HD Only ATB	-9.6736	8.5237	23	-1.1349	0.8618
WT No treatment - WT ATB_FMT	4.6863	9.2371	23	0.5073	0.9954
WT No treatment - HD ATB_FMT	-1.0366	9.2566	23	-0.1120	1.0000
HD No treatment - WT Only ATB	-14.9964	8.5175	23	-1.7607	0.5086
HD No treatment - HD Only ATB	-25.3566	4.1105	677	-6.1687	**0.0000**
HD No treatment - WT ATB_FMT	-10.9967	7.9437	23	-1.3843	0.7356
HD No treatment - HD ATB_FMT	-16.7196	7.9664	23	-2.0988	0.3224

Supplementary Table 12: Post-hoc results for genotype * Treatment in Fecal Water Content in male *(continued)*

contrast	estimate	SE	df	t.ratio	p.value
WT Only ATB - HD Only ATB	-10.3603	8.5237	23	-1.2155	0.8249
WT Only ATB - WT ATB_FMT	3.9997	9.2371	23	0.4330	0.9978
WT Only ATB - HD ATB_FMT	-1.7233	9.2566	23	-0.1862	1.0000
HD Only ATB - WT ATB_FMT	14.3599	7.9504	23	1.8062	0.4814
HD Only ATB - HD ATB_FMT	8.6370	7.9730	23	1.0833	0.8830
WT ATB_FMT - HD ATB_FMT	-5.7229	8.7315	23	-0.6554	0.9851

Supplementary Table 13: Post-hoc results for genotype * Treatment in Food Intake in male

contrast	estimate	SE	df	t.ratio	p.value
WT No treatment - HD No treatment	-0.0973	0.0345	22	-2.8239	0.0906
WT No treatment - WT Only ATB	-0.1906	0.0366	292	-5.2061	**0.0000**
WT No treatment - HD Only ATB	-0.0659	0.0345	22	-1.9130	0.4208
WT No treatment - WT ATB_FMT	-0.1767	0.0347	22	-5.0869	**0.0005**
WT No treatment - HD ATB_FMT	-0.0519	0.0366	22	-1.4181	0.7162
HD No treatment - WT Only ATB	-0.0933	0.0345	22	-2.7089	0.1134
HD No treatment - HD Only ATB	0.0314	0.0304	292	1.0328	0.9066
HD No treatment - WT ATB_FMT	-0.0794	0.0324	22	-2.4472	0.1835
HD No treatment - HD ATB_FMT	0.0454	0.0345	22	1.3168	0.7729
WT Only ATB - HD Only ATB	0.1247	0.0345	22	3.6199	**0.0166**
WT Only ATB - WT ATB_FMT	0.0139	0.0347	22	0.4008	0.9985
WT Only ATB - HD ATB_FMT	0.1387	0.0366	22	3.7879	**0.0113**
HD Only ATB - WT ATB_FMT	-0.1108	0.0324	22	-3.4144	**0.0262**
HD Only ATB - HD ATB_FMT	0.0140	0.0345	22	0.4058	0.9984
WT ATB_FMT - HD ATB_FMT	0.1248	0.0347	22	3.5921	**0.0177**

Supplementary Table 14: Post-hoc results for Treatment given genotype in Water Intake in male

contrast	genotype	estimate	SE	df	t.ratio	p.value
No treatment - Only ATB	WT	-0.3721	0.1511	264	-2.4622	**0.0383**
No treatment - ATB_FMT	WT	-0.2737	0.1434	22	-1.9092	0.1598
Only ATB - ATB_FMT	WT	0.0984	0.1434	22	0.6862	0.7739
No treatment - Only ATB	HD	-0.2293	0.0974	264	-2.3526	0.0505
No treatment - ATB_FMT	HD	-0.3113	0.1372	22	-2.2691	0.0817
Only ATB - ATB_FMT	HD	-0.0820	0.1372	22	-0.5974	0.8229

Supplementary Table 15: Post-hoc results for genotype given Treatment*Arm in Ymaze in male

contrast	Treatment	Arm	estimate	SE	df	t.ratio	p.value
WT - HD	No treatment	Novel	17.1598	8.2060	39	2.0911	**0.0431**
WT - HD	Only ATB	Novel	2.8606	7.7449	39	0.3694	0.7139
WT - HD	ATB_FMT	Novel	14.4345	7.5293	39	1.9171	0.0626
WT - HD	No treatment	Familiar	16.3095	8.2060	39	1.9875	0.0539
WT - HD	Only ATB	Familiar	2.0103	7.7449	39	0.2596	0.7966

Supplementary Table 15: Post-hoc results for genotype given Treatment*Arm in Ymaze in male *(continued)*

contrast	Treatment	Arm	estimate	SE	df	t.ratio	p.value
WT - HD	ATB_FMT	Familiar	13.5842	7.5293	39	1.8042	0.0789

Supplementary Table 16: Post-hoc results for for genotype given Treatment in Brain weight in male

contrast	Treatment	estimate	SE	df	t.ratio	p.value
WT - HD	No treatment	0.0700	0.0213	23	3.2864	**0.0032**
WT - HD	Only ATB	0.0510	0.0202	23	2.5239	**0.0190**
WT - HD	ATB_FMT	0.0644	0.0201	23	3.2091	**0.0039**

Supplementary Table 17: Post-hoc results for genotype given Treatment in Propel Brake Ratio in male

contrast	Treatment	estimate	SE	df	t.ratio	p.value
WT - HD	No treatment	-1.4822	0.4934	45	-3.0043	**0.0043**
WT - HD	Only ATB	0.3485	0.4786	42	0.7281	0.4706
WT - HD	ATB_FMT	-0.9988	0.4918	45	-2.0310	**0.0482**

Supplementary Table 18: Post-hoc results for genotype given Treatment in FITC in male

contrast	Treatment	estimate	SE	df	t.ratio	p.value
WT - HD	No treatment	-0.5967	0.2329	12	-2.5616	**0.0249**
WT - HD	Only ATB	-0.2904	0.2277	12	-1.2756	0.2262
WT - HD	ATB_FMT	-0.8545	0.2211	12	-3.8653	**0.0022**

Supplementary Table 19: Post-hoc results for Treatment given genotype in Cecum Weight in male

contrast	genotype	estimate	SE	df	t.ratio	p.value
No treatment - Only ATB	WT	0.0300	0.0350	16	0.8560	0.6747
No treatment - ATB_FMT	WT	0.0850	0.0379	16	2.2455	0.0936
Only ATB - ATB_FMT	WT	0.0550	0.0379	16	1.4530	0.3388
No treatment - Only ATB	HD	-0.0193	0.0363	16	-0.5317	0.8571
No treatment - ATB_FMT	HD	0.0477	0.0410	16	1.1626	0.4914
Only ATB - ATB_FMT	HD	0.0670	0.0400	16	1.6768	0.2442

Supplementary Table 20: Post-hoc results for genotype given Treatment in Colon length in male

contrast	Treatment	estimate	SE	df	t.ratio	p.value
WT - HD	No treatment	1.2244	0.4248	23	2.8821	**0.0084**
WT - HD	Only ATB	0.4483	0.3918	22	1.1443	0.2648
WT - HD	ATB_FMT	0.8804	0.3882	23	2.2675	**0.0331**

Supplementary Table 21: Post-hoc results for genotype Treatment in Acetate in male

contrast	estimate	SE	df	t.ratio	p.value
WT No treatment - HD No treatment	9.5017	18.6410	12	0.5097	0.9948
WT No treatment - WT Only ATB	10.3542	16.6730	12	0.6210	0.9872
WT No treatment - HD Only ATB	-37.8161	17.4868	12	-2.1625	0.3206
WT No treatment - WT ATB_FMT	20.1300	16.6730	12	1.2073	0.8254
WT No treatment - HD ATB_FMT	-10.0521	17.4868	12	-0.5748	0.9909
HD No treatment - WT Only ATB	0.8525	18.6410	12	0.0457	1.0000
HD No treatment - HD Only ATB	-47.3178	19.3723	12	-2.4425	0.2161
HD No treatment - WT ATB_FMT	10.6283	18.6410	12	0.5702	0.9913
HD No treatment - HD ATB_FMT	-19.5538	19.3723	12	-1.0094	0.9061
WT Only ATB - HD Only ATB	-48.1703	17.4868	12	-2.7547	0.1342
WT Only ATB - WT ATB_FMT	9.7758	16.6730	12	0.5863	0.9901
WT Only ATB - HD ATB_FMT	-20.4063	17.4868	12	-1.1670	0.8439
HD Only ATB - WT ATB_FMT	57.9461	17.4868	12	3.3137	0.0539
HD Only ATB - HD ATB_FMT	27.7640	18.2644	12	1.5201	0.6593
WT ATB_FMT - HD ATB_FMT	-30.1821	17.4868	12	-1.7260	0.5414

Supplementary Table 22: Post-hoc results for Treatment given genotype in IL17E in male

contrast	genotype	estimate	SE	df	t.ratio	p.value
No treatment - Only ATB	WT	0.0321	0.1801	14	0.1782	0.9827
No treatment - ATB_FMT	WT	-0.4044	0.1819	14	-2.2229	0.1018
Only ATB - ATB_FMT	WT	-0.4365	0.1877	14	-2.3252	0.0850
No treatment - Only ATB	HD	0.2024	0.2292	14	0.8833	0.6592
No treatment - ATB_FMT	HD	0.1352	0.2339	14	0.5783	0.8337
Only ATB - ATB_FMT	HD	-0.0672	0.1952	14	-0.3442	0.9371

Supplementary Table 23: Linear mixed model results for Propionate, Isobutyrate and Methylbutyrate (Week 14) for females

	Dependent variable:		
	Propionate-14	Isobutyrate-14	Methylbutyrate-14
	(1)	(2)	(3)
genotypeHD	−0.7309	−0.7858	0.4986
	p = 0.9007	p = 0.3314	p = 0.1249
TreatmentOnly ATB	0.1275	−0.0158	0.0564
	p = 0.9821	p = 0.9834	p = 0.8497
TreatmentATB_FMT	−2.0237	−0.3104	−0.0682
	p = 0.7257	p = 0.6963	p = 0.8252
genotypeHD:TreatmentOnly ATB	0.2815	0.4770	−0.5904
	p = 0.9730	p = 0.6717	p = 0.1924
genotypeHD:TreatmentATB_FMT	−2.1452	1.5722	−0.4113
	p = 0.7942	p = 0.1866	p = 0.3633
Constant	9.6166*	2.9692***	0.9686***
	p = 0.0327	p = 0.0001	p = 0.0004
Observations	28	32	32
Log Likelihood	−76.5134	−48.3574	−21.2659
Akaike Inf. Crit.	169.0268	112.7149	58.5317
Bayesian Inf. Crit.	177.7551	122.7796	68.5965
Note:	+ p<0.1; * p<0.05; ** p<0.01; *** p<0.001		

Supplementary Table 24: Linear mixed model results for Isobutyrate and Methylbutyrate (Week 20) for females

	Dependent variable:	
	Isobutyrate-20	Methylbutyrate-20
	(1)	(2)
genotypeHD	0.3708	0.1820
	p = 0.6891	p = 0.5637
TreatmentOnly ATB	0.4860	0.4082
	p = 0.6316	p = 0.2414
TreatmentATB_FMT	1.0963	0.4162
	p = 0.2528	p = 0.2038
genotypeHD:TreatmentOnly ATB	0.3220	−0.0468
	p = 0.8139	p = 0.9187
genotypeHD:TreatmentATB_FMT	0.0403	−0.1764
	p = 0.9754	p = 0.6909
Constant	2.3977***	0.7544**
	p = 0.0009	p = 0.0016
Observations	29	29
Log Likelihood	−44.3597	−16.9530
Akaike Inf. Crit.	104.7194	49.9061
Bayesian Inf. Crit.	113.8034	58.9900
Note:	+ p<0.1; * p<0.05; ** p<0.01; *** p<0.001	

Supplementary Table 25: Linear mixed model results for Propionate, Isobutyrate and Methylbutyrate (Week 14) for males

	Dependent variable:		
	Propionate-14	Isobutyrate-14	Methylbutyrate-14
	(1)	(2)	(3)
genotypeHD	0.9088	0.5904	0.1806
	p = 0.5602	p = 0.2188	p = 0.2247
TreatmentOnly ATB	1.7019	0.3049	−0.1399
	p = 0.3241	p = 0.5616	p = 0.3874
TreatmentATB_FMT	−1.5247	−0.3897	−0.2389
	p = 0.4770	p = 0.5441	p = 0.2377
Constant	2.9463*	2.5912***	0.7841***
	p = 0.0469	p = 0.00002	p = 0.00002
Observations	36	35	36
Log Likelihood	−97.9223	−55.1535	−20.7752
Akaike Inf. Crit.	207.8446	122.3070	53.5503
Bayesian Inf. Crit.	216.6390	130.9109	62.3447
Note:	+ p<0.1; * p<0.05; ** p<0.01; *** p<0.001		

Supplementary Table 26: Linear mixed model results for Isobutyrate and Methylbutyrate (Week 20) for males

	Dependent variable:	
	Isobutyrate-20	Methylbutyrate-20
	(1)	(2)
genotypeHD	0.0254	0.2137
	p = 0.9763	p = 0.7970
TreatmentOnly ATB	0.7819	1.4797+
	p = 0.3360	p = 0.0644
TreatmentATB_FMT	1.1532	−0.1066
	p = 0.1651	p = 0.8859
genotypeHD:TreatmentOnly ATB	1.4744	−0.9962
	p = 0.2282	p = 0.3887
genotypeHD:TreatmentATB_FMT	0.6947	0.3270
	p = 0.5607	p = 0.7741
Constant	2.6521***	0.6934
	p = 0.0003	p = 0.1986
Observations	32	32
Log Likelihood	−44.8358	−47.8591
Akaike Inf. Crit.	105.6716	111.7182
Bayesian Inf. Crit.	115.7364	121.7829
Note:	+ p<0.1; * p<0.05; ** p<0.01; *** p<0.001	

Supplementary Table 27: Linear mixed model results for IL21, IL17A and IL17E (Week 14) for females

	Dependent variable:		
	IL21-14	IL17A-14	IL17E-14
	(1)	(2)	(3)
genotypeHD	-0.1450^{+}	-0.0103	0.2609
	p = 0.0850	p = 0.8914	p = 0.3931
TreatmentOnly ATB	-0.0533	0.0665	0.3395
	p = 0.5576	p = 0.3849	p = 0.2725
TreatmentATB_FMT	-0.0493	0.0182	0.5259
	p = 0.6213	p = 0.8094	p = 0.1023
genotypeHD:TreatmentOnly ATB		-0.0337	0.0339
		p = 0.7521	p = 0.9413
genotypeHD:TreatmentATB_FMT		-0.0763	-0.7119
		p = 0.4781	p = 0.1278
Constant	0.5979^{***}	0.1702^{**}	0.6938^{**}
	p = 0.000003	p = 0.0044	p = 0.0061
Observations	26	36	30
Log Likelihood	1.0641	14.1085	-19.8117
Akaike Inf. Crit.	9.8717	-12.2171	55.6234
Bayesian Inf. Crit.	16.4180	-1.0075	65.0479
Note:	+ p<0.1; * p<0.05; ** p<0.01; *** p<0.001		

Supplementary Table 28: Linear mixed model results for IL1b, IL22 and TNFa (Week 14) for females

	Dependent variable:		
	IL1b-14	IL22-14	TNFa-14
	(1)	(2)	(3)
genotypeHD	0.8150	0.4568	0.0258
	p = 0.1036	p = 0.3588	p = 0.9825
TreatmentOnly ATB	-0.0696	-0.0976	-0.0080
	p = 0.8830	p = 0.8419	p = 0.9944
TreatmentATB_FMT	0.0325	0.7720	1.6155
	p = 0.9451	p = 0.1327	p = 0.1686
genotypeHD:TreatmentOnly ATB	-0.6118	-0.2245	0.3394
	p = 0.3681	p = 0.7459	p = 0.8378
genotypeHD:TreatmentATB_FMT	-0.1118	-1.1779	-1.5066
	p = 0.8672	p = 0.1074	p = 0.3624
Constant	0.6141^{+}	0.6753^{+}	0.8728
	p = 0.0768	p = 0.0614	p = 0.2794
Observations	36	36	34
Log Likelihood	-39.1337	-42.3066	-60.4890
Akaike Inf. Crit.	94.2674	100.6132	136.9779
Bayesian Inf. Crit.	105.4769	111.8228	147.6356
Note:	+ p<0.1; * p<0.05; ** p<0.01; *** p<0.001		

Supplementary Table 29: Linear mixed model results for IL17A, IL1b and IL21 (Week 20) for females

	Dependent variable:		
	IL17A-20	IL1b-20	IL21-20
	(1)	(2)	(3)
genotypeHD	-0.1527^{+}	0.4596	-0.0047
	p = 0.0744	p = 0.1730	p = 0.9510
TreatmentOnly ATB	-0.0509	0.2706	0.1423
	p = 0.5686	p = 0.4590	p = 0.1190
TreatmentATB_FMT	-0.1382	0.2454	-0.0622
	p = 0.1389	p = 0.5009	p = 0.4752
genotypeHD:TreatmentOnly ATB	0.0785	-0.3485	-0.2108^{+}
	p = 0.5134	p = 0.4767	p = 0.0888
genotypeHD:TreatmentATB_FMT	0.2035^{+}	-0.1846	0.2091^{+}
	p = 0.0990	p = 0.6958	p = 0.0833
Constant	0.2588^{***}	0.4852^{*}	0.3807^{***}
	p = 0.0002	p = 0.0440	p = 0.000002
Observations	34	34	34
Log Likelihood	20.6591	-27.8835	12.2008
Akaike Inf. Crit.	-25.3181	71.7670	-8.4017
Bayesian Inf. Crit.	-14.6605	82.4246	2.2559
Note:	+ p<0.1; * p<0.05; ** p<0.01; *** p<0.001		

Supplementary Table 30: Linear mixed model results for IL22, IL6 and TNFa (Week 20) for females

	Dependent variable:		
	IL22-20	IL6-20	TNFa-20
	(1)	(2)	(3)
genotypeHD	0.4040	-0.4964	-0.4820
	p = 0.6271	p = 0.1477	p = 0.1298
TreatmentOnly ATB	0.1274	0.1559	-0.3684
	p = 0.8905	p = 0.6703	p = 0.2855
TreatmentATB_FMT	-0.0860	-0.2278	-0.3580
	p = 0.9259	p = 0.5358	p = 0.2988
genotypeHD:TreatmentOnly ATB	-0.6839	-0.0815	0.4548
	p = 0.5841	p = 0.8679	p = 0.3216
genotypeHD:TreatmentATB_FMT	0.6249	0.6418	0.7472
	p = 0.6065	p = 0.1947	p = 0.1072
Constant	0.8332	1.1527^{***}	1.1667^{***}
	p = 0.1632	p = 0.0001	p = 0.00005
Observations	34	34	33
Log Likelihood	-52.5298	-21.1180	-11.3178
Akaike Inf. Crit.	121.0595	58.2361	38.6356
Bayesian Inf. Crit.	131.7172	68.8937	49.0023
Note:	+ p<0.1; * p<0.05; ** p<0.01; *** p<0.001		

Supplementary Table 31: Linear mixed model results for IL21, IL17A and IL17E (Week 14) for males

	Dependent variable:		
	IL21-14	IL17A-14	IL17E-14
	(1)	(2)	(3)
genotypeHD	−0.0558	0.0589	0.4301
	p = 0.4973	p = 0.6692	p = 0.1134
TreatmentOnly ATB	−0.0518	−0.1186	0.1212
	p = 0.5905	p = 0.4588	p = 0.6958
TreatmentATB_FMT	0.1637	−0.0928	0.3462
	p = 0.1212	p = 0.5949	p = 0.2649
Constant	0.3083***	0.2550+	0.5904*
	p = 0.0009	p = 0.0615	p = 0.0206
Observations	31	31	26
Log Likelihood	3.9388	−12.6930	−21.9144
Akaike Inf. Crit.	4.1224	37.3860	55.8288
Bayesian Inf. Crit.	11.8975	45.1610	62.3750
Note:	+ p<0.1; * p<0.05; ** p<0.01; *** p<0.001		

Supplementary Table 32: Linear mixed model results for IL1b, IL22 and TNFa (Week 14) for males

	Dependent variable:		
	IL1b-14	IL22-14	TNFa-14
	(1)	(2)	(3)
genotypeHD	−0.0718	−0.0097	0.3622
	p = 0.7599	p = 0.9651	p = 0.9964
TreatmentOnly ATB	0.2181	−0.0400	−0.0461
	p = 0.3593	p = 0.8577	p = 0.9996
TreatmentATB_FMT	0.1976	0.1203	0.3905
	p = 0.4268	p = 0.6081	p = 0.9964
genotypeHD:TreatmentOnly ATB	−0.1764	−0.1169	0.2442
	p = 0.5900	p = 0.7061	p = 0.9983
genotypeHD:TreatmentATB_FMT	0.0438	−0.0483	150.3674
	p = 0.8999	p = 0.8875	p = 0.2201
Constant	0.3428+	0.5447**	0.8442
	p = 0.0529	p = 0.0032	p = 0.9876
Observations	35	34	33
Log Likelihood	−19.8067	−17.7358	−174.8050
Akaike Inf. Crit.	55.6134	51.4717	365.6099
Bayesian Inf. Crit.	66.5518	62.1293	375.9766
Note:	+ p<0.1; * p<0.05; ** p<0.01; *** p<0.001		

Supplementary Table 33: Linear mixed model results for IL17A, IL1b and IL21 (Week 20) for males

	Dependent variable:		
	IL17A-20	IL1b-20	IL21-20
	(1)	(2)	(3)
genotypeHD	−0.0107	0.1719	−0.2490
	p = 0.8701	p = 0.7236	p = 0.1524
TreatmentOnly ATB	−0.0146	0.3688	−0.1318
	p = 0.8388	p = 0.4511	p = 0.3863
TreatmentATB_FMT	0.0685	−0.1591	0.0579
	p = 0.3454	p = 0.7535	p = 0.7138
genotypeHD:TreatmentOnly ATB	0.0787	0.3263	0.1306
	p = 0.4319	p = 0.6426	p = 0.5842
genotypeHD:TreatmentATB_FMT	0.0824	0.1651	0.1002
	p = 0.4164	p = 0.8172	p = 0.6710
Constant	0.1448**	0.6860+	0.7783***
	p = 0.0062	p = 0.0581	p = 0.000003
Observations	35	39	30
Log Likelihood	17.3611	−47.8369	−6.1180
Akaike Inf. Crit.	−18.7222	111.6737	28.2359
Bayesian Inf. Crit.	−7.7838	123.6458	37.6604
Note:	+ p<0.1; * p<0.05; ** p<0.01; *** p<0.001		

Supplementary Table 34: Linear mixed model results for IL22, IL6 and TNFa (Week 20) for males

	Dependent variable:		
	IL22-20	IL6-20	TNFa-20
	(1)	(2)	(3)
genotypeHD	−0.0978	−0.2050	−0.9973
	p = 0.7838	p = 0.7827	p = 0.5521
TreatmentOnly ATB	0.1930	0.0898	−0.7883
	p = 0.5895	p = 0.9038	p = 0.6377
TreatmentATB_FMT	−0.1003	1.0858	−0.8374
	p = 0.7868	p = 0.1762	p = 0.6308
genotypeHD:TreatmentOnly ATB	0.5237	0.3921	0.6315
	p = 0.3159	p = 0.7163	p = 0.7933
genotypeHD:TreatmentATB_FMT	0.3911	−0.3485	4.3945+
	p = 0.4593	p = 0.7500	p = 0.0956
Constant	1.1129***	0.8100	1.7749
	p = 0.0004	p = 0.1356	p = 0.1458
Observations	39	39	38
Log Likelihood	−38.4919	−59.1479	−86.8274
Akaike Inf. Crit.	92.9839	134.2957	189.6548
Bayesian Inf. Crit.	104.9559	146.2678	201.3807
Note:	+ p<0.1; * p<0.05; ** p<0.01; *** p<0.001		
