# Supplementary material for: Faecal microbiota transplant ameliorates gut dysbiosis and cognitive deficits in Huntington’s disease mice
Source: Brain Commun. 2022 Aug 12;4(4):fcac205. doi: 10.1093/braincomms/fcac205 (PMC9400176; doi:10.1093/braincomms/fcac205)
Supplement: fcac205_Supplementary_Data [file fcac205_supplementary_data.zip › Manuscript_revision-1.pdf]

OXFORD UNIVERSITY PRESS | Brain Communications

Fecal microbiota transplant ameliorates gut dysbiosis and cognitive deficits in Huntington's disease mice

Journal:	*Brain Communications*
Manuscript ID	BRAINCOM-2022-109.R1
Manuscript Type:	Original Article
Date Submitted by the Author:	21-May-2022

Complete List of Authors:	Gubert, Carolina; The Florey Institute of Neuroscience and Mental Health<br>Choo, Jocelyn ; SAHMRI<br>Love, Chloe; The Florey Institute of Neuroscience and Mental Health<br>Kodikara, Saritha; The University of Melbourne<br>Masson, Bethany; The Florey Institute of Neuroscience and Mental Health<br>Liew, Jamie ; The Florey Institute of Neuroscience and Mental Health<br>Wang, Yiwen; The Florey Institute of Neuroscience and Mental Health<br>Kong, Geraldine; The Peter Doherty Institute for Infection and Immunity<br>Narayana, Vinod; The University of Melbourne, Department of Biochemistry and Molecular Biology<br>Renoir, Thibault; The Florey Institute of Neuroscience and Mental Health<br>Lê Cao, Kim-Anh; The University of Melbourne<br>Rogers, Geraint ; The Florey Institute of Neuroscience and Mental Health<br>Hannan, Anthony; The Florey Institute of Neuroscience and Mental Health; The University of Melbourne

Keywords:	dementia, gut microbiome, microbiota-gut-brain axis, neurodegenerative disorder, microbiota, tandem repeat disorder

SCHOLARONE™ Manuscripts

Fecal microbiota transplant ameliorates gut dysbiosis and cognitive deficits in Huntington's disease mice

Carolina Gubert[1,], Jocelyn M. Choo[2,3], Chloe J. Love[1], Saritha Kodikara[4], Bethany A. Masson[1], Jamie J. M. Liew[1], Yiwen Wang[4], Geraldine Kong[1], Vinod K. Narayana[5], Thibault Renoir[1], Kim-Anh Lê Cao[4], Geraint B. Rogers[2,3] and Anthony J. Hannan[1,6,]

Abstract

Huntington's disease (HD) is a neurodegenerative disorder involving psychiatric, cognitive and motor symptoms. HD is caused by a tandem-repeat expansion in the huntingtin gene, which is widely expressed throughout the brain and body, including the gastrointestinal system. There are currently no effective disease-modifying treatments available for this fatal disorder. Despite recent evidence of gut microbiome disruption in preclinical and clinical HD, its potential as a target for therapeutic interventions has not been explored. The microbiota-gut-brain axis provides a potential pathway via which changes in the gut could modulate brain function, including cognition. We now show that fecal microbiota transplant (FMT) from wild-type (WT) into HD mice positively modulates cognitive outcomes, particularly in females. In HD males, we revealed an inefficiency of FMT engraftment, which is potentially due to the more pronounced changes of the structure, composition and instability of the gut microbial community, and the imbalance in acetate and gut immune profiles found in these mice. This study demonstrates a role for gut microbiome modulation in ameliorating cognitive deficits modelling dementia in HD. Our findings pave the way for the development of future therapeutic approaches, including FMT and other forms of gut microbiome modulation, as potential clinical interventions for HD.

For Review Only

Author affiliations:

[1]Florey Institute of Neuroscience and Mental Health, Melbourne Brain Centre, University of Melbourne, Parkville, Victoria, Australia
[2]Microbiome and Host Health, South Australian Health and Medical Research Institute, Adelaide, SA, 5001, Australia.
[3]Infection and Immunity, Flinders Health and Medical Research Institute, College of Medicine and Public Health, Flinders University, Bedford Park, SA, 5042, Australia.
[4]Melbourne Integrative Genomics, School of Mathematics and Statistics, University of Melbourne, Parkville, Victoria, Australia
[5]Bio21 Institute and Department of Biochemistry and Molecular Biology, University of Melbourne, Parkville, Victoria, Australia
[6]Department of Anatomy and Neuroscience, University of Melbourne, Parkville, Victoria, Australia
[5]Bio21 Institute and Department of Biochemistry and Molecular Biology, University of Melbourne, Parkville, Victoria, Australia
[6]Department of Anatomy and Physiology, University of Melbourne, Parkville, Victoria, Australia

Correspondence to: Prof. A.J. Hannan or Dr C. Gubert,

Florey Institute of Neuroscience and Mental Health, University of Melbourne, Melbourne Brain Centre, Parkville VIC 3010, Australia

E-mail: anthony.hannan@florey.edu.au, carolina.gubert@florey.edu.au

For Review Only

Running title: Fecal matter transplant ameliorates dementia in HD model

Keywords: dementia, gut microbiome, Huntington's disease, microbiota-gut-brain axis, neurodegenerative disorder

Abbreviations: ATB = antibiotic; BCFA = branched-chain fatty acid; CFC = contextual fear conditioning; CS = conditioned stimulus; FMT = fecal matter transplant; FITC = fluorescein isothiocyanate; GI = gastrointestinal; HD = Huntington's disease; HTT = huntingtin; LMM = linear mixed models; SCFA = short-chain fatty acid; US = unconditioned stimulus; WT = wild-type.

Introduction

Huntington's disease (HD) is a fatal neurodegenerative disorder for which there are currently no effective disease-modifying treatments[1]. HD has a complex core symptomology, including motor deficits, cognitive and psychiatric symptoms, with devastating impacts on HD patients and their families[2]. HD is caused by expansions of trinucleotide (CAG) tandem DNA repeats in the huntingtin (*HTT*) gene[3]. The mutated huntingtin protein, containing an expanded polyglutamine tract, is expressed ubiquitously throughout the body, affecting both the brain and periphery[4]. Gastrointestinal (GI) dysfunctions are serious complications of HD, and can include the presence of constipation, weight loss and nutrient deficiency, as well as impairment in gut structure, permeability and motility[5–8]. R6/1 transgenic HD mice express the mutant human huntingtin transgene and provide an excellent preclinical model, exhibiting progressive cognitive, behavioral, cellular and molecular deficits closely modeling clinical HD[9–11], including the onset of gut dysfunction at early stages of the disease with the potential to worsen with disease progression[12].

The community of microorganisms that colonise the gut, and their activity (gut microbiome), have been shown to influence brain function[13]. Disruption of this microbial ecosystem occurs in various neurodegenerative conditions, including Parkinson's and Alzheimer's disease[14,15]. More recently, gut microbiome disruption has been consistently shown in both preclinical and clinical HD[8,16–19], even before the onset of motor symptoms[16,17]. Importantly, in HD gene expansion carriers (including symptomatic individuals), alterations to the gut microbiota were demonstrated to be associated with inflammatory status[19], cognitive performance and clinical outcomes[18].

Fecal microbiota transplant (FMT) from healthy donors has been shown to be an effective approach for the treatment of some diseases that affect the GI tract and that have gut microbial disruption as a pathological feature, such as *Clostridium difficile* infection[19] and

For Review Only

active ulcerative colitis[20], potentially by restoring the GI tract with protective effects of the commensal microbiota. Recent studies have also investigated FMT as an approach for brain disorders including autism spectrum disorder[21] and Alzheimer's disease[22].

We hypothesize that interventions that ameliorate gut microbiome disruption will in turn be therapeutic in HD. In this study, we investigated whether FMT from wild-type (WT) into HD mice will be therapeutic, an approach that has shown promise for some other brain disorders, but has never been tested in HD. We assessed FMT impacts on the onset and progression of various aspects of the disease in the R6/1 transgenic mouse model of HD.

Materials and methods

Subject Details

Male R6/1 hemizygous mice (originally from Jackson Laboratories, USA) after back-crossing onto the CBA background for more than 10 generations (generating the CBAxC57Bl/6 strain background) were crossed with female CBAxC57Bl/6 mice to generate male and female wild-type (WT) and R6/1 (HD) littermates. Males and females were bred and housed separately, therefore sex was not added as a factor of the study. Genomic DNA from a tail biopsy was used to characterise genotypes. Due to their coprophagic nature, mice were housed according to genotype, sex and treatment to avoid any sharing of microbiota as ingestion of cage-mate feces can modulate the gut microbiota (2-5 mice per cage). Mice were housed in open-top cages (34 × 16 × 16 cm) with basic sterilized wood shavings and facial tissues for bedding and nesting materials. Cages are sterilised through a washer at 82℃ before use.

All mice had *ad libitum* access to sterilized food and filtered water (through a 0.5µm filter) and were housed in a room with a 12:12 h light/dark cycle, controlled for temperature (22 °C) and humidity (45%). Cages were changed and body-weight assessment was performed

For Review Only

weekly. All experiments and procedures were approved by The Florey Institute of Neuroscience and Mental Health Ethics Committee and were performed following the research guidelines and regulations of the National Health and Medical Research Council.

Preparation of donor cecal content for fecal microbiota transplantation

Sex and age matched WT littermate mice, naive to behavioural experiments and grouped housed (with their donor-group mates) were used as fecal donors. Donors were culled via cervical dislocation and caeca were harvested immediately and transferred to 15% glycerol-phosphate buffered saline (PBS). The subsequent processing was performed under anaerobic conditions (10% CO2, 10% H2, 80% N2), following a previously published protocol with minor modifications (Choo 2021). Cecal contents were removed from cecal tissue, weighed, resuspended in 4x (w/v) anaerobic PBS, and homogenised by vortexing. The resulting suspension was passed through a Falcon 100 µM nylon cell filter (Thermofisher Scientific, Waltham, USA) to obtain the non-fibrous content. The supernatant from all collected caeca was pooled, mixed with an equal volume of 30% anaerobic glycerol-PBS, and stored in 3 Hungate tubes (one tube per day of the gavaging to avoid freeze-thaw cycles) at -80 C until required (the solution was prepared in the same week of the gavaging, therefore not being stored for more than 7 days). Prior to oral gavage, pooled cecal supernatant was diluted with 2x volume of anaerobic PBS (pH 7.2) resulting in a solution of 5mg/mL of cecal content.

For Review Only

Experimental Models

To prepare the host for the subsequent FMT[32], ATB were used to induce a disruption of the gut microbiota[31]. Mice were randomly assigned into either control (vehicle/vehicle), ATB-only

(ATB/vehicle) or ATB/FMT groups (Fig. 1A). The ATB group had *ad libitum* access to a non-absorbable antibiotics (ATB) cocktail of ertapenem sodium, vancomycin hydrochloride (both from Glentham Life Sciences) and neomycin sulphate (Sigma-Aldrich) in sterile water at a ratio of 1 mg ml−1, for 7 days (at week 8 of age), following a previous published protocol[31]. The vehicle of ATB intervention was drinking water. Non-absorbable antibiotics have a relatively small degree of systemic absorption, decreasing possible off target effects. The FMT intervention consisted of oral administration of 150µl of the prepared 5g/mL of donor caeca content solution, for 3 days with 2 days of spacing (at 9 weeks of age). The vehicle control for the FMT intervention was oral administration of 7.5% glycerol-PBS. Plastic needles were used to minimise the effect of the gavage on the mice. The control group received neither ATB nor FMT, only the respective vehicle solution (drinking water and 7.5% glycerol-PBS).

Food and Water Intake

Food and water intake was assessed from 6 to 20 weeks of age (**Fig. 1A**). To account for individual weight variability, intake was normalised to body weight and results represent g of food per g of body weight and ml of water per g of body weight.

Motor Testing

Rotarod

Rotarod testing was used as an indicator of motor co-ordination[46]. The Rotarod (Ugo Basile, Varese, Italy) consists of a motorised rotating cylinder that is divided into five compartments. One mouse is placed into each compartment and the rotating cylinder accelerates at a constant speed of four rpm before the cylinder gradually accelerates to 40 rpm over 300 seconds. The time taken for the mice to fall onto the lever below is recorded as an indicator of motor co-

For Review Only

ordination. Mice were tested weekly from 7 to 20 weeks of age after habituation at week 6 (Fig. 1A). Habituation involved placing them on the cylinder at a constant speed of 4 rpm, before accelerating to 40 rpm across 300 seconds. Mice that clung to the rotating rod or changed orientation during the test, not turning back and/or getting back to the upright walk for 3 consecutive turns, were removed from the apparatus, and the maximum score was assigned.

Clasping

Mice were assessed weekly for clasping as a phenotypic characteristic specific to HD transgenic mice (relative to their WT littermate controls) independent of motor co-ordination[11] from 7 to 20 weeks of age (Fig. 1A). Clasping is identified by the retraction of paws during tail suspension and appears independent of deficits in motor co-ordination and locomotion. Mice are suspended by their tails for 30 seconds per session, while observing the clasping action of the paws during this time. Mice were scored on a five-point scale from 0 to 4, with a score of zero indicating no clasping and a score of 1-4 indicating the number of clasping paws, with the highest score recorded. The experimenter was blinded to experimental groups during scoring.

For Review Only

Digigait

At 14 weeks of age, mice were assessed using Digigait (Mouse Specifics Inc., Boston, MA, USA) for the assessment of gait and locomotion[47] (Fig. 1A). Mice are placed inside a plexiglass container on top of a transparent treadmill and allowed to habituate for 1 min. Mounted below the belt is a digital video camera that records the mice paws during treadmill locomotion. The treadmill was accelerated to 15 cm/s. Once the mouse was walking consistently for 2-5 seconds, 4-10 footsteps (or 3 seconds walking) were recorded and analysed, after which the mouse was removed from the testing chamber and returned to their home cage.

Analysis software was used to determine when individual paws are in contact with the belt of the treadmill to calculate several gait parameters. The gait parameters analysed were propelling time (the duration between maximum paw contact to the start of the swing phase) and braking time (the duration between the beginning of the swing phase and returning to maximum paw contact with the belt). The propel-to-brake ratio was then calculated.

Behavioural Testing of Cognitive Function

Y-Maze

For cognitive assessments, we used Y-maze as a short-term spatial learning and memory test[48] (Fig. 2A) at 12 weeks of age. The Y-maze is composed of three arms (10 cm wide, 30 cm long and 17 cm high) with visual cues at the end of each arm. Mice were housed in the testing room for at least one hour before the test (for all behavioural tests). In the initial trial, one arm of the maze was closed (novel arm) using a divider and mice were placed in another arm (home arm) and allowed to explore the home and another available arm (familiar arm) for ten minutes. After the initial trial, mice were placed in a holding cage for one hour and following this interval the mice were returned to the Y-maze for the second test trial, where they had free access to all three arms of the maze for five minutes. Tracking of animal movements was performed using Topscan Lite (CleverSys Inc., Reston, Virginia, USA) tracking software and the time spent exploring the novel arm was recorded as a measure of short-term memory.

Fear Conditioning & Extinction Learning

At 13 weeks of age the mice underwent fear conditioning to assess their learning capabilities in concurrence with extinction learning, testing longer-term associative cognitive function[49] (Fig. 2C). Fear conditioning and extinction learning were conducted as previously described[49,50]. The mice were placed individually into fear conditioning chambers (Med

For Review Only

Associates Inc., Fairfax, VT, USA) with stainless steel rod floors. The chambers contained either a patterned background and paper towel bedding or a plain background and clean bedding, the same type used in the home cages, to create different environments. For fear conditioning, a conditioned stimulus (CS) of an auditory tone (80dB, 5000 Hz, 10s) was paired with an unconditioned stimulus (US) of a foot shock (electric shock, 0.6 mA, 1s). The mice were habituated for 2 minutes, while their freezing (locomotor immobility) was measured (this period was considered the conditioning baseline), then the mice received 6 paired CS-US with the tone lasting 10 seconds (s) and co-terminating with a 1s foot shock. The inter-trial interval was 110 seconds. Freezing was analysed during the 9s before the onset of the foot-shock to ensure the freezing was a conditioned response to the tone. Following the last presentation of the tone, the mice were left within the chamber for 2 minutes before being returned to their home cages.

The following day their memory of the CS was tested in a different environmental context within the experimental chamber, to ensure the chamber environment was not paired with the tone in the CS. Mice were allowed to habituate in the chamber for 2 minutes, while their freezing measurement were analysed (this period was considered the extinction baseline) before being exposed to 45 presentations of the 10s tone without the shock with an inter-trial interval of 10s. Percentage freezing during the 10s tone presentation was analysed and presented as averages of nine blocks of 5 CSs to track extinction learning. The recorded freezing of the mice during fear conditioning and extinction learning was analysed using VideoFreeze (Med Associates Inc.).

For Review Only

Gastrointestinal Measures

Fecal Output & Fecal Water Content

We analysed fecal water content as a measure of gastrointestinal water absorption and fecal output and gut transit time markers of gut function and motility[17]. At 12 weeks of age, mice were single housed in a sterile cage for 1 hour and the number of excreted pellets was recorded as fecal output. These pellets were collected, and the total weight was recorded before being dried at 95°C for > 3 hours[17]. The difference between the initial feces weight and the dry weight was recorded as a percentage and taken as the fecal water content.

Gut Transit Time

At 12 weeks of age, non-fasting mice were gavaged with non-absorbable carmine red dye (Sigma-Aldrich), prepared as a 6% (w/v) dilution in 0.5% methylcellulose (Sigma-Aldrich), autoclaved and filtered before administration. Mice were single housed, and the time taken from gavage to the first appearance of carmine red was recorded as the gastrointestinal transit time.

Gut Permeability

At 14 and 20 weeks of age, mice were fasted for 4 hours, then orally gavaged with 150 µL of 4 kDa fluorescein isothiocyanate (FITC) dextran (Sigma-Aldrich) dissolved in PBS to a concentration of 100 mg/mL to measure intestinal epithelial barrier permeability[51]. Blood was collected via cardiac puncture 4 hrs after administration and immediately transferred to an EDTA collection tube and centrifuged at 1,000 × g for 10 min. Plasma was then collected, and fluorescence was quantified at an excitation wavelength of 485 nm and an emission wavelength of 528 nm (PHERAstar *FSX*, Millipore). FITC-Dextran serially diluted in PBS was used to calculate a standard curve.

Macroscopic Measures

For Review Only

Gut macroscopy, including cecum and colon measurements, was assessed as an indicator of general gut health[7]. At 14 and 20 weeks of age, mice were euthanised by cervical dislocation, the intestines were removed and placed on a non-absorbent surface and the length of the cecum and colon was measured using a ruler. The cecum was then weighed and normalised to body weight (g).

SCFA and BCFA Extraction and Analysis

Mouse plasma samples were taken at 14 and 20 weeks of age and stored at -80˚C prior to analysis. The extraction of short chain fatty acids (SCFAs) and branched-chain fatty acids (BCFAs) were performed using a modified protocol[52]. Briefly, 20 µL of plasma was suspended in 380 µL of water: acetonitrile (1:1. v/v) containing 4 µM of 4-methylvaleric acid (product # 277827, Sigma-Aldrich, Australia) as internal standard. Samples were vortexed for 30s and then mixed at 950 rpm for 10 min at 4˚C with a thermomixer (Eppendorf, Macquarie Park, Australia). Samples were centrifuged at 16,200 g (Beckman Coulter Microfuge 22R refrigerated microcentrifuge) for 5 min at 4 ˚C and the supernatant transferred to fresh LoBind Eppendorf tubes. Stock solutions of all the SCFAs and BCFAs were freshly and individually prepared in 100% aqueous acetonitrile with 1mM concentration. This solution was further diluted to have concentrations of 50 µM to 0.1 µM and the resulting solutions were used as the calibrators. The calibrators (40 µL) and sample supernatant (40 µL) were mixed with 20 µL of 200mM 3-nitrophenylhydrazine (NPH, Product # N21804, Sigma-Aldrich) in 50% aqueous acetonitrile and 20 µL of 120 mM 1-Ethyl-3-(3-dime-thylaminopropyl) carbodiimide (EDC, Product # E7750, Sigma-Aldrich) in 50% aqueous acetonitrile with 6% pyridine solution. The mixture was reacted at 40 ˚C for 30 min in a thermomixer at 950 rpm. The reaction was stopped using 20 µL of 200 mM Quinic acid (Product # 46944, Sigma-Aldrich) dissolved in acetonitrile: water (1:1, v/v) by further incubating the mixture at 40 ˚C for 30 min in a

thermomixer at 950 rpm. Finally, the samples were then spiked with 20 µL of 10 µM isotope-labelled internal standard mix prepared in accordance with the previously described protocol[52]. The samples were further diluted to 2 mL with 15% acetonitrile and 1 uL were then injected for LC-MS/MS analysis. Derivatized SCFAs and BCFAs were processed and detected by Metabolomics Australia (Bio21 Institute, Melbourne, VIC, Australia) using the conditions previously described (1, 2) using Agilent 1290 liquid chromatography (LC) system and Agilent Triple quadrupole 6490 mass spectrometer (Agilent Technologies, Mulgrave, Australia).

Gut Immune Profile

Proximal colon samples from 14 and 20 weeks of age, were stored at -80˚C prior to analysis. Samples were sonicated in 300 µL cell lysis buffer (Invitrogen) and refrozen. The thawed suspension was spun for 5 mins at 3000rpm, and the supernatant was assayed for cytokines as per manufacturers recommendations. The ProcartaPlex multiplex Immunoassay (Invitrogen) was used to determine the Th17 panel of cytokines (IFNγ, IL-17A, IL-17E, IL-1β, IL-21, IL-22, IL-6, IL-7R and TNF-α) using Luminex® 200 System. The DC protein assay kit (Bio-rad laboratories, USA) was used determine the protein content. Groups that had less 4 samples above the limit of the detection were excluded from the analysis. Results were normalised to supernatant protein concentration and are expressed as pg/mg of protein.

Fecal DNA Extraction and 16S rRNA Sequencing

Fecal samples were collected at 8 (pre-interventions), 12 (3 weeks post-ATB, 2 weeks post-FMT) and 20 (11 weeks post-ATB, 10 weeks post-FMT) weeks of age (Fig. 1A). Mice were placed in sterile individual cages for fecal pellet collection. Due to COVID-19 pandemic restrictions (lockdown) at the time, we could not collect fecal samples at 8 weeks of age from

For Review Only

the female cohorts that underwent the full experimental design and received all the interventions. Fecal pellets from an extra cohort of 8-week-old WT and HD females were added to the study instead. Fresh fecal pellets were placed into 1.5 mL Eppendorf tubes aseptically, immediately frozen with dry ice, followed by storage at -80°C prior to analysis (up to six months with no freeze-thaw cycle). Fecal pellets underwent DNA extraction by a combination of mechanical and chemical lysis method with using a DNeasy PowerLyzer PowerSoil kit (QIAGEN, Hilden, Germany, according to the manufacturer's instructions with modifications as previously described[32].

Extracted DNA was used for amplicon sequencing of the V4 hypervariable region (515 – 806bp) of the 16S rRNA gene on a Miseq Illumina platform as previously described[53]. Paired-end sequencing (2 x 300bp) of indexed amplicon libraries were performed using a Miseq reagent kit v3 and Miseq system (Illumina Inc, San Diego, USA) at the South Australian Genomics Centre (SAGC), Adelaide, Australia. Microbiota profiling was performed based on a subsampling depth of 9,604 or 12,777 sequence reads for the male or female cohort, respectively.

For Review Only

Bioinformatics Analysis

Paired-end 16S rRNA gene sequence reads were analysed using QIIME2 (v2.0)[54]. Briefly, the DADA2 workflow was used for denoising, quality filtering, chimera removal and merging of paired-end sequence reads[55]. Taxonomic assignment of amplicon sequence variants (ASVs) was performed against the V4 hypervariable region sequences of the SILVA 132 16S rRNA reference database clustered at 97% similarity[56]. Alpha diversity (observed species, Faith's phylogenetic diversity) and weighted Unifrac distances were computed using QIIME2. Genus-level relative abundances were used for downstream analysis.

Statistical Analysis for 16S rRNA Sequencing

Non-parametric analyses were performed using the Mann-Whitney test to compare two groups, or the Kruskal-Wallis test with *post-hoc* Dunn's test for three or more groups. Microbiota composition differences between groups were assessed based on weighted Unifrac distances and analysed using permutational ANOVA (PERMANOVA). Correction of multiple testing was performed using the false discovery rate (FDR) method. Sample ordination and analysis was performed using GraphPad Prism 9 or R (package vegan 2.5-7).

For Review Only

Statistical analysis

GraphPad Prism (version 9.2.0) was used to plot the graphs and the data analysis was performed using R studio (version 1.3.1093). Data is presented as the mean ± standard error of the mean (SEM). Linear mixed models (LMM) were used to analyse the data separately for males and females. Cumulative LMM with Laplace approximation was used for clasping scores that was measured on an ordinal scale. For covariates declared significant at the 0.05 significance level, we performed a *post-hoc* pairwise comparisons (emmeans R package) (Supplementary tables 1-34).

In LMM cage effects were specified as random effects in LMM due to potential effect of coprophagy. For single time point variables (e.g. brain weight), genotype and treatment (No treatment, ATB, ATB/FMT) were specified as fixed effects. For Y-maze, arm type (novel, familiar) was added as fixed effect. For repeated measurements variables (e.g. body weight), time was also added as a fixed effect with the analysis being conducted from week 8, when interventions started. For contextual fear conditioning (CFC), baseline CFC conditioning and baseline CFC extinction were added as fixed effects, respectively. We considered all two-way interactions between fixed effects when there were at least four samples in each group

combination, otherwise only the main effects were considered (as was the case for males for the Acetate variable in week 14).

Data availability

All data, including 16S rRNA amplicon sequencing data, will be made fully available upon publication. Raw sequence data is deposited in the public repository Sequence Read Archive (SRA) under the Bioproject accession number PRJNA795813. The data analysis R code is available at https://github.com/SarithaKodikara/Fecal-microbiota-transplant-therapeutic-effect-in-a-Huntington-s-disease-mouse-model.

For Review Only

Results

ATB/FMT did not improve motor deficits and related outcomes in HD mice

Impairment in motor performance, decreased body and brain weight and increased water intake are the most consistent markers of preclinical HD progression (Fig. 1A). Our study recapitulated all these phenotypic characteristics in the R6/1 transgenic mouse model of HD. When compared to WT, both male and female HD mice showed a decreased body weight over time ($p<0.0001$) (Fig. 1B), despite an increasing food intake in HD males ($p=0.01$) and HD females ($p=0.003$) over time (Fig. 1C). In males, ATB decreased body weight ($p<0.0001$). In HD mice, ATB increased body weight ($p=0.0002$) and decreased food intake ($p<0.0001$). ATB/FMT increased body weight in females over time ($p=0.005$) and there was a trend to increase body weight in males over time ($p=0.052$), as well as a significant increase in food intake in males ($p=0.0004$). Conversely, ATB/FMT induced a decreased food intake ($p=0.0002$). Both male and female HD mice showed an increase in water intake over time

($p<0.0001$ and $p<0.0001$, respectively), while ATB increased the water intake compared to the no treatment (no FMT nor ATB) mice ($p=0.01$, in males only) over time (Fig. 1D).

Both male and female HD mice showed an impaired motor performance on the rotarod over time ($p<0.0001$ for both) (Fig. 1E). For both sexes, we observed an overall increase in clasping scores, a marker of HD disease progression, as the mice aged (Fig. 1G), as well as an increase in clasping score for HD mice compared to WT ($p<0.0001$ for both) (Fig. 1G). Apart from an effect of ATB/FMT in males inducing an unexpected general decrease in the latency to fall off the rotarod over time ($p=0.0116$), ATB and ATB/FMT were not able to modulate either rotarod or clasping performance. Digigait analysis (assessment of gait and locomotion) in males only revealed an increase in propel:brake ratio in HD mice ($p=0.004$) and a decrease in ATB-treated HD mice ($p=0.01$) (Fig. 1F). Finally, at 20 weeks of age, a late stage in the disease, we observed a decrease in brain weight as a measure of gross neurodegeneration in HD males only ($p=0.003$), with no effect of ATB or ATB/FMT (Fig. 1H).

For Review Only

FMT modulated cognitive outcomes in HD

Short-term spatial learning and memory were assessed based on Y-maze performance (Fig. 2A). As expected, both male and female HD mice performed worse on this cognitive test when compared to WT ($p=0.04$ and $p=0.004$, respectively) (Fig. 2B). Interestingly in females, *post-hoc* testing indicated that in WT, the difference observed between arms in the control group is not present in the ATB group and appears again after the ATB/FMT treatment (Supplementary table S1). On the other hand, ATB/FMT did not modulate HD performance in this test.

We also conducted contextual fear-conditioning testing, by assessing the freezing response, or the absence of movement apart from respiration, as an indicator of cognitive function (Fig. 2C). In the conditioning trial we observed an effect of the conditioned stimulus (CS) in both males and females ($p<0.0001$) indicating that the CS and unconditioned stimulus

(US) pairing induces conditioning learning (Fig. 2D). Furthermore, in males only, we saw an effect at the baseline time point (p=0.0002), as well as an interaction between the baseline and ATB/FMT, overall decreasing the percentage of freezing, suggesting a positive effect of FMT on basal anxiety. At the extinction trial, we saw a decrease in the percentage of freezing in both males and females by time (p=0.0002 and p<0.0001, respectively), indicating that in general the extinction occurred in the test (Fig. 2E). As previously reported, we found a decrease in extinction in both HD males (p<0.0001) and females (p=0.0005) over time compared to WT. We also observed an effect at the baseline time point in both males and females (p<0.0001) with an increased percentage of freezing, while an interaction demonstrated a decrease in freezing over time in both males and females (p<0.0001 and p=0.01, respectively). Also, in female HD mice only, we observed a decrease in freezing behaviour at the baseline time point (p=0.0005) and an interaction between ATB/FMT and the baseline (p=0.009). ATB in females increased the percentage of freezing over time (p=0.01). *Post-hoc* analysis indicated that in males, there was a decrease in extinction in HD mice subjected to ATB/FMT when compared to WT mice subjected to ATB/FMT (Supplementary table S2**)**. In females on the other hand, *post-hoc* analysis indicated a decrease in extinction in HD mice in the control group and in the ATB group but not between the mice subjected to the ATB/FMT intervention (Supplementary table S3**)**.

Gut dysfunction is a characteristic of the HD phenotype and ATB/FMT did not modulate its progression

In the present study, we have investigated gut structure and function of adult-onset R6/1 HD mice over time, including at early (14 weeks of age) and late (20 weeks of age) stages of the disease, while assessing the effect of ATB and ATB/FMT on these outcomes. Despite observing an increased food and water intake in HD mice compared to WT, both male and

For Review Only

female HD mice showed a decreased fecal output, an indirect measurement of constipation as they aged ($p<0.0001$, for both). In males we saw a trend towards ATB increasing fecal output in HD only ($p=0.056$), while ATB increased the fecal output of both WT and HD females ($p=0.01$) (Fig. 3A). HD females showed an overall decrease in fecal water content ($p=0.04$). ATB increased the fecal water content in HD males ($p<0.0140$) while ATB/FMT over time induced a decrease in fecal water content in both males ($p=0.03$) and females ($p=0.01$) (Fig. 3B). While the fecal output and fecal water content results indicate constipation in the HD mice, when gut transit time was assessed, apart from a trend of ATB/FMT to induce a decrease in the gut transit time in females ($p=0.054$), no differences were observed at week 14 or 20 for either sex (Supplementary Fig. 1A, B). Since a leaky gut has been identified in R6/2 HD mice[8], we investigated the intestinal permeability in our R6/1 HD model. No differences at the early disease stage (week 14) in HD mice were observed, when compared to WT (Supplementary Fig. 1C), consistent with our recent findings[12]. However, an increase in gut permeability at the late disease stage (week 20) was detected in both male and female HD mice compared to WT littermate controls ($p=0.02$ and $p=0.03$, respectively) (Fig. 3C). ATB and ATB/FMT interventions were not able to modulate this outcome.

We investigated the gut macroscopic structure of the HD mice and found no genotype differences at 14 weeks of age (Fig. 3E, F, Supplementary Fig. 1D), indicating no gut gross structural changes at this early stage of the disease. However, at the late disease stage (20 weeks of age) we did observe a decreased colon length in HD males compared to WT littermate controls ($p=0.008$) (Fig. 3D). Apart from male WT and HD mice showing a decrease in cecum weight induced by ABT/FMT treatment ($p=0.03$) (Fig. 3E) we did not see any short-term or long-term effect of ATB or ATB/FMT on other gut outcomes, including cecum and colon length at week 14, and cecum weight and length at week 20 (Fig. 3F, Supplementary Fig. 1D, E, F). Altogether, these results indicate the presence of gut dysfunction in the late stage of the

HD phenotype as well as sexual dimorphism, with HD males being slightly more affected. Moreover, neither ATB nor FMT resulted in significant gut-related modulation.

HD males develop gut microbiome disruption including abnormally high microbiome instability at an early stage of the disease

In determining the potential for early-stage prevention of gut microbiome disruption (*via* FMT intervention) prior to the onset of gut dysfunction, the gut microbiota of male and female HD mice were characterised at 8 weeks of age, using fecal 16S rRNA sequencing and bioinformatics. Reductions in fecal total bacterial load (Supplementary Fig. 2A), and changes to the structure and composition of the gut microbial community were observed for HD males, but not in females. Specifically, alpha diversity measures of microbial richness (observed species, p=0.002) (Supplementary Fig. 2B) and diversity were reduced (Faith's phylogenetic diversity, p=0.007) (Supplementary Fig. 2C), while composition of the gut microbiota was significantly altered in HD males compared to WT littermate controls (PERMANOVA p=0.0002) (Supplementary Fig. 2D). In contrast, gut microbiota alterations were less marked in HD females compared to WT. Specifically, alpha diversity indices of microbial richness (p=0.613) and Faith's phylogenetic diversity (p=0.779), as well as the gut microbiota composition (PERMANOVA p=0.634), were similar between HD and WT females.

Homogeneity of the microbiota composition among HD male and female mice at the early stage of disease (week 8) were significantly lower compared to those of WT littermate controls (PERMDISP p=0.042 and p=0.034, respectively). These results were consistent with significantly larger microbiota variation within HD mice compared to the WT mice at week 8 (males, p<0.0001 and females, p=0.046, respectively) (Supplementary Fig. 3), with a higher

For Review Only

magnitude of variation observed for HD males (median [IQR]= 0.23[0.14, 0.27]) compared to females (0.16[0.09, 0.24]).

HD male mice show lack of engraftment of WT gut microbiota following FMT intervention

FMT intervention was performed during the early stages of the disease to determine whether colonisation of WT gut microbiota in HD mice could delay the onset of disease and associated phenotypic features. Donor microbiota engraftment was subsequently determined by fecal microbiota analysis of WT and HD groups at week 12 and week 20. For both the WT and HD genotypes in males, fecal bacterial load of ATB or FMT mice were similar to those receiving the vehicle by week 12 and week 20 (Mann-Whitney test, $p>0.05$) (Fig. 4A). In females, fecal bacterial load at week 12 was significantly higher in WT and HD mice that received FMT ($p=0.039$ and $p=0.002$, respectively) when compared to the levels in the untreated WT group (Fig. 4A). Similar to males, the levels of fecal bacterial load in females at 20 weeks of age were similar to those receiving the vehicle.

For both WT and HD males that received antibiotics, reductions in the number of species observed ($p=0.005$ and $p=0.007$, respectively) (Fig. 4B) and compositional alterations (PERMANOVA $p=0.004$ for both WT and HD genotypes) (Fig. 5A) were observed at 12 weeks of age when compared to those that received the vehicle. FMT reversed these effects in WT males (microbial richness, $p=0.366$; composition, PERMANOVA $p=0.445$) but not in HD males (microbial richness, $p=0.0009$; composition, PERMANOVA $p=0.004$) (Fig. 4B, Fig. 5B). At 20 weeks of age, microbial alpha diversity of HD mice that received antibiotics or FMT did not significantly differ from the WT mice (Fig. 4B-C), and compositional differences associated with gut dysbiosis almost disappeared (ATB $p=0.049$; FMT $p=0.040$) (Fig. 5A). In contrast, FMT was able to fully restore the gut microbiome composition of ATB-treated female

For Review Only

HD mice to resemble that of the untreated WT mice ($p>0.05$). Together, these results suggest that wild-type gut microbiota engraftment in HD mice was more effectively established in females compared to males.

HD males have increased plasma acetate and colon IL7R levels while female HD mice show a decrease in colon IFNg levels

We assessed the plasma levels of short-chain fatty acids (SCFAs) and branched-chain fatty acids (BCFAs), which are known to be produced by gut microbiota, and found an increase in acetate levels in HD males at 14 weeks of age compared to WT mice ($p=0.04$) (Fig. 6A). At 20 weeks of age, we saw an increase in acetate levels induced by ATB in HD males ($p=0.04$) (Fig. 6B) and an effect of ATB/FMT on propionate levels in females ($p=0.04$) (Fig. 6C). We did not see any further significant difference in any other SCFA or BCFA levels (Supplementary tables S23-S26**)**.

We assessed the gut immune status by analysing cytokines levels in proximal colon. Interestingly, at 14 weeks of age, we found a decrease in IFNγ levels in female HD mice ($p=0.02$) (Fig. 7A) and, at 20 weeks of age, we saw a trend for male HD mice to have decreased IFNγ levels ($p=0.059$) (Fig. 7B). In females, we found a trend towards a decrease of IL6 levels induced by ATB at at 14 weeks of age ($p=0.059$) (Fig. 7C). However, at 14 weeks of age in males, FMT increased IL17E levels ($p=0.04$) (Fig. 7D) and IL7R was found to be increased in HD mice ($p=0.01$) (Fig. 7E). No significant differences in levels of any other cytokine were observed (Supplementary tables S27-S34).

Discussion

For Review Only

This is the first study to investigate the therapeutic potential of FMT as a means to shift the recently discovered pathological HD gut microbiome characteristics (dysbiosis) towards a healthy profile. FMT modulated cognitive outcomes in HD, an effect that was more pronounced in females. This study reveals an inefficiency of engraftment of the WT microbiota in HD male mice and an associated sexually dimorphic disruption and instability of the gut microbial community, circulating SCFA levels and gut immune profiles in HD. We report, for the first time, significant gut dysfunction in the late stage of the HD phenotype. Sexual dimorphism was also observed for this trait, with HD males being more affected. Our findings, together with those of others, suggest that HD is not just a brain disorder but rather is a whole-body disease with pathological features including sexually dimorphic and progressive gut dysfunction and microbial disruption. These findings will inform future microbiota-targeted therapies for HD, to address both central and peripheral symptoms of this fatal disease.

The onset of cognitive symptoms in HD is a driving factor in the progressive decline in quality of life[23]. Improvement in learning and memory has also been one of the key targets for the gut microbial interventions for brain disorders[13,24,25]. Corroborating previous findings[9], we have found reduced novel arm preference in HD mice in the Y-maze cognitive test. Gastrointestinal inflammation and gut microbiome disruption has been shown to impair cognitive performance in the Y-maze[26] and in our study, ATB in female WT mice inhibited cognitive performance in the Y-maze, which was resolved after the FMT intervention. We have also replicated previous findings showing that HD mice exhibit decreased freezing in the extinction trial of the fear conditioning test, indicating impaired fear learning and memory[27]. Recent findings have implicated the gut microbiota in fear memory and impaired extinction learning with ATB-treated and germ-free mice showing impaired extinction learning[28]. We observed similar results in females with the ATB treatment. In males, we found a decrease in extinction induced by ATB/FMT in HD mice when compared to WT mice. However, in

For Review Only

females, the FMT intervention was able to fully rescue the cognitive deficits observed in HD mice. Altogether, these results reflect the sexually dimorphic FMT engraftment observed in HD mice and support the hypothesis that interventions that ameliorate gut microbiome disruption (dysbiosis) could in turn be therapeutic for this disease.

Successful engraftment of the gut microbiota is dependent on many factors, including genetic background, composition of the gut microbial community, inflammation status, housing environment, as well as diet of the donor and recipient individuals[29]. All variables besides the genetic and microbiota differences between our donor WT and recipient HD mice, were carefully controlled and kept consistent in the present study. We have also followed the most recent guidelines for reporting and performing animal FMT[30] and have used established protocols for both the ATB gut preparation[31] and intervention[32]. FMT engraftment was successful in both WT males and females, whereas a lower fidelity of engraftment and subsequently, lack of phenotype modulation was observed in HD mice, particularly in HD males. These results suggest the importance of further understanding complex donor-recipient dynamics in HD for the optimization of future clinical intervention strategies associated with modulating microbial-host relationships.

The stability of the gut microbiome is critical to host health, in which complex interactions between microbial species increases resilience of the microbial community to perturbation[33,34]. An unstable state involving increased heterogeneity in microbiome composition over time, which has been associated with numerous negative health outcomes[35], was observed in the present study, particularly in HD males, extending our previous study in unmanipulated R6/1 HD mice[16]. Notably, in the present study we observed that the instability of the HD gut microbiome was concurrent with its resistance to FMT engraftment. Given that the host genetics greatly influences the physical structure of the gut, which then determines the chemical and physical environment inhabited by the gut microbiome[36], one possible

For Review Only

explanation is that the expression of mutant HTT (*via* the human HD transgene) by the HD mice altered the gut mucosal lining and overall architecture, thus exerting stochastic effects on microbiome composition while impeding the colonization of microbes adapted to a healthy gut. This hypothesis should be further investigated.

Male HD mice showed increased levels of acetate (a key SCFA) in plasma at early and late stages of the disease. The major source of circulating SCFAs is the fermentation of indigestible carbohydrates by intestinal bacteria[37], and SCFAs are considered key regulators of the microbiota-gut-brain crosstalk[38]. While increases in SCFA levels are usually associated with beneficial outcomes, increased plasma acetate levels have been shown in multiple sclerosis patients compared to controls, with levels correlating with greater disability and increased T helper 17 (T_H17) + cells[39]. Interestingly, acetate is considered to be an important metabolite for host resistance to bacterial infection, having a pivotal role in the pro- and anti-inflammatory balance, with high acetate promoting superior immune control[40,41]. The gut immune profile is crucial for the host gut microbiota composition[42] and it has been recently shown to be a key modulatory factor for the efficacy of FMT in *C. difficile* infection[43]. We have found increased IL7R colon levels in HD males while HD females showed a decrease in IFNγ colon levels. Intestinal IL7R signaling from the T_H17 cell subset is present in inflamed colon tissue, closely related to inflammatory bowel disease[44]. IFNγ mediates the recognition and response to bacteria[45], both key players in the host bacterial defence and in shaping the gut microbial community. The imbalance of the acetate levels and intestinal immunity observed in male and female HD mice (a more pro-inflammatory and antimicrobial defense profile in males) could also be related to the sexual dimorphism observed in the FMT engraftment in HD. Further studies are needed to test this hypothesis. Nevertheless, our results raise opportunities to improve FMT efficacy for therapeutic intervention for both sexes in HD by focusing on modulating gut microbial stability, acetate levels and immune profiles.

For Review Only

In conclusion, our results demonstrate a potential role for gut microbiota in modulating cognitive outcomes in HD, presenting opportunities to pursue novel microbiota-targeted therapies for this devastating disease in future preclinical studies and clinical trials.

Acknowledgments

CG dedicates this study to the memory of Dr. Rafael Calixto Bortolin, who provided kind support and fruitful brainstorming sessions at the conceptualization period of this project. We thank members of the Hannan Laboratory, past and present, for useful discussions and technical advice which informed this study. The Graphical Abstract was created using software from BioRender.com.

Funding

CG is a Hereditary Disease Foundation (HDF) Fellow. TR is a National Health and Medical Research Council (NHMRC) Dementia Fellow (GNT1136529). AJH is an NHMRC Principal Research Fellow (GNT1117148) and is also supported by NHMRC Project Grants, an ARC Discovery Project and the DHB Foundation, Equity Trustees. K-A.LC was supported in part by the NHMRC Career Development fellowship (GNT1159458). YW was supported by the Chinese Scholarship Council. GBR was supported by an NHMRC grant (APP1155179) and Matthew Flinders Fellowship.

Competing interests

The authors report no competing interests.

Supplementary material

Supplementary material is available at *Brain* online.

For Review Only

Author contributions

Conceptualization and methodology, C.G., G.K., J.M.C., G.B.R. and A.J.H.; Investigation, C.G., C.J.L., B.A.M., J.J.M.L., V.K.N and J.M.C.; Statistical data analysis, C.G., S.K., Y.W. J.M.C. and K-A.L.C.; Resources, T.R., G.B.R. and A.J.H.; Writing – Original Draft, C.G., C.J.L., S.K. and J.M.C.; Writing – Review & Editing, C.G., C.J.L., J.M.C., B.A.M., S.K., Y.W., J.J.M.L., G.K., V.K.N., G.B.R., K-A.L.C., T.R. and A.J.H.; Supervision, C.G., K-A.L.C., T.R., G.B.R. and A.J.H.; Project Administration, C.G.; Funding Acquisition, G.B.R. and A.J.H. All authors approved the final version of the manuscript.

For Review Only

References

1. Wyant KJ, Ridder AJ, Dayalu P. Huntington's Disease—Update on Treatments. *Curr Neurol Neurosci Rep.* 2017;17:48109.
2. McColgan P, Tabrizi SJ. Huntington's disease: a clinical review. *Eur J Neurol.* 2018;25:24–34.
3. Schulte J, Littleton JT. The biological function of the Huntingtin protein and its relevance to Huntington's Disease pathology. *Curr Trends Neurol.* 2011;5:65–78.
4. Li SH, Schilling G, Young WS. et al. Huntington's disease gene (IT15) is widely expressed in human and rat tissues. *Neuron.* 1993;11:985–993.
5. Nance MA, Sanders G. Characteristics of individuals with Huntington disease in long-term care. *Mov Disord.* 1996;11:542–548.
6. van der Burg JMM, Gardiner SL, Ludolph AC, Landwehrmeyer GB, Roos RAC, Aziz NA. Body weight is a robust predictor of clinical progression in Huntington disease. *Ann Neurol.* 2017;82:479–483.
7. Van Der Burg JMM, Winqvist A, Aziz NA, et al. Gastrointestinal dysfunction contributes to weight loss in Huntington's disease mice. *Neurobiol Dis.* 2011;44:1–8.
8. Stan TL, Soylu-Kucharz R, Burleigh S, et al. Increased intestinal permeability and gut dysbiosis in the R6/2 mouse model of Huntington's disease. *Sci Rep.* 2020;10:1–9 ().
9. Nithianantharajah J, Barkus C, Murphy M, Hannan AJ. Gene-environment interactions modulating cognitive function and molecular correlates of synaptic plasticity in Huntington's disease transgenic mice. *Neurobiol Dis.* 2008;29:490–504.
10. Spires TL, Grote HE, Varshney NK, et al. Environmental Enrichment Rescues Protein Deficits in a Mouse Model of Huntington's Disease, Indicating a Possible Disease Mechanism. *J Neurosci.* 2004;24:2270–2276.
11. Van Dellen A, Blakemore C, Deacon R, York D, Hannan AJ. Delaying the onset of Huntington's in mice. *Nature.* 2000;404:721–722.
12. Gubert C, Love CJ, Kodikara S, et al. Gene-environment-gut interactions in

Huntington's disease mice are associated with environmental modulation of the gut microbiome. *iScience* 2021;25(1):103687.

13. Cryan JF, O'Riordan KJ, Cowan CSM, et al. The Microbiota-Gut-Brain Axis. *Physiol Rev.* 2019;99:1877–2013.
14. Dinan TG, Cryan JF. Gut instincts: microbiota as a key regulator of brain development, ageing and neurodegeneration. *J Physiol.* 2017;595:489–503.
15. Sarkar A, Harty S, Lehto SM, et al. The Microbiome in Psychology and Cognitive Neuroscience. *Trends Cogn Sci.* 2018;22:611–636.
16. Kong G, Ellul S, Narayana VK, et al. An integrated metagenomics and metabolomics approach implicates the microbiome-gut-brain-axis in the pathogenesis of Huntington's disease transgenic mice. *Neurobiol Dis.* 2020;148:105199.
17. Kong G, Cao KL, Judd LM, Li S, Renoir T, Hannan AJ. Microbiome profiling reveals gut dysbiosis in a transgenic mouse model of Huntington's disease. *Neurobiol Dis.* 2018; 135:104268.
18. Wasser CI, Mercieca EC, Kong G, et al. Gut dysbiosis in Huntington's disease: associations between gut microbiota, cognitive performance and clinical outcomes. *Brain Commun.* 2020;2(2):fcaa110.
19. Du G, Dong W, Yang Q, et al. Altered Gut Microbiota Related to Inflammatory Responses in Patients With Huntington's Disease. *Front Immunol.* 2021;11:1–12.
20. Green JE, Davis JA, Berk M, et al. Efficacy and safety of fecal microbiota transplantation for the treatment of diseases other than Clostridium difficile infection: a systematic review and meta-analysis. *Gut Microbes.* 2020;12:1–25.
21. Kang DW, Adams JB, Gregory AC, et al. Microbiota Transfer Therapy alters gut ecosystem and improves gastrointestinal and autism symptoms: An open-label study. *Microbiome.* 2017;5:22082–22090.
22. Kim MS, Kim Y, Choi H, et al. Transfer of a healthy microbiota reduces amyloid and tau pathology in an Alzheimer's disease animal model. *Gut.* 2020;69:283–294.
23. Roos RA. Huntington's disease: a clinical review. *Orphanet J Rare Dis.* 2010;5:40.
24. Desbonnet L, Clarke G, Traplin A, et al. Gut microbiota depletion from early adolescence in mice: Implications for brain and behaviour. *Brain Behav Immun.* 2015; 48:165-73.
25. Savignac HM, Tramullas M, Kiely B, Dinan TG, Cryan JF. Bifidobacteria modulate cognitive processes in an anxious mouse strain. *Behav Brain Res.* 2015;287:59–72.
26. Jang SE, Lim SM, Jeong JJ, et al. Gastrointestinal inflammation by gut microbiota disturbance induces memory impairment in mice. *Mucosal Immunol.* 2018;11:369–379.
27. Zhang H, Zhang C, Vincent J, et al. Modulation of AMPA receptor surface diffusion restores hippocampal plasticity and memory in Huntington's disease models. *Nat Commun.* 2018;9(1):4272.
28. Chu C, Murdock MH, Jing D, et al. The microbiota regulate neuronal function and fear extinction learning. *Nature.* 2019;574:543–548.
29. Danne C, Rolhion N, Sokol H. Recipient factors in faecal microbiota transplantation: one stool does not fit all. *Nat Rev Gastroenterol Hepatol* 2021;18(7):503-513.
30. Secombe KR, Al-Qadami GH, Subramaniam CB, et al. Guidelines for reporting on animal fecal transplantation (GRAFT) studies: recommendations from a systematic review of murine transplantation protocols. *Gut Microbes.* 2021;13(1):1979878.
31. Staley C, Kaiser T, Beura LK, et al. Stable engraftment of human microbiota into mice with a single oral gavage following antibiotic conditioning. *Microbiome.* 2017;5:87.
32. Choo JM, Rogers GB. Establishment of murine gut microbiota in gnotobiotic mice. *iScience* 2021;24:102049.

For Review Only

33. Lozupone CA, Stombaugh JI, Gordon JI, Jansson JK, Knight R. Diversity, stability and resilience of the human gut microbiota. *Nature* 2012;489:220–230.
34. Faith JJ, Guruge JL, Charbonneau M, et al. The long-term stability of the human gut microbiota. *Science.* 2013;341(6141):1237439.
35. Ma Z. Testing the Anna Karenina Principle in Human Microbiome-Associated Diseases. *iScience.* 2020;23:101007.
36. Hall AB, Tolonen AC, Xavier RJ. Human genetic variation and the gut microbiome in disease. *Nat Rev Genet.* 2017;18:690–699.
37. Bose S, Ramesh V, Locasale JW. Acetate Metabolism in Physiology, Cancer, and Beyond. *Trends Cell Biol.* 2019;29:695–703.
38. Dalile B, Van Oudenhove L, Vervliet B, Verbeke K. The role of short-chain fatty acids in microbiota–gut–brain communication. *Nat Rev Gastroenterol Hepatol.* 2019;16:461–478.
39. Pérez-Pérez S, Domínguez-Mozo MI, Alonso-Gómez A, et al. Acetate correlates with disability and immune response in multiple sclerosis. *PeerJ.* 2020;8:1–14.
40. Balmer ML, Ma EH, Bantug GR, et al. Memory CD8+ T Cells Require Increased Concentrations of Acetate Induced by Stress for Optimal Function. *Immunity* 2016;44:1312–1324.
41. Balmer ML, Ma EH, Thompson AJ, et al. Memory CD8+ T Cells Balance Pro- and Anti-inflammatory Activity by Reprogramming Cellular Acetate Handling at Sites of Infection. *Cell Metab.* 2020;32:457-467.e5.
42. Zheng D, Liwinski T, Elinav E. Interaction between microbiota and immunity in health and disease. *Cell Research.* 2020;30:492–506.
43. Littmann ER, Lee JJ, Denny JE, et al. Host immunity modulates the efficacy of microbiota transplantation for treatment of Clostridioides difficile infection. *Nat Commun.* 2021;12:1–15.
44. Belarif L, Danger R, Kermarrec L, et al. IL-7 receptor influences anti-TNF responsiveness and T cell gut homing in inflammatory bowel disease. *J Clin Invest.* 2019;129:1910–1925.
45. Boxx GM, Cheng G. The Roles of Type i Interferon in Bacterial Infection. *Cell Host Microbe* 2016;19:760–769.
46. Brooks SP, Janghra N, Workman VL, Bayram-Weston Z, Jones L, Dunnett SB. Longitudinal analysis of the behavioural phenotype in R6/1 (C57BL/6J) Huntington's disease transgenic mice. *Brain Res Bull.* 2012;88:94–103.
47. Wright DJ, Renoir T, Smith ZM, et al. N-Acetylcysteine improves mitochondrial function and ameliorates behavioral deficits in the R6/1 mouse model of Huntington's disease. *Transl Psychiatry* 2015;5:e492-10.
48. Pang TYC, Stam NC, Nithianantharajah J, Howard ML, Hannan AJ. Differential effects of voluntary physical exercise on behavioral and brain-derived neurotrophic factor expression deficits in huntington's disease transgenic mice. *Neuroscience* 2006;141:569–584.
49. Short AK, Yeshurun S, Powell R, et al. Exercise alters mouse sperm small noncoding RNAs and induces a transgenerational modification of male offspring conditioned fear and anxiety. *Transl Psychiatry.* 2017;7(5):e1114.
50. Handford CE, Tan S, Lawrence AJ, Kim JH. The effect of the mGlu5 negative allosteric modulator MTEP and NMDA receptor partial agonist D-cycloserine on Pavlovian conditioned fear. *Int J Neuropsychopharmacol.* 2014;17:1521–1532.
51. Woting A, Blaut M. Small intestinal permeability and gut-transit time determined with low and high molecular weight fluorescein isothiocyanate-dextrans in C3H mice. *Nutrients.* 2018;10:4–10.

For Review Only

52. Han J, Lin K, Sequeira C, Borchers CH. An isotope-labeled chemical derivatization method for the quantitation of short-chain fatty acids in human feces by liquid chromatography-tandem mass spectrometry. *Anal Chim Acta* 2015;854:86–94.
53. Choo JM, Kanno T, Zain NM, et al. Divergent Relationships between Fecal Microbiota and Metabolome following Distinct Antibiotic-Induced Disruptions. *mSphere* 2017;2(1):e00005-17.
54. Bolyen E, Rideout JR, Dillon MR, et al. Author Correction: Reproducible, interactive, scalable and extensible microbiome data science using QIIME 2. *Nat Biotechnol.* 2019;37:1091.
55. Callahan BJ, McMurdie PJ, Rosen MJ, Han AW, Johnson AJ, Holmes SP. DADA2: High-resolution sample inference from Illumina amplicon data. *Nat Methods* 2016;13:581–583.
56. Quast C, Pruesse E, Yilmaz P, et al. The SILVA ribosomal RNA gene database project: Improved data processing and web-based tools. *Nucleic Acids Res.* 2013;41, 590–596.

For Review Only

Figure legends

Figure 1 Effects of ATB and ATB/FMT on the phenotypic expression of HD mice. (**A**) Study design illustrating weekly measurements, motor and gastrointestinal measurements and behavioural testing as well as ATB intervention at 8 weeks and FMT intervention at 9 weeks. (**B**) Body weight (g) in WT and HD mice with ATB and ATB/FMT interventions from 7 to 20 weeks of age (males n=8-26, females n=6-14). (**C**) Food intake in WT and HD mice with ATB and ATB/FMT interventions from 9 to 20 weeks of age as gram-to-gram ratio (males n=3-6 cages, females n=2-7 cages; 2-3 mice per cage). (**D**) Water intake in WT and HD mice with ATB and ATB/FMT interventions from 10 to 20 weeks of age as mL-to-gram ratio (males n=4-5 cages, females n=2-7 cages; 2-3 mice per cage). (**E**) Latency to fall (s) off rotarod in WT and HD mice with ATB and ATB/FMT interventions from 6 to 20 weeks of age (males n=8-26, females n=6-14). (**F**) Digigait propel:brake ratio in WT and HD mice with ATB and ATB/FMT interventions at 14 weeks of age (males n=6-10, females n=9-14). Each data point represents a mouse. (**G**) Clasping scores in WT and HD mice with ATB and ATB/FMT interventions from 8-20 weeks of age (males n=8-19, females n=6-14). (**H**) Brain weight (g) at 20 weeks of age for WT and HD mice with ATB and ATB/FMT interventions (males n=8-10, females n=6-8). Each data point represents a mouse. Data and the line graphs represent mean ± SEM. Panels B-E, LMM with time as fixed effect; panels F and H, LMM followed by *post-hoc* pairwise comparisons (emmeans) *p<0.05, **p<0.01; panel G, cumulative linear mixed model with Laplace approximation. HD, Huntington's Disease; WT, wild-type; ATB, antibiotics; FMT, fecal microbiota transplant; GTT, gut transit time; LMM, linear mixed model.

Figure 2 Effects of ATB and ATB/FMT on the cognitive measures in WT and HD mice. (**A**) Diagrammatic representation of the Y-Maze trial and testing paradigms. (**B**) Time spent in each of the Y-maze arms during the second trial in WT and HD mice with ATB and ATB/FMT interventions at 12 weeks of age. Each data point represents a mouse. (**C**) Diagrammatic representation of the contextual fear conditioning and extinction paradigm. (**D**) Freeze percentage during fear conditioning in WT and HD mice with ATB and ATB/FMT interventions (each data entry represents a mouse: males n=11-15, females n=10-13). (**E**) Percentage of freezing during fear extinction in WT and HD mice with ATB and ATB/FMT interventions (each data entry represents a mouse: males n=7-16, females n=10-13). Data and the line graphs represent mean ± SEM. Panel B, LMM followed by *post-hoc* pairwise

For Review Only

comparisons (emmeans) *p<0.05, **p<0.01; panels D and E, LMM with time as fixed effect. HD, Huntington's Disease; WT, wild-type; ATB, antibiotics; FMT, fecal microbiota transplant; LMM, linear mixed model.

Figure 3 Effects of ATB and ATB/FMT on gastrointestinal measures in WT and HD mice at 14 and 20 weeks of age. (**A**) Fecal output as pellet per hour in WT and HD mice with ATB and ATB/FMT interventions from 8 to 20 weeks of age (males n=8-10, females n=6-8). (**B**) Percentage fecal water content in WT and HD mice with ATB and ATB/FMT interventions from 9 to 20 weeks of age (males n=7-11, females n=5-8). (C) Gut permeability at 20 weeks of age measure using FITC-Dextran (µg/ml) in WT and HD mice with ATB and ATB/FMT interventions (n=4-6 for both males and females). (**D**) Characterisation of macroscopy measures of colon and caecum length (cm). (**E**) Colon length (cm) in WT and HD mice with ATB and ATB/FMT interventions at 20 weeks of age (males n=7-10, females n=6). (**F**) Cecum weight in (g) in WT and HD mice with ATB and ATB/FMT interventions at 14 weeks of age (males n=5-8, females n=4-7). (**G**) Cecum length (cm) at 14 weeks of age for WT and HD mice with ATB and ATB/FMT interventions (males n=5-8, females n=4-7). Each data point represents a mouse. Data and the line graphs represent mean ± SEM. Panels A and B, LMM with time as fixed effect; panels C, E, F and G, LMM followed by *post-hoc* pairwise comparisons (emmeans) *p<0.05, **p<0.01; HD, Huntington's Disease; WT, wild-type; ATB, antibiotics; FMT, fecal microbiota transplant; LMM, linear mixed model.

Figure 4 Microbiome profiling reveals changes in gut microbiota over time. Changes in (**A**) fecal total bacterial load, (**B**) microbial richness (observed species) and (**C**) microbial diversity (Faith's phylogenetic diversity) between week 12 and week 20 across WT (each data entry represents a mouse: males n=8, females n=8) and HD groups (each data entry represents a mouse: males n=8, females n=7). The line graph represents the median and the error bars represent the interquartile ranges. Statistical analysis was performed using the Kruskal-Wallis test, followed by *post-hoc* analysis using the Dunn's test with false discovery rate (FDR) correction on multiple comparisons. Significance was determined based on FDR p<0.05, as denoted by the asterisk. Legend for statistical comparisons (*): Total bacterial load, W12: WT/H2O vs WT/ATB, FDR p=0.039; Observed species, W12: WT/H2O vs WT/ATB, FDR p=0.005. HD, Huntington's Disease; WT, wild-type; ATB, antibiotics; FMT, fecal microbiota transplant.

Figure 5 Microbiota composition of WT and HD groups at week 12 and 20. (**A**) Non-metric multi-dimensional scaling plot depicting the ordination of samples of WT or HD mice receiving the vehicle, ATB or ATB/FMT. Compositional distances between samples were determined based on weighted Unifrac distance scores. The solid and dotted lines connect the samples to the group centroid for the WT and HD groups respectively, and the colour denotes the intervention groups. (**B**) Compositional differences of ATB and ATB/FMT groups compared to the untreated WT mice at week 12 and at week 20. Larger distance scores indicate larger compositional difference to the WT receiving the vehicle. The line graph represents the median and the error bars represent the interquartile ranges. Sample numbers: Week 12 males (vehicle n=8 (WT), n=8 (HD); ATB n=9 (WT), n=10 (HD); ATB/FMT n=9 (WT), n=10 (HD)), week 20 males (vehicle n=8 (WT), n=8 (HD); ATB n=8 (WT), n=10 (HD); ATB/FMT n=9 (WT), n=10 (HD)), week 12 females (vehicle n=8 (WT), n=8 (HD); ATB n=8 (WT), n=8 (HD); ATB/FMT n=8 (WT), n=8 (HD)), week 20 females (vehicle n=6 (WT), n=8 (HD); ATB n=8 (WT), n=7 (HD); ATB/FMT n=6 (WT), n=6 (HD)). Statistical comparison between groups was performed using a permutational ANOVA (PERMANOVA), with significance determined based on $p<0.05$ as denoted by the asterisk. Each data entry represents a mouse. Legend for statistical comparisons (*): W12: WT/H2O vs WT/ATB, PERMANOVA P=0.004; W12: WT/ATB vs WT/FMT, PERMANOVA p=0.0006. HD, Huntington's disease; WT, wild-type; ATB, antibiotics; FMT, fecal microbiota transplant.

Figure 6 Effects of ATB and ATB/FMT on SCFA concentrations in WT and HD mice at 14 and 20 weeks of age. Acetate concentrations (μM) in WT and HD mice with ATB and ATB/FMT interventions at (**A**) 14 weeks of age (males n=2-8, females n=3-6) and (B) at 20 weeks of age (males n=4-6, females n=5-6). (**B**) Propionate concentrations (μM) in WT and HD mice with ATB and ATB/FMT interventions at 20 weeks of age (males n=3-6, females n=3-6). Each data point represents a mouse. Data represent mean ± SEM. Fig. 6A-C LMM. SCFAs, short-chain fatty acids; HD, Huntington's disease; WT, wild-type; ATB, antibiotics; FMT, fecal microbiota transplant; LMM, linear mixed model.

Figure 7 Effects of ATB and ATB/FMT on gut inflammation in WT and HD mice at 14 and 20 weeks of age. IFNγ (pg/mg protein) in WT and HD mice with ATB and ATB/FMT interventions at (**A**) 14 weeks of age (males n=5-7, females n=4-8) and (**B**) at 20 weeks of age (males n=4-7, females n=5-6). (**C**) IL6 (pg/mg protein) in WT and HD mice with ATB and ATB/FMT interventions at 14 weeks of age (males n=4-7, females n=4-8). (**D**) IL17E (pg/mg

For Review Only

protein) in WT and HD mice with ATB and ATB/FMT interventions at 20 weeks of age (males n=2-7, females n=3-6). (E) IL7R (pg/mg protein) in WT and HD mice with ATB and ATB/FMT interventions at 14 weeks of age (males n=2-6, females n=4-8). Each data point represents a mouse. Data represent mean ± SEM. Fig. 7A-E LMM followed by *post-hoc* pairwise comparisons (emmeans) *$p<0.05$. HD, Huntington's disease; WT, wild-type; ATB, antibiotics; FMT, fecal microbiota transplant; LMM, linear mixed model.

For Review Only

Supplementary figure legends

Supplementary Fig. 1 Effects of ATB and ATB/FMT on gastrointestinal measures in WT and HD mice at 14 and 20 weeks of age. Gut transit time (min) in WT and HD mice with ATB and ATB/FMT interventions at (**A**) 14 weeks of age (males n=7-8, females n=4-7) and (**B**) at 20 weeks of age (males n=7-8, females n=6-8). (**C**) FITC-Dextran (µg/mL) concentrations in WT and HD mice with ATB and ATB/FMT interventions at 14 weeks of age (males n=5-8, females n=4-6). (**D**) Colon length (cm) in WT and HD mice with ATB and ATB/FMT interventions at 14 weeks of age (males n=5-8, females n=4-7). (**E**) Cecum weight (g) in WT and HD mice with ATB and ATB/FMT interventions at 20 weeks of age (males n=7-10, females n=6). (**F**) Cecum length (cm) in WT and HD mice with ATB and ATB/FMT interventions at 20 weeks of age (males n=8-10, females n=6-7). Each data point represents a mouse. Data represent mean ± SEM. Fig. S1A-F LMM. HD, Huntington's disease; WT, wild-type; ATB, antibiotics; FMT, fecal microbiota transplant; LMM, linear mixed model.

Supplementary Fig. 2 Comparison of the gut microbiota of WT and HD mice in the early stages of disease at week 8. (**A**) Fecal bacterial load determined based on quantitative PCR of the 16S rRNA gene, (**B**) microbial richness based on observed species and (**C**) microbial diversity based on Faith's phylogenetic diversity between WT (each data point represents a mouse: males n=26, females n=8) and HD (each data point represents a mouse: males n=31, females n=7) males and females at week 8. (D) Non-metric multi-dimensional scaling plot of WT and HD male and female mice samples ordinated based on weighted Unifrac distances at week 8. The solid and dotted lines denote the connection of WT and HD samples to the respective group centroid. HD, Huntington's disease; WT, wild-type; ATB, antibiotics; FMT, fecal microbiota transplant; PCR, polymerase chain reaction.

Supplementary Fig. 3 Within-group weighted Unifrac distance to assess the variation in microbiota composition within WT and HD mice at week 8 (males n=8, females n=11). Microbiota composition was computed based on the weighted Unifrac distances. Higher weighted Unifrac distances denote larger within-group microbiota variation. Each data point represents the compositional distance score between two mice. HD, Huntington's Disease; WT, wild-type.

For Review Only

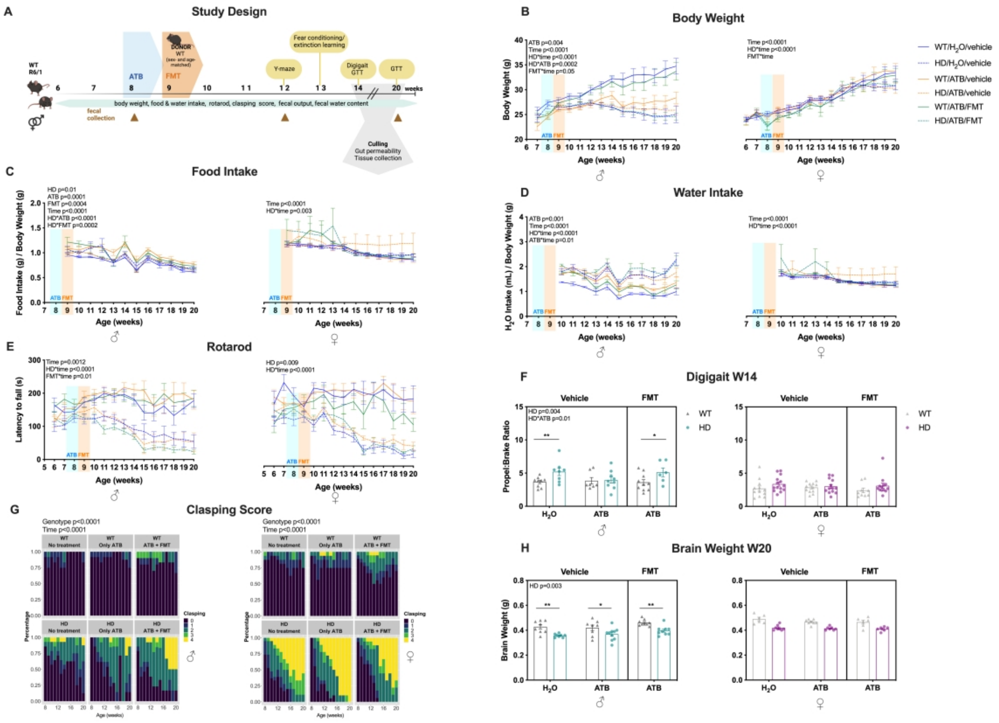

Figure 1

408x295mm (300 x 300 DPI)

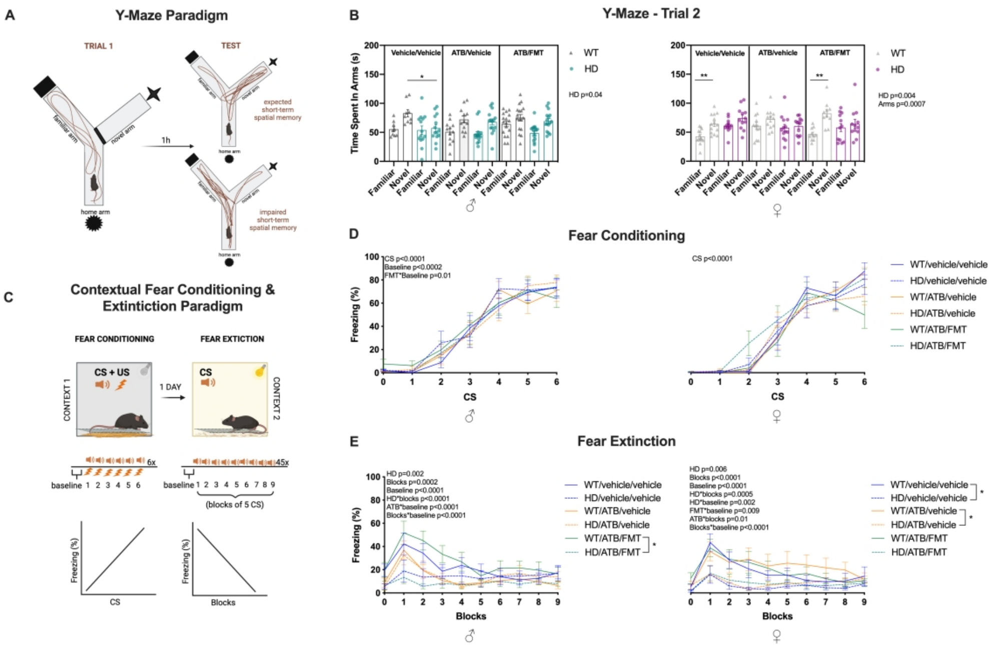

Figure 2

351x227mm (300 x 300 DPI)

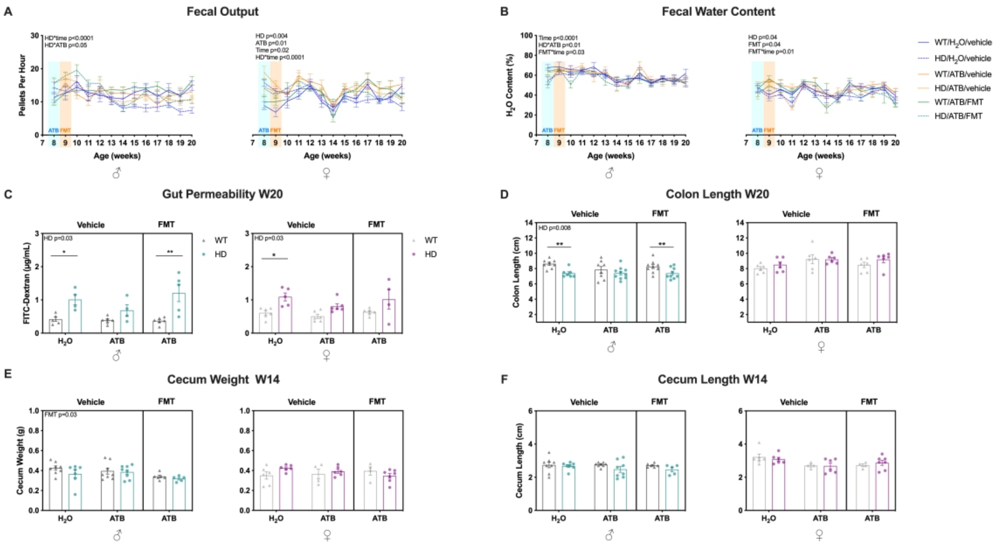

Figure 3

408x223mm (300 x 300 DPI)

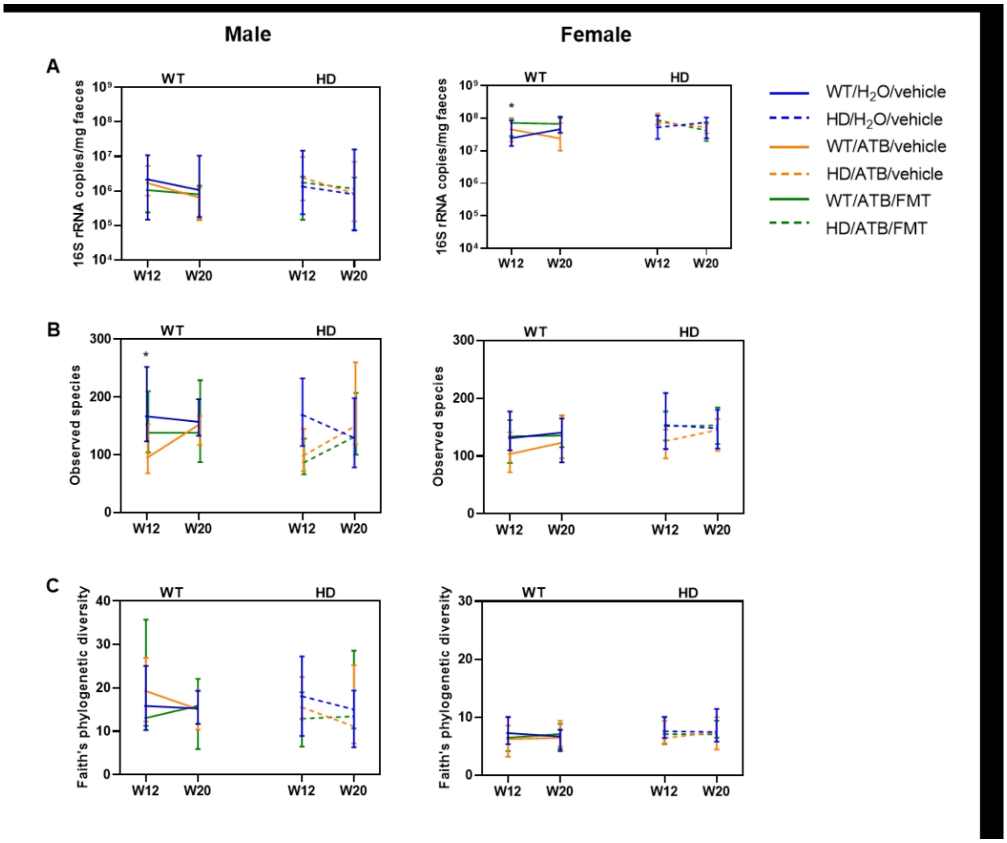

Figure 4

513x428mm (59 x 59 DPI)

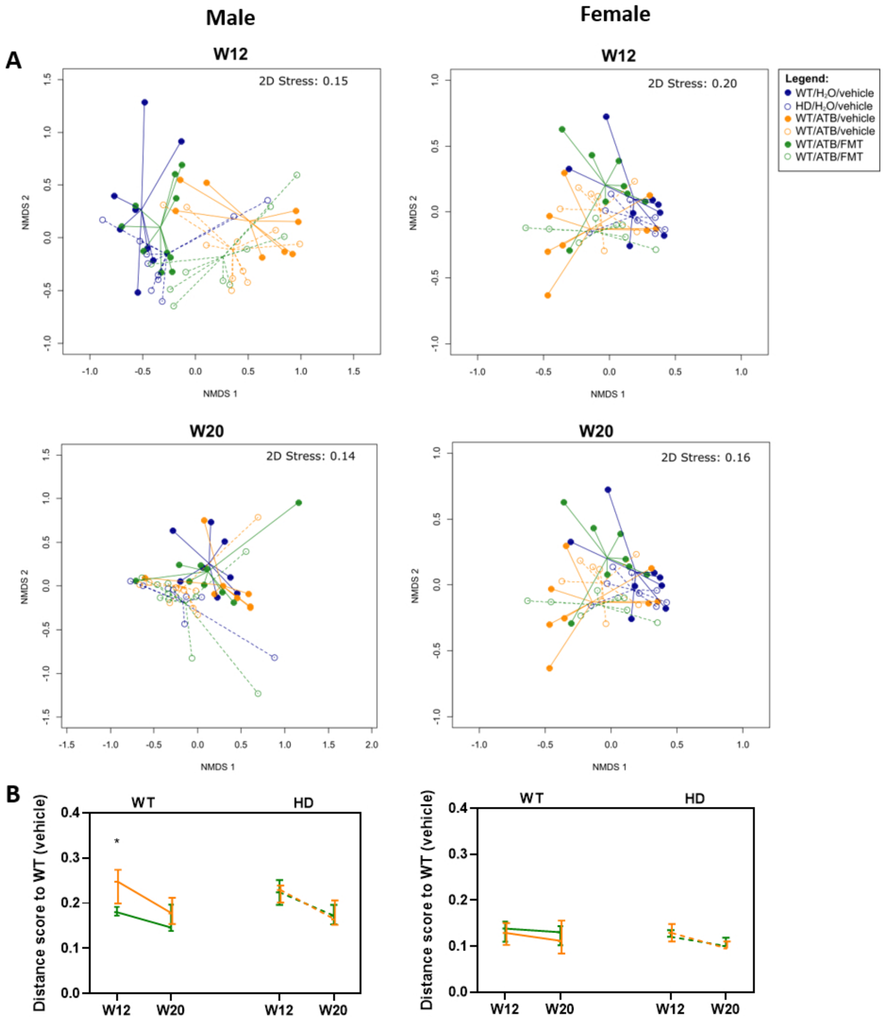

Figure 5

456x481mm (59 x 59 DPI)

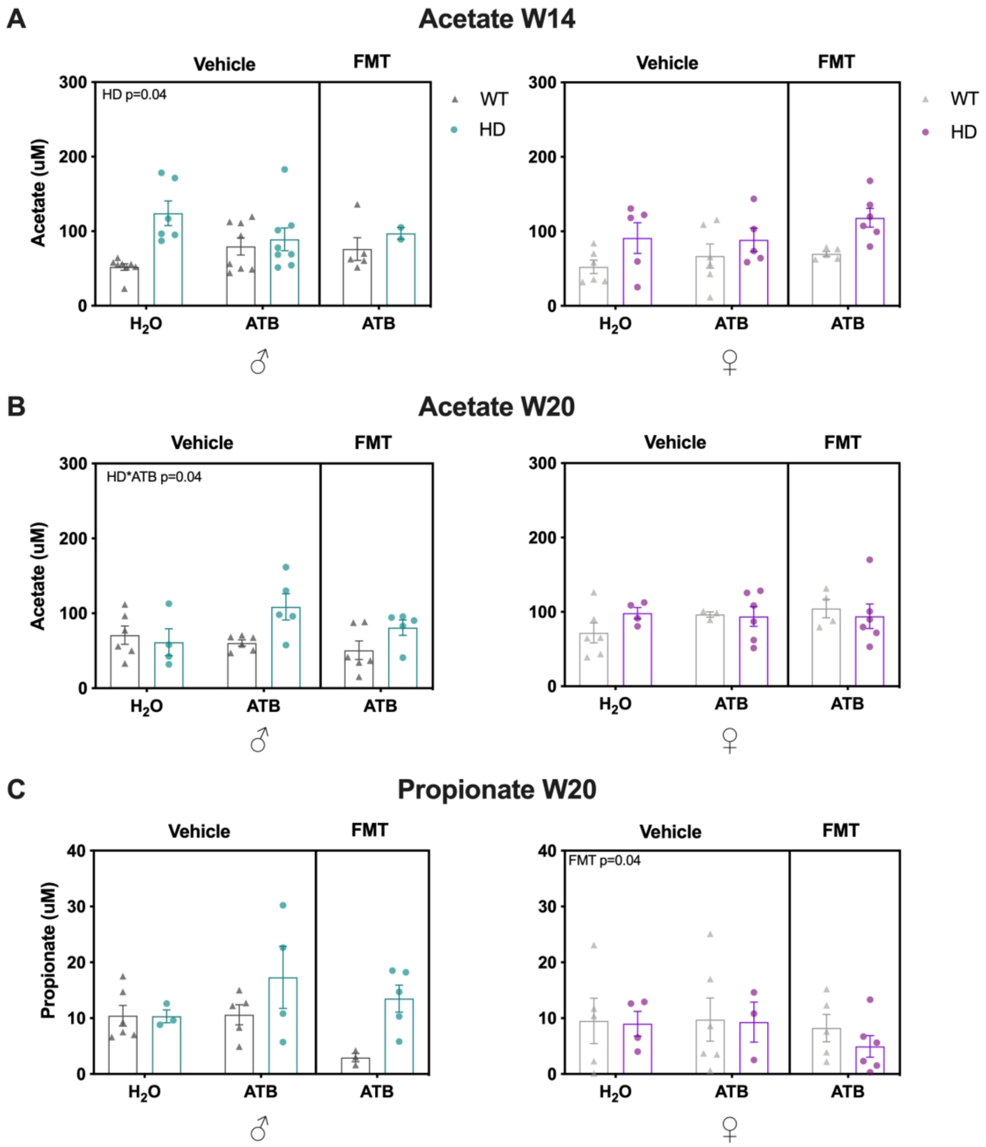

Figure 6

182x211mm (300 x 300 DPI)

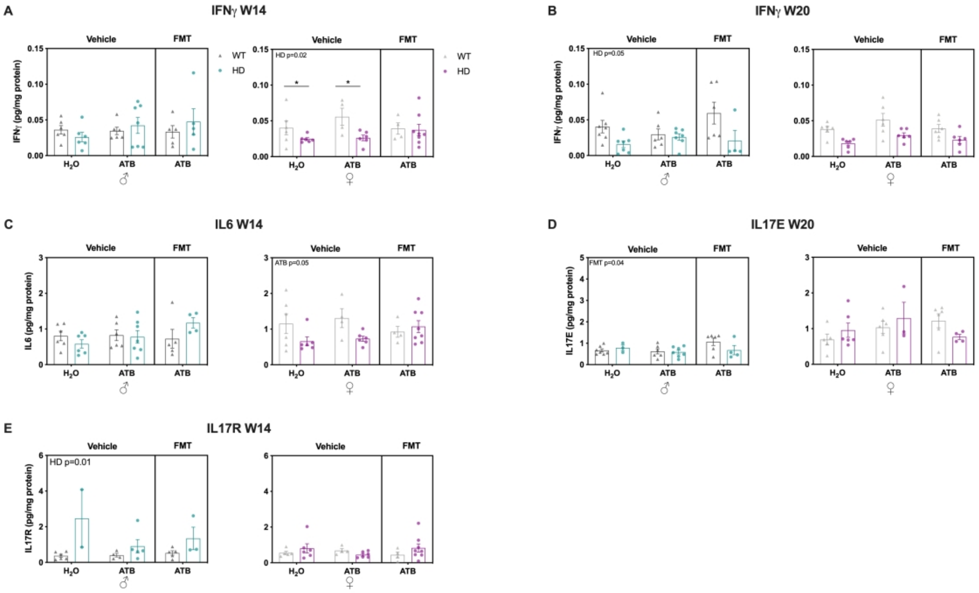

Figure 7

380x226mm (300 x 300 DPI)

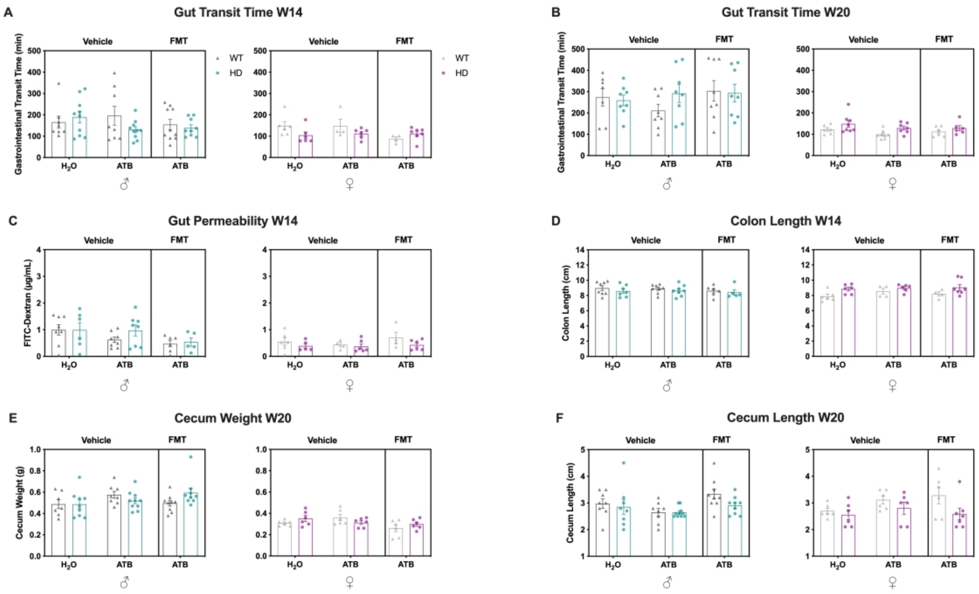

381x226mm (300 x 300 DPI)

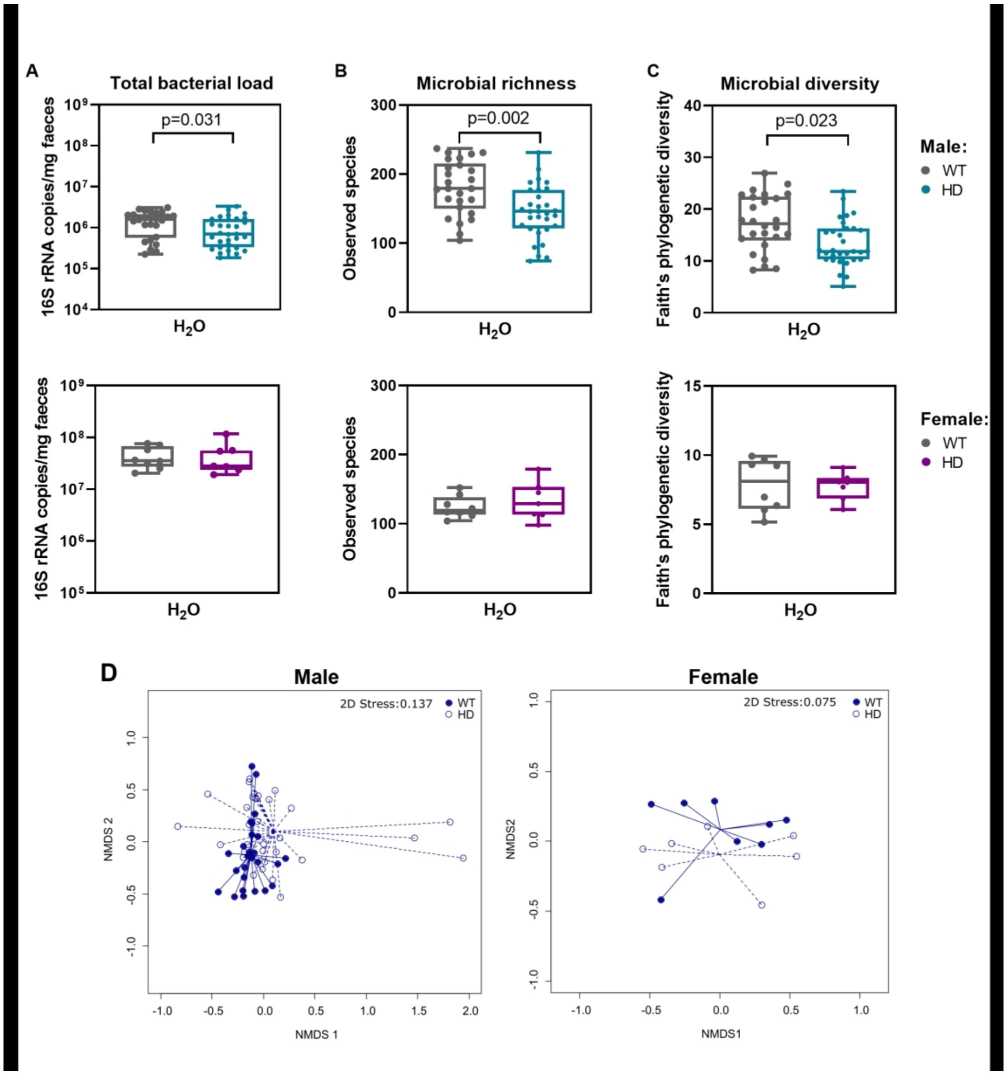

436x465mm (130 x 130 DPI)

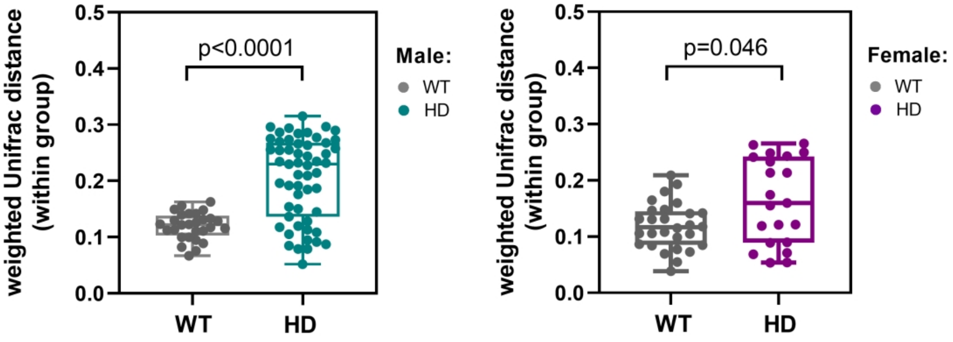

597x248mm (118 x 118 DPI)

Table S1: Post-hoc results for genotype and arm given Treatment in Ymaze in female

contrast	Treatment	estimate	SE	df	t.ratio	p.value
WT Novel - HD Novel	No treatment	18.6328	6.1519	32	3.0288	**0.0237**
WT Novel - WT Familiar	No treatment	22.3397	6.2664	69	3.5650	**0.0036**
WT Novel - HD Familiar	No treatment	32.2559	7.4644	32	4.3213	**0.0008**
HD Novel - WT Familiar	No treatment	3.7069	7.4644	32	0.4966	0.9593
HD Novel - HD Familiar	No treatment	13.6231	6.0176	69	2.2639	0.1167
WT Familiar - HD Familiar	No treatment	9.9163	6.1519	32	1.6119	0.3865
WT Novel - HD Novel	Only ATB	12.1654	6.2319	32	1.9521	0.2273
WT Novel - WT Familiar	Only ATB	11.4686	6.2929	69	1.8225	0.2717
WT Novel - HD Familiar	Only ATB	14.9175	7.6010	32	1.9626	0.2232
HD Novel - WT Familiar	Only ATB	-0.6968	7.6010	32	-0.0917	0.9997
HD Novel - HD Familiar	Only ATB	2.7521	6.1654	69	0.4464	0.9701
WT Familiar - HD Familiar	Only ATB	3.4489	6.2319	32	0.5534	0.9449
WT Novel - HD Novel	ATB_FMT	7.6466	6.5573	32	1.1661	0.6521
WT Novel - WT Familiar	ATB_FMT	24.4781	6.6793	69	3.6647	**0.0027**
WT Novel - HD Familiar	ATB_FMT	23.4082	8.1203	32	2.8827	**0.0336**
HD Novel - WT Familiar	ATB_FMT	16.8315	8.1203	32	2.0728	0.1837
HD Novel - HD Familiar	ATB_FMT	15.7616	6.4032	69	2.4615	0.0752
WT Familiar - HD Familiar	ATB_FMT	-1.0699	6.5573	32	-0.1632	0.9984

For Review Only

Table S2: Post-hoc results for genotype given Treatment in CFC Extinction in male

contrast	Treatment	estimate	SE	df	t.ratio	p.value
WT - HD	No treatment	0.8993	4.0707	41	0.2209	0.8263
WT - HD	Only ATB	1.6746	4.0536	41	0.4131	0.6817
WT - HD	ATB_FMT	10.5431	4.2334	41	2.4905	**0.0169**

Table S3: Post-hoc results for genotype given Treatment in CFC Extinction in female

contrast	Treatment	estimate	SE	df	t.ratio	p.value
WT - HD	No treatment	9.8921	4.3878	31	2.2544	**0.0314**
WT - HD	Only ATB	14.8152	4.2791	31	3.4622	**0.0016**
WT - HD	ATB_FMT	6.4316	4.8933	31	1.3144	0.1984

Table S4: Post-hoc results for genotype given Treatment in Rotarod in female

contrast	Treatment	estimate	SE	df	t.ratio	p.value
WT - HD	No treatment	119.7505	17.4210	32	6.8739	**0e+00**
WT - HD	Only ATB	99.4652	17.3779	32	5.7237	**0e+00**
WT - HD	ATB_FMT	70.4652	18.4903	32	3.8109	**6e-04**

Table S5: Post-hoc results for genotype * Treatment in Fecal Water Content in female

contrast	estimate	SE	df	t.ratio	p.value
WT No treatment - HD No treatment	4.1612	2.0162	15	2.0639	0.3548
WT No treatment - WT Only ATB	-2.9555	2.0162	15	-1.4659	0.6892
WT No treatment - HD Only ATB	1.4598	2.0630	15	0.7076	0.9781
WT No treatment - WT ATB_FMT	-1.5672	2.1601	15	-0.7255	0.9756
WT No treatment - HD ATB_FMT	3.9833	2.1600	15	1.8441	0.4693
HD No treatment - WT Only ATB	-7.1167	1.8666	15	-3.8126	**0.0173**
HD No treatment - HD Only ATB	-2.7014	1.9171	15	-1.4091	0.7215
HD No treatment - WT ATB_FMT	-5.7284	2.0212	15	-2.8341	0.1055
HD No treatment - HD ATB_FMT	-0.1779	2.0211	15	-0.0880	1.0000
WT Only ATB - HD Only ATB	4.4153	1.9171	15	2.3031	0.2518
WT Only ATB - WT ATB_FMT	1.3883	2.0212	15	0.6869	0.9807
WT Only ATB - HD ATB_FMT	6.9388	2.0211	15	3.4331	**0.0355**
HD Only ATB - WT ATB_FMT	-3.0270	2.0680	15	-1.4637	0.6904
HD Only ATB - HD ATB_FMT	2.5235	2.0679	15	1.2203	0.8206
WT ATB_FMT - HD ATB_FMT	5.5505	2.1647	15	2.5641	0.1668

For Review Only

Table S6: Post-hoc results Post-hoc results for genotype*Treatment given Time in Fecal Output in female

contrast	Time	estimate	SE	df	t.ratio	p.value
WT No treatment - HD No treatment	1	-4.0863	1.3321	15	-3.0675	0.0697
WT No treatment - WT Only ATB	1	-3.6540	1.3785	15	-2.6508	0.1445
WT No treatment - HD Only ATB	1	-6.3756	1.5162	15	-4.2050	**0.0081**
WT No treatment - WT ATB_FMT	1	-0.5487	1.4697	15	-0.3734	0.9988
WT No treatment - HD ATB_FMT	1	-4.6543	1.5832	15	-2.9397	0.0876
HD No treatment - WT Only ATB	1	0.4323	1.3892	15	0.3112	0.9995
HD No treatment - HD Only ATB	1	-2.2894	1.3122	15	-1.7447	0.5258
HD No treatment - WT ATB_FMT	1	3.5375	1.4865	15	2.3797	0.2239
HD No treatment - HD ATB_FMT	1	-0.5680	1.3963	15	-0.4068	0.9983
WT Only ATB - HD Only ATB	1	-2.7216	1.2634	15	-2.1543	0.3130
WT Only ATB - WT ATB_FMT	1	3.1053	1.3906	15	2.2330	0.2794
WT Only ATB - HD ATB_FMT	1	-1.0003	1.4890	15	-0.6718	0.9825
HD Only ATB - WT ATB_FMT	1	5.8269	1.5130	15	3.8511	**0.0160**
HD Only ATB - HD ATB_FMT	1	1.7214	1.4020	15	1.2278	0.8170
WT ATB_FMT - HD ATB_FMT	1	-4.1055	1.4088	15	-2.9142	0.0917
WT No treatment - HD No treatment	2	-3.4982	1.2968	15	-2.6975	0.1335
WT No treatment - WT Only ATB	2	-3.5466	1.3301	15	-2.6665	0.1407
WT No treatment - HD Only ATB	2	-5.6801	1.4338	15	-3.9617	**0.0130**
WT No treatment - WT ATB_FMT	2	-0.6496	1.4190	15	-0.4578	0.9970
WT No treatment - HD ATB_FMT	2	-4.1670	1.5015	15	-2.7753	0.1169
HD No treatment - WT Only ATB	2	-0.0484	1.3136	15	-0.0369	1.0000
HD No treatment - HD Only ATB	2	-2.1820	1.2612	15	-1.7300	0.5343
HD No treatment - WT ATB_FMT	2	2.8486	1.4086	15	2.0223	0.3751
HD No treatment - HD ATB_FMT	2	-0.6688	1.3430	15	-0.4980	0.9955
WT Only ATB - HD Only ATB	2	-2.1335	1.2261	15	-1.7400	0.5285
WT Only ATB - WT ATB_FMT	2	2.8970	1.3388	15	2.1638	0.3088
WT Only ATB - HD ATB_FMT	2	-0.6204	1.4104	15	-0.4399	0.9975

Table S6: Post-hoc results Post-hoc results for genotype*Treatment given Time in Fecal Output in female *(continued)*

contrast	Time	estimate	SE	df	t.ratio	p.value
HD Only ATB - WT ATB_FMT	2	5.0305	1.4314	15	3.5143	**0.0304**
HD Only ATB - HD ATB_FMT	2	1.5131	1.3507	15	1.1203	0.8656
WT ATB_FMT - HD ATB_FMT	2	-3.5174	1.3755	15	-2.5572	0.1687
WT No treatment - HD No treatment	3	-2.9101	1.2673	15	-2.2963	0.2543
WT No treatment - WT Only ATB	3	-3.4392	1.2891	15	-2.6679	0.1404
WT No treatment - HD Only ATB	3	-4.9846	1.3626	15	-3.6582	**0.0232**
WT No treatment - WT ATB_FMT	3	-0.7504	1.3762	15	-0.5453	0.9931
WT No treatment - HD ATB_FMT	3	-3.6798	1.4311	15	-2.5712	0.1648
HD No treatment - WT Only ATB	3	-0.5291	1.2483	15	-0.4239	0.9979
HD No treatment - HD Only ATB	3	-2.0746	1.2180	15	-1.7033	0.5499
HD No treatment - WT ATB_FMT	3	2.1596	1.3414	15	1.6100	0.6048
HD No treatment - HD ATB_FMT	3	-0.7697	1.2976	15	-0.5931	0.9900
WT Only ATB - HD Only ATB	3	-1.5454	1.1948	15	-1.2935	0.7841
WT Only ATB - WT ATB_FMT	3	2.6888	1.2949	15	2.0764	0.3488
WT Only ATB - HD ATB_FMT	3	-0.2406	1.3426	15	-0.1792	1.0000
HD Only ATB - WT ATB_FMT	3	4.2342	1.3610	15	3.1110	0.0644
HD Only ATB - HD ATB_FMT	3	1.3049	1.3071	15	0.9983	0.9114
WT ATB_FMT - HD ATB_FMT	3	-2.9293	1.3476	15	-2.1737	0.3045
WT No treatment - HD No treatment	4	-2.3220	1.2438	15	-1.8669	0.4567
WT No treatment - WT Only ATB	4	-3.3318	1.2563	15	-2.6520	0.1442
WT No treatment - HD Only ATB	4	-4.2892	1.3046	15	-3.2878	**0.0465**
WT No treatment - WT ATB_FMT	4	-0.8513	1.3420	15	-0.6343	0.9864
WT No treatment - HD ATB_FMT	4	-3.1925	1.3739	15	-2.3237	0.2440
HD No treatment - WT Only ATB	4	-1.0098	1.1951	15	-0.8450	0.9539
HD No treatment - HD Only ATB	4	-1.9672	1.1832	15	-1.6625	0.5738
HD No treatment - WT ATB_FMT	4	1.4707	1.2868	15	1.1429	0.8559
HD No treatment - HD ATB_FMT	4	-0.8705	1.2613	15	-0.6902	0.9803
WT Only ATB - HD Only ATB	4	-0.9573	1.1699	15	-0.8183	0.9595
WT Only ATB - WT ATB_FMT	4	2.4805	1.2597	15	1.9691	0.4021
WT Only ATB - HD ATB_FMT	4	0.1393	1.2875	15	0.1082	1.0000
HD Only ATB - WT ATB_FMT	4	3.4379	1.3037	15	2.6371	0.1478
HD Only ATB - HD ATB_FMT	4	1.0966	1.2722	15	0.8620	0.9500
WT ATB_FMT - HD ATB_FMT	4	-2.3412	1.3256	15	-1.7662	0.5134
WT No treatment - HD No treatment	5	-1.7339	1.2267	15	-1.4134	0.7191
WT No treatment - WT Only ATB	5	-3.2244	1.2324	15	-2.6164	0.1530
WT No treatment - HD Only ATB	5	-3.5937	1.2615	15	-2.8487	0.1029
WT No treatment - WT ATB_FMT	5	-0.9521	1.3170	15	-0.7230	0.9760
WT No treatment - HD ATB_FMT	5	-2.7053	1.3315	15	-2.0317	0.3705
HD No treatment - WT Only ATB	5	-1.4905	1.1555	15	-1.2899	0.7860
HD No treatment - HD Only ATB	5	-1.8598	1.1578	15	-1.6063	0.6069
HD No treatment - WT ATB_FMT	5	0.7817	1.2463	15	0.6273	0.9871
HD No treatment - HD ATB_FMT	5	-0.9714	1.2346	15	-0.7868	0.9656
WT Only ATB - HD Only ATB	5	-0.3692	1.1517	15	-0.3206	0.9994
WT Only ATB - WT ATB_FMT	5	2.2723	1.2339	15	1.8415	0.4707
WT Only ATB - HD ATB_FMT	5	0.5191	1.2466	15	0.4165	0.9981
HD Only ATB - WT ATB_FMT	5	2.6415	1.2611	15	2.0947	0.3402
HD Only ATB - HD ATB_FMT	5	0.8884	1.2467	15	0.7126	0.9774
WT ATB_FMT - HD ATB_FMT	5	-1.7531	1.3096	15	-1.3387	0.7603
WT No treatment - HD No treatment	6	-1.1458	1.2164	15	-0.9420	0.9290

For Review Only

Table S6: Post-hoc results Post-hoc results for genotype*Treatment given Time in Fecal Output in female *(continued)*

contrast	Time	estimate	SE	df	t.ratio	p.value
WT No treatment - WT Only ATB	6	-3.1170	1.2178	15	-2.5595	0.1680
WT No treatment - HD Only ATB	6	-2.8982	1.2349	15	-2.3468	0.2355
WT No treatment - WT ATB_FMT	6	-1.0530	1.3018	15	-0.8089	0.9614
WT No treatment - HD ATB_FMT	6	-2.2180	1.3055	15	-1.6990	0.5524
HD No treatment - WT Only ATB	6	-1.9712	1.1312	15	-1.7426	0.5270
HD No treatment - HD Only ATB	6	-1.7524	1.1422	15	-1.5342	0.6495
HD No treatment - WT ATB_FMT	6	0.0928	1.2213	15	0.0760	1.0000
HD No treatment - HD ATB_FMT	6	-1.0722	1.2184	15	-0.8800	0.9456
WT Only ATB - HD Only ATB	6	0.2188	1.1407	15	0.1919	1.0000
WT Only ATB - WT ATB_FMT	6	2.0640	1.2182	15	1.6943	0.5551
WT Only ATB - HD ATB_FMT	6	0.8990	1.2214	15	0.7360	0.9740
HD Only ATB - WT ATB_FMT	6	1.8452	1.2348	15	1.4943	0.6728
HD Only ATB - HD ATB_FMT	6	0.6801	1.2312	15	0.5524	0.9927
WT ATB_FMT - HD ATB_FMT	6	-1.1650	1.2999	15	-0.8962	0.9416
WT No treatment - HD No treatment	7	-0.5577	1.2129	15	-0.4598	0.9969
WT No treatment - WT Only ATB	7	-3.0096	1.2129	15	-2.4813	0.1908
WT No treatment - HD Only ATB	7	-2.2027	1.2259	15	-1.7967	0.4959
WT No treatment - WT ATB_FMT	7	-1.1538	1.2966	15	-0.8899	0.9432
WT No treatment - HD ATB_FMT	7	-1.7308	1.2966	15	-1.3348	0.7623
HD No treatment - WT Only ATB	7	-2.4519	1.1229	15	-2.1835	0.3002
HD No treatment - HD Only ATB	7	-1.6450	1.1370	15	-1.4468	0.7002
HD No treatment - WT ATB_FMT	7	-0.5962	1.2129	15	-0.4915	0.9958
HD No treatment - HD ATB_FMT	7	-1.1731	1.2129	15	-0.9672	0.9214
WT Only ATB - HD Only ATB	7	0.8069	1.1370	15	0.7097	0.9778
WT Only ATB - WT ATB_FMT	7	1.8558	1.2129	15	1.5300	0.6519
WT Only ATB - HD ATB_FMT	7	1.2788	1.2129	15	1.0544	0.8916
HD Only ATB - WT ATB_FMT	7	1.0488	1.2259	15	0.8555	0.9515
HD Only ATB - HD ATB_FMT	7	0.4719	1.2259	15	0.3849	0.9987
WT ATB_FMT - HD ATB_FMT	7	-0.5769	1.2966	15	-0.4449	0.9973
WT No treatment - HD No treatment	8	0.0304	1.2164	15	0.0250	1.0000
WT No treatment - WT Only ATB	8	-2.9022	1.2178	15	-2.3832	0.2227
WT No treatment - HD Only ATB	8	-1.5072	1.2349	15	-1.2205	0.8205
WT No treatment - WT ATB_FMT	8	-1.2547	1.3018	15	-0.9638	0.9224
WT No treatment - HD ATB_FMT	8	-1.2435	1.3055	15	-0.9526	0.9259
HD No treatment - WT Only ATB	8	-2.9326	1.1312	15	-2.5925	0.1592
HD No treatment - HD Only ATB	8	-1.5376	1.1422	15	-1.3461	0.7563
HD No treatment - WT ATB_FMT	8	-1.2851	1.2213	15	-1.0522	0.8925
HD No treatment - HD ATB_FMT	8	-1.2739	1.2184	15	-1.0456	0.8949
WT Only ATB - HD Only ATB	8	1.3950	1.1407	15	1.2230	0.8193
WT Only ATB - WT ATB_FMT	8	1.6475	1.2182	15	1.3524	0.7528
WT Only ATB - HD ATB_FMT	8	1.6587	1.2214	15	1.3580	0.7498
HD Only ATB - WT ATB_FMT	8	0.2525	1.2348	15	0.2045	0.9999
HD Only ATB - HD ATB_FMT	8	0.2637	1.2312	15	0.2141	0.9999
WT ATB_FMT - HD ATB_FMT	8	0.0112	1.2999	15	0.0086	1.0000
WT No treatment - HD No treatment	9	0.6185	1.2267	15	0.5042	0.9952
WT No treatment - WT Only ATB	9	-2.7948	1.2324	15	-2.2678	0.2654
WT No treatment - HD Only ATB	9	-0.8117	1.2615	15	-0.6434	0.9855
WT No treatment - WT ATB_FMT	9	-1.3555	1.3170	15	-1.0293	0.9008
WT No treatment - HD ATB_FMT	9	-0.7563	1.3315	15	-0.5680	0.9917

For Review Only

Table S6: Post-hoc results Post-hoc results for genotype*Treatment given Time in Fecal Output in female *(continued)*

contrast	Time	estimate	SE	df	t.ratio	p.value
HD No treatment - WT Only ATB	9	-3.4133	1.1555	15	-2.9539	0.0855
HD No treatment - HD Only ATB	9	-1.4302	1.1578	15	-1.2353	0.8134
HD No treatment - WT ATB_FMT	9	-1.9740	1.2463	15	-1.5840	0.6202
HD No treatment - HD ATB_FMT	9	-1.3748	1.2346	15	-1.1135	0.8684
WT Only ATB - HD Only ATB	9	1.9831	1.1517	15	1.7219	0.5390
WT Only ATB - WT ATB_FMT	9	1.4393	1.2339	15	1.1664	0.8456
WT Only ATB - HD ATB_FMT	9	2.0385	1.2466	15	1.6353	0.5898
HD Only ATB - WT ATB_FMT	9	-0.5439	1.2611	15	-0.4313	0.9977
HD Only ATB - HD ATB_FMT	9	0.0554	1.2467	15	0.0444	1.0000
WT ATB_FMT - HD ATB_FMT	9	0.5993	1.3096	15	0.4576	0.9970
WT No treatment - HD No treatment	10	1.2066	1.2438	15	0.9701	0.9205
WT No treatment - WT Only ATB	10	-2.6874	1.2563	15	-2.1391	0.3198
WT No treatment - HD Only ATB	10	-0.1162	1.3046	15	-0.0891	1.0000
WT No treatment - WT ATB_FMT	10	-1.4564	1.3420	15	-1.0852	0.8798
WT No treatment - HD ATB_FMT	10	-0.2690	1.3739	15	-0.1958	1.0000
HD No treatment - WT Only ATB	10	-3.8940	1.1951	15	-3.2584	**0.0491**
HD No treatment - HD Only ATB	10	-1.3228	1.1832	15	-1.1179	0.8665
HD No treatment - WT ATB_FMT	10	-2.6630	1.2868	15	-2.0695	0.3521
HD No treatment - HD ATB_FMT	10	-1.4756	1.2613	15	-1.1700	0.8440
WT Only ATB - HD Only ATB	10	2.5712	1.1699	15	2.1979	0.2941
WT Only ATB - WT ATB_FMT	10	1.2310	1.2597	15	0.9772	0.9183
WT Only ATB - HD ATB_FMT	10	2.4184	1.2875	15	1.8784	0.4504
HD Only ATB - WT ATB_FMT	10	-1.3402	1.3037	15	-1.0280	0.9012
HD Only ATB - HD ATB_FMT	10	-0.1528	1.2722	15	-0.1201	1.0000
WT ATB_FMT - HD ATB_FMT	10	1.1874	1.3256	15	0.8957	0.9417
WT No treatment - HD No treatment	11	1.7947	1.2673	15	1.4162	0.7175
WT No treatment - WT Only ATB	11	-2.5800	1.2891	15	-2.0014	0.3856
WT No treatment - HD Only ATB	11	0.5793	1.3626	15	0.4252	0.9979
WT No treatment - WT ATB_FMT	11	-1.5572	1.3762	15	-1.1315	0.8608
WT No treatment - HD ATB_FMT	11	0.2182	1.4311	15	0.1525	1.0000
HD No treatment - WT Only ATB	11	-4.3747	1.2483	15	-3.5046	**0.0310**
HD No treatment - HD Only ATB	11	-1.2154	1.2180	15	-0.9979	0.9116
HD No treatment - WT ATB_FMT	11	-3.3519	1.3414	15	-2.4988	0.1855
HD No treatment - HD ATB_FMT	11	-1.5765	1.2976	15	-1.2149	0.8232
WT Only ATB - HD Only ATB	11	3.1593	1.1948	15	2.6442	0.1461
WT Only ATB - WT ATB_FMT	11	1.0228	1.2949	15	0.7898	0.9650
WT Only ATB - HD ATB_FMT	11	2.7982	1.3426	15	2.0842	0.3451
HD Only ATB - WT ATB_FMT	11	-2.1366	1.3610	15	-1.5698	0.6285
HD Only ATB - HD ATB_FMT	11	-0.3611	1.3071	15	-0.2762	0.9997
WT ATB_FMT - HD ATB_FMT	11	1.7755	1.3476	15	1.3175	0.7716
WT No treatment - HD No treatment	12	2.3828	1.2968	15	1.8374	0.4730
WT No treatment - WT Only ATB	12	-2.4726	1.3301	15	-1.8590	0.4610
WT No treatment - HD Only ATB	12	1.2748	1.4338	15	0.8891	0.9434
WT No treatment - WT ATB_FMT	12	-1.6581	1.4190	15	-1.1685	0.8447
WT No treatment - HD ATB_FMT	12	0.7055	1.5015	15	0.4698	0.9966
HD No treatment - WT Only ATB	12	-4.8554	1.3136	15	-3.6964	**0.0216**
HD No treatment - HD Only ATB	12	-1.1080	1.2612	15	-0.8785	0.9460
HD No treatment - WT ATB_FMT	12	-4.0409	1.4086	15	-2.8688	0.0993
HD No treatment - HD ATB_FMT	12	-1.6773	1.3430	15	-1.2490	0.8066

For Review Only

Table S6: Post-hoc results Post-hoc results for genotype*Treatment given Time in Fecal Output in female *(continued)*

contrast	Time	estimate	SE	df	t.ratio	p.value
WT Only ATB - HD Only ATB	12	3.7474	1.2261	15	3.0563	0.0711
WT Only ATB - WT ATB_FMT	12	0.8145	1.3388	15	0.6084	0.9887
WT Only ATB - HD ATB_FMT	12	3.1781	1.4104	15	2.2534	0.2711
HD Only ATB - WT ATB_FMT	12	-2.9329	1.4314	15	-2.0489	0.3620
HD Only ATB - HD ATB_FMT	12	-0.5693	1.3507	15	-0.4215	0.9979
WT ATB_FMT - HD ATB_FMT	12	2.3636	1.3755	15	1.7183	0.5411
WT No treatment - HD No treatment	13	2.9709	1.3321	15	2.2302	0.2805
WT No treatment - WT Only ATB	13	-2.3652	1.3785	15	-1.7158	0.5425
WT No treatment - HD Only ATB	13	1.9703	1.5162	15	1.2995	0.7810
WT No treatment - WT ATB_FMT	13	-1.7589	1.4697	15	-1.1968	0.8317
WT No treatment - HD ATB_FMT	13	1.1927	1.5832	15	0.7533	0.9713
HD No treatment - WT Only ATB	13	-5.3361	1.3892	15	-3.8412	**0.0164**
HD No treatment - HD Only ATB	13	-1.0006	1.3122	15	-0.7625	0.9698
HD No treatment - WT ATB_FMT	13	-4.7298	1.4865	15	-3.1818	0.0566
HD No treatment - HD ATB_FMT	13	-1.7782	1.3963	15	-1.2735	0.7944
WT Only ATB - HD Only ATB	13	4.3355	1.2634	15	3.4317	**0.0356**
WT Only ATB - WT ATB_FMT	13	0.6063	1.3906	15	0.4360	0.9976
WT Only ATB - HD ATB_FMT	13	3.5579	1.4890	15	2.3895	0.2205
HD Only ATB - WT ATB_FMT	13	-3.7293	1.5130	15	-2.4648	0.1959
HD Only ATB - HD ATB_FMT	13	-0.7776	1.4020	15	-0.5546	0.9926
WT ATB_FMT - HD ATB_FMT	13	2.9517	1.4088	15	2.0952	0.3399

Table S7: Post-hoc results for genotype given Treatment in FITC

contrast	Treatment	estimate	SE	df	t.ratio	p.value
WT - HD	No treatment	-0.4804	0.2031	11	-2.3648	**0.0375**
WT - HD	Only ATB	-0.2980	0.1995	11	-1.4936	0.1634
WT - HD	ATB_FMT	-0.3774	0.2230	11	-1.6921	0.1187

Table S8: Post-hoc results for Treatment given genotype in Propionate

contrast	genotype	estimate	SE	df	t.ratio	p.value
No treatment - Only ATB	WT	0.0569	5.0083	9	0.0114	0.9999
No treatment - ATB_FMT	WT	-10.8384	4.5975	9	-2.3575	0.0980
Only ATB - ATB_FMT	WT	-10.8953	5.4139	9	-2.0125	0.1648
No treatment - Only ATB	HD	-6.9653	4.5975	9	-1.5150	0.3293
No treatment - ATB_FMT	HD	-4.7370	4.5975	9	-1.0303	0.5775
Only ATB - ATB_FMT	HD	2.2283	4.1121	9	0.5419	0.8530

Table S9: Post-hoc results for genotype given Treatment in IFNg

contrast	Treatment	estimate	SE	df	t.ratio	p.value
WT - HD	No treatment	0.0200	0.0076	12	2.6196	**0.0224**

For Review Only

Table S9: Post-hoc results for genotype given Treatment in IFNg *(continued)*

contrast	Treatment	estimate	SE	df	t.ratio	p.value
WT - HD	Only ATB	0.0215	0.0076	12	2.8133	**0.0157**
WT - HD	ATB_FMT	0.0158	0.0076	12	2.0701	0.0607

Table S10: Post-hoc results for genotype *Treatment in body weight in male

contrast	estimate	SE	df	t.ratio	p.value
WT No treatment - HD No treatment	5.5503	0.9342	71	5.9415	**0.0000**
WT No treatment - WT Only ATB	2.8106	1.0413	1297	2.6992	0.0760
WT No treatment - HD Only ATB	3.9178	0.9354	71	4.1886	**0.0011**
WT No treatment - WT ATB_FMT	0.5025	1.0205	71	0.4924	0.9963
WT No treatment - HD ATB_FMT	4.9871	1.0245	71	4.8679	**0.0001**
HD No treatment - WT Only ATB	-2.7396	0.9361	71	-2.9266	0.0503
HD No treatment - HD Only ATB	-1.6325	0.5282	1297	-3.0908	**0.0249**
HD No treatment - WT ATB_FMT	-5.0478	0.9141	71	-5.5224	**0.0000**
HD No treatment - HD ATB_FMT	-0.5632	0.9119	71	-0.6176	0.9894
WT Only ATB - HD Only ATB	1.1072	0.9336	71	1.1859	0.8421
WT Only ATB - WT ATB_FMT	-2.3082	1.0205	71	-2.2617	0.2236
WT Only ATB - HD ATB_FMT	2.1765	1.0245	71	2.1244	0.2869
HD Only ATB - WT ATB_FMT	-3.4153	0.9129	71	-3.7411	**0.0048**
HD Only ATB - HD ATB_FMT	1.0693	0.9113	71	1.1734	0.8480
WT ATB_FMT - HD ATB_FMT	4.4846	0.9988	71	4.4900	**0.0004**

Table S11: Post-hoc results for genotype * Treatment in Weight Gain in male

contrast	estimate	SE	df	t.ratio	p.value
WT No treatment - HD No treatment	17.6839	3.5853	23	4.9324	**0.0007**
WT No treatment - WT Only ATB	7.2766	4.0791	682	1.7839	0.4769
WT No treatment - HD Only ATB	13.3880	3.5875	23	3.7319	**0.0123**
WT No treatment - WT ATB_FMT	2.1142	3.8698	23	0.5463	0.9935
WT No treatment - HD ATB_FMT	21.0392	3.8809	23	5.4212	**0.0002**
HD No treatment - WT Only ATB	-10.4073	3.5853	23	-2.9028	0.0759
HD No treatment - HD Only ATB	-4.2958	1.8509	682	-2.3209	0.1870
HD No treatment - WT ATB_FMT	-15.5697	3.3452	23	-4.6544	**0.0014**
HD No treatment - HD ATB_FMT	3.3553	3.3580	23	0.9992	0.9135
WT Only ATB - HD Only ATB	6.1114	3.5875	23	1.7035	0.5432
WT Only ATB - WT ATB_FMT	-5.1624	3.8698	23	-1.3340	0.7637
WT Only ATB - HD ATB_FMT	13.7626	3.8809	23	3.5463	**0.0188**
HD Only ATB - WT ATB_FMT	-11.2738	3.3476	23	-3.3678	**0.0281**
HD Only ATB - HD ATB_FMT	7.6512	3.3604	23	2.2769	0.2434
WT ATB_FMT - HD ATB_FMT	18.9250	3.6602	23	5.1704	**0.0004**

For Review Only

Table S12: Post-hoc results for genotype * Treatment in Fecal Water Content in male

contrast	estimate	SE	df	t.ratio	p.value
WT No treatment - HD No treatment	15.6830	8.5175	23	1.8413	0.4608
WT No treatment - WT Only ATB	0.6866	9.7349	677	0.0705	1.0000
WT No treatment - HD Only ATB	-9.6736	8.5237	23	-1.1349	0.8618
WT No treatment - WT ATB_FMT	4.6863	9.2371	23	0.5073	0.9954
WT No treatment - HD ATB_FMT	-1.0366	9.2566	23	-0.1120	1.0000
HD No treatment - WT Only ATB	-14.9964	8.5175	23	-1.7607	0.5086
HD No treatment - HD Only ATB	-25.3566	4.1105	677	-6.1687	**0.0000**
HD No treatment - WT ATB_FMT	-10.9967	7.9437	23	-1.3843	0.7356
HD No treatment - HD ATB_FMT	-16.7196	7.9664	23	-2.0988	0.3224
WT Only ATB - HD Only ATB	-10.3603	8.5237	23	-1.2155	0.8249
WT Only ATB - WT ATB_FMT	3.9997	9.2371	23	0.4330	0.9978
WT Only ATB - HD ATB_FMT	-1.7233	9.2566	23	-0.1862	1.0000
HD Only ATB - WT ATB_FMT	14.3599	7.9504	23	1.8062	0.4814
HD Only ATB - HD ATB_FMT	8.6370	7.9730	23	1.0833	0.8830
WT ATB_FMT - HD ATB_FMT	-5.7229	8.7315	23	-0.6554	0.9851

Table S13: Post-hoc results for genotype * Treatment in Food Intake in male

contrast	estimate	SE	df	t.ratio	p.value
WT No treatment - HD No treatment	-0.0973	0.0345	22	-2.8239	0.0906
WT No treatment - WT Only ATB	-0.1906	0.0366	292	-5.2061	**0.0000**
WT No treatment - HD Only ATB	-0.0659	0.0345	22	-1.9130	0.4208
WT No treatment - WT ATB_FMT	-0.1767	0.0347	22	-5.0869	**0.0005**
WT No treatment - HD ATB_FMT	-0.0519	0.0366	22	-1.4181	0.7162
HD No treatment - WT Only ATB	-0.0933	0.0345	22	-2.7089	0.1134
HD No treatment - HD Only ATB	0.0314	0.0304	292	1.0328	0.9066
HD No treatment - WT ATB_FMT	-0.0794	0.0324	22	-2.4472	0.1835
HD No treatment - HD ATB_FMT	0.0454	0.0345	22	1.3168	0.7729
WT Only ATB - HD Only ATB	0.1247	0.0345	22	3.6199	**0.0166**
WT Only ATB - WT ATB_FMT	0.0139	0.0347	22	0.4008	0.9985
WT Only ATB - HD ATB_FMT	0.1387	0.0366	22	3.7879	**0.0113**
HD Only ATB - WT ATB_FMT	-0.1108	0.0324	22	-3.4144	**0.0262**
HD Only ATB - HD ATB_FMT	0.0140	0.0345	22	0.4058	0.9984
WT ATB_FMT - HD ATB_FMT	0.1248	0.0347	22	3.5921	**0.0177**

Table S14: Post-hoc results for Treatment given genotype in Water Intake in male

contrast	genotype	estimate	SE	df	t.ratio	p.value
No treatment - Only ATB	WT	-0.3721	0.1511	264	-2.4622	**0.0383**
No treatment - ATB_FMT	WT	-0.2737	0.1434	22	-1.9092	0.1598
Only ATB - ATB_FMT	WT	0.0984	0.1434	22	0.6862	0.7739
No treatment - Only ATB	HD	-0.2293	0.0974	264	-2.3526	0.0505
No treatment - ATB_FMT	HD	-0.3113	0.1372	22	-2.2691	0.0817
Only ATB - ATB_FMT	HD	-0.0820	0.1372	22	-0.5974	0.8229

https://mc.manuscriptcentral.com/braincom

For Review Only

Table S15: Post-hoc results for genotype given Treatment*Arm in Ymaze in male

contrast	Treatment	Arm	estimate	SE	df	t.ratio	p.value
WT - HD	No treatment	Novel	17.1598	8.2060	39	2.0911	**0.0431**
WT - HD	Only ATB	Novel	2.8606	7.7449	39	0.3694	0.7139
WT - HD	ATB_FMT	Novel	14.4345	7.5293	39	1.9171	0.0626
WT - HD	No treatment	Familiar	16.3095	8.2060	39	1.9875	0.0539
WT - HD	Only ATB	Familiar	2.0103	7.7449	39	0.2596	0.7966
WT - HD	ATB_FMT	Familiar	13.5842	7.5293	39	1.8042	0.0789

Table S16: Post-hoc results for for genotype given Treatment in Brain weight in male

contrast	Treatment	estimate	SE	df	t.ratio	p.value
WT - HD	No treatment	0.0700	0.0213	23	3.2864	**0.0032**
WT - HD	Only ATB	0.0510	0.0202	23	2.5239	**0.0190**
WT - HD	ATB_FMT	0.0644	0.0201	23	3.2091	**0.0039**

Table S17: Post-hoc results for genotype given Treatment in Propel Brake Ratio in male

contrast	Treatment	estimate	SE	df	t.ratio	p.value
WT - HD	No treatment	-1.4822	0.4934	45	-3.0043	**0.0043**
WT - HD	Only ATB	0.3485	0.4786	42	0.7281	0.4706
WT - HD	ATB_FMT	-0.9988	0.4918	45	-2.0310	**0.0482**

Table S18: Post-hoc results for genotype given Treatment in FITC in male

contrast	Treatment	estimate	SE	df	t.ratio	p.value
WT - HD	No treatment	-0.5967	0.2329	12	-2.5616	**0.0249**
WT - HD	Only ATB	-0.2904	0.2277	12	-1.2756	0.2262
WT - HD	ATB_FMT	-0.8545	0.2211	12	-3.8653	**0.0022**

Table S19: Post-hoc results for Treatment given genotype in Cecum Weight in male

contrast	genotype	estimate	SE	df	t.ratio	p.value
No treatment - Only ATB	WT	0.0300	0.0350	16	0.8560	0.6747
No treatment - ATB_FMT	WT	0.0850	0.0379	16	2.2455	0.0936
Only ATB - ATB_FMT	WT	0.0550	0.0379	16	1.4530	0.3388
No treatment - Only ATB	HD	-0.0193	0.0363	16	-0.5317	0.8571
No treatment - ATB_FMT	HD	0.0477	0.0410	16	1.1626	0.4914
Only ATB - ATB_FMT	HD	0.0670	0.0400	16	1.6768	0.2442

For Review Only

Table S20: Post-hoc results for genotype given Treatment in Colon length in male

contrast	Treatment	estimate	SE	df	t.ratio	p.value
WT - HD	No treatment	1.2244	0.4248	23	2.8821	**0.0084**
WT - HD	Only ATB	0.4483	0.3918	22	1.1443	0.2648
WT - HD	ATB_FMT	0.8804	0.3882	23	2.2675	**0.0331**

Table S21: Post-hoc results for genotype Treatment in Acetate in male

contrast	estimate	SE	df	t.ratio	p.value
WT No treatment - HD No treatment	9.5017	18.6410	12	0.5097	0.9948
WT No treatment - WT Only ATB	10.3542	16.6730	12	0.6210	0.9872
WT No treatment - HD Only ATB	-37.8161	17.4868	12	-2.1625	0.3206
WT No treatment - WT ATB_FMT	20.1300	16.6730	12	1.2073	0.8254
WT No treatment - HD ATB_FMT	-10.0521	17.4868	12	-0.5748	0.9909
HD No treatment - WT Only ATB	0.8525	18.6410	12	0.0457	1.0000
HD No treatment - HD Only ATB	-47.3178	19.3723	12	-2.4425	0.2161
HD No treatment - WT ATB_FMT	10.6283	18.6410	12	0.5702	0.9913
HD No treatment - HD ATB_FMT	-19.5538	19.3723	12	-1.0094	0.9061
WT Only ATB - HD Only ATB	-48.1703	17.4868	12	-2.7547	0.1342
WT Only ATB - WT ATB_FMT	9.7758	16.6730	12	0.5863	0.9901
WT Only ATB - HD ATB_FMT	-20.4063	17.4868	12	-1.1670	0.8439
HD Only ATB - WT ATB_FMT	57.9461	17.4868	12	3.3137	0.0539
HD Only ATB - HD ATB_FMT	27.7640	18.2644	12	1.5201	0.6593
WT ATB_FMT - HD ATB_FMT	-30.1821	17.4868	12	-1.7260	0.5414

For Review Only

Table S22: Post-hoc results for Treatment given genotype in IL17E in male

contrast	genotype	estimate	SE	df	t.ratio	p.value
No treatment - Only ATB	WT	0.0321	0.1801	14	0.1782	0.9827
No treatment - ATB_FMT	WT	-0.4044	0.1819	14	-2.2229	0.1018
Only ATB - ATB_FMT	WT	-0.4365	0.1877	14	-2.3252	0.0850
No treatment - Only ATB	HD	0.2024	0.2292	14	0.8833	0.6592
No treatment - ATB_FMT	HD	0.1352	0.2339	14	0.5783	0.8337
Only ATB - ATB_FMT	HD	-0.0672	0.1952	14	-0.3442	0.9371

Table S23: Linear mixed model results for Propionate, Isobutyrate and Methylbutyrate (Week 14) for females

	Dependent variable:		
	Propionate-14	Isobutyrate-14	Methylbutyrate-14
	(1)	(2)	(3)
genotypeHD	−0.7309	−0.7858	0.4986
	p = 0.9007	p = 0.3314	p = 0.1249
TreatmentOnly ATB	0.1275	−0.0158	0.0564
	p = 0.9821	p = 0.9834	p = 0.8497
TreatmentATB_FMT	−2.0237	−0.3104	−0.0682
	p = 0.7257	p = 0.6963	p = 0.8252
genotypeHD:TreatmentOnly ATB	0.2815	0.4770	−0.5904
	p = 0.9730	p = 0.6717	p = 0.1924
genotypeHD:TreatmentATB_FMT	−2.1452	1.5722	−0.4113
	p = 0.7942	p = 0.1866	p = 0.3633
Constant	9.6166*	2.9692***	0.9686***
	p = 0.0327	p = 0.0001	p = 0.0004
Observations	28	32	32
Log Likelihood	−76.5134	−48.3574	−21.2659
Akaike Inf. Crit.	169.0268	112.7149	58.5317
Bayesian Inf. Crit.	177.7551	122.7796	68.5965
Note:	+ p<0.1; * p<0.05; ** p<0.01; *** p<0.001		

Table S24: Linear mixed model results for Isobutyrate and Methylbutyrate (Week 20) for females

	Dependent variable:	
	Isobutyrate-20	Methylbutyrate-20
	(1)	(2)
genotypeHD	0.3708	0.1820
	p = 0.6891	p = 0.5637
TreatmentOnly ATB	0.4860	0.4082
	p = 0.6316	p = 0.2414
TreatmentATB_FMT	1.0963	0.4162
	p = 0.2528	p = 0.2038
genotypeHD:TreatmentOnly ATB	0.3220	−0.0468
	p = 0.8139	p = 0.9187
genotypeHD:TreatmentATB_FMT	0.0403	−0.1764
	p = 0.9754	p = 0.6909
Constant	2.3977***	0.7544**
	p = 0.0009	p = 0.0016
Observations	29	29
Log Likelihood	−44.3597	−16.9530
Akaike Inf. Crit.	104.7194	49.9061
Bayesian Inf. Crit.	113.8034	58.9900
Note:	+ p<0.1; * p<0.05; ** p<0.01; *** p<0.001	

For Review Only

Table S25: Linear mixed model results for Propionate, Isobutyrate and Methylbutyrate (Week 14) for males

	Dependent variable:		
	Propionate-14	Isobutyrate-14	Methylbutyrate-14
	(1)	(2)	(3)
genotypeHD	0.9088	0.5904	0.1806
	p = 0.5602	p = 0.2188	p = 0.2247
TreatmentOnly ATB	1.7019	0.3049	−0.1399
	p = 0.3241	p = 0.5616	p = 0.3874
TreatmentATB_FMT	−1.5247	−0.3897	−0.2389
	p = 0.4770	p = 0.5441	p = 0.2377
Constant	2.9463*	2.5912***	0.7841***
	p = 0.0469	p = 0.00002	p = 0.00002
Observations	36	35	36
Log Likelihood	−97.9223	−55.1535	−20.7752
Akaike Inf. Crit.	207.8446	122.3070	53.5503
Bayesian Inf. Crit.	216.6390	130.9109	62.3447
Note:	+ p<0.1; * p<0.05; ** p<0.01; *** p<0.001		

For Review Only

Table S26: Linear mixed model results for Isobutyrate and Methylbutyrate (Week 20) for males

	Dependent variable:	
	Isobutyrate-20	Methylbutyrate-20
	(1)	(2)
genotypeHD	0.0254	0.2137
	p = 0.9763	p = 0.7970
TreatmentOnly ATB	0.7819	1.4797+
	p = 0.3360	p = 0.0644
TreatmentATB_FMT	1.1532	−0.1066
	p = 0.1651	p = 0.8859
genotypeHD:TreatmentOnly ATB	1.4744	−0.9962
	p = 0.2282	p = 0.3887
genotypeHD:TreatmentATB_FMT	0.6947	0.3270
	p = 0.5607	p = 0.7741
Constant	2.6521***	0.6934
	p = 0.0003	p = 0.1986
Observations	32	32
Log Likelihood	−44.8358	−47.8591
Akaike Inf. Crit.	105.6716	111.7182
Bayesian Inf. Crit.	115.7364	121.7829
Note:	+ p<0.1; * p<0.05; ** p<0.01; *** p<0.001	

Table S27: Linear mixed model results for IL21, IL17A and IL17E (Week 14) for females

	Dependent variable:		
	IL21-14	IL17A-14	IL17E-14
	(1)	(2)	(3)
genotypeHD	−0.1450+	−0.0103	0.2609
	p = 0.0850	p = 0.8914	p = 0.3931
TreatmentOnly ATB	−0.0533	0.0665	0.3395
	p = 0.5576	p = 0.3849	p = 0.2725
TreatmentATB__FMT	−0.0493	0.0182	0.5259
	p = 0.6213	p = 0.8094	p = 0.1023
genotypeHD:TreatmentOnly ATB		−0.0337	0.0339
		p = 0.7521	p = 0.9413
genotypeHD:TreatmentATB__FMT		−0.0763	−0.7119
		p = 0.4781	p = 0.1278
Constant	0.5979***	0.1702**	0.6938**
	p = 0.000003	p = 0.0044	p = 0.0061
Observations	26	36	30
Log Likelihood	1.0641	14.1085	−19.8117
Akaike Inf. Crit.	9.8717	−12.2171	55.6234
Bayesian Inf. Crit.	16.4180	−1.0075	65.0479
Note:	+ p<0.1; * p<0.05; ** p<0.01; *** p<0.001		

Table S28: Linear mixed model results for IL1b, IL22 and TNFa (Week 14) for females

	Dependent variable:		
	IL1b-14	IL22-14	TNFa-14
	(1)	(2)	(3)
genotypeHD	0.8150	0.4568	0.0258
	p = 0.1036	p = 0.3588	p = 0.9825
TreatmentOnly ATB	−0.0696	−0.0976	−0.0080
	p = 0.8830	p = 0.8419	p = 0.9944
TreatmentATB__FMT	0.0325	0.7720	1.6155
	p = 0.9451	p = 0.1327	p = 0.1686
genotypeHD:TreatmentOnly ATB	−0.6118	−0.2245	0.3394
	p = 0.3681	p = 0.7459	p = 0.8378
genotypeHD:TreatmentATB__FMT	−0.1118	−1.1779	−1.5066
	p = 0.8672	p = 0.1074	p = 0.3624
Constant	0.6141+	0.6753+	0.8728
	p = 0.0768	p = 0.0614	p = 0.2794
Observations	36	36	34
Log Likelihood	−39.1337	−42.3066	−60.4890
Akaike Inf. Crit.	94.2674	100.6132	136.9779
Bayesian Inf. Crit.	105.4769	111.8228	147.6356
Note:	+ p<0.1; * p<0.05; ** p<0.01; *** p<0.001		

For Review Only

Table S29: Linear mixed model results for IL17A, IL1b and IL21 (Week 20) for females

	Dependent variable:		
	IL17A-20	IL1b-20	IL21-20
	(1)	(2)	(3)
genotypeHD	−0.1527+	0.4596	−0.0047
	p = 0.0744	p = 0.1730	p = 0.9510
TreatmentOnly ATB	−0.0509	0.2706	0.1423
	p = 0.5686	p = 0.4590	p = 0.1190
TreatmentATB_FMT	−0.1382	0.2454	−0.0622
	p = 0.1389	p = 0.5009	p = 0.4752
genotypeHD:TreatmentOnly ATB	0.0785	−0.3485	−0.2108+
	p = 0.5134	p = 0.4767	p = 0.0888
genotypeHD:TreatmentATB_FMT	0.2035+	−0.1846	0.2091+
	p = 0.0990	p = 0.6958	p = 0.0833
Constant	0.2588***	0.4852*	0.3807***
	p = 0.0002	p = 0.0440	p = 0.000002
Observations	34	34	34
Log Likelihood	20.6591	−27.8835	12.2008
Akaike Inf. Crit.	−25.3181	71.7670	−8.4017
Bayesian Inf. Crit.	−14.6605	82.4246	2.2559
Note:	+ p<0.1; * p<0.05; ** p<0.01; *** p<0.001		

Table S30: Linear mixed model results for IL22, IL6 and TNFa (Week 20) for females

	Dependent variable:		
	IL22-20	IL6-20	TNFa-20
	(1)	(2)	(3)
genotypeHD	0.4040	−0.4964	−0.4820
	p = 0.6271	p = 0.1477	p = 0.1298
TreatmentOnly ATB	0.1274	0.1559	−0.3684
	p = 0.8905	p = 0.6703	p = 0.2855
TreatmentATB_FMT	−0.0860	−0.2278	−0.3580
	p = 0.9259	p = 0.5358	p = 0.2988
genotypeHD:TreatmentOnly ATB	−0.6839	−0.0815	0.4548
	p = 0.5841	p = 0.8679	p = 0.3216
genotypeHD:TreatmentATB_FMT	0.6249	0.6418	0.7472
	p = 0.6065	p = 0.1947	p = 0.1072
Constant	0.8332	1.1527***	1.1667***
	p = 0.1632	p = 0.0001	p = 0.00005
Observations	34	34	33
Log Likelihood	−52.5298	−21.1180	−11.3178
Akaike Inf. Crit.	121.0595	58.2361	38.6356
Bayesian Inf. Crit.	131.7172	68.8937	49.0023
Note:	+ p<0.1; * p<0.05; ** p<0.01; *** p<0.001		

For Review Only

Table S31: Linear mixed model results for IL21, IL17A and IL17E (Week 14) for males

	Dependent variable:		
	IL21-14	IL17A-14	IL17E-14
	(1)	(2)	(3)
genotypeHD	−0.0558	0.0589	0.4301
	p = 0.4973	p = 0.6692	p = 0.1134
TreatmentOnly ATB	−0.0518	−0.1186	0.1212
	p = 0.5905	p = 0.4588	p = 0.6958
TreatmentATB_FMT	0.1637	−0.0928	0.3462
	p = 0.1212	p = 0.5949	p = 0.2649
Constant	0.3083***	0.2550+	0.5904*
	p = 0.0009	p = 0.0615	p = 0.0206
Observations	31	31	26
Log Likelihood	3.9388	−12.6930	−21.9144
Akaike Inf. Crit.	4.1224	37.3860	55.8288
Bayesian Inf. Crit.	11.8975	45.1610	62.3750
Note:	+ p<0.1; * p<0.05; ** p<0.01; *** p<0.001		

Table S32: Linear mixed model results for IL1b, IL22 and TNFa (Week 14) for males

	Dependent variable:		
	IL1b-14	IL22-14	TNFa-14
	(1)	(2)	(3)
genotypeHD	−0.0718	−0.0097	0.3622
	p = 0.7599	p = 0.9651	p = 0.9964
TreatmentOnly ATB	0.2181	−0.0400	−0.0461
	p = 0.3593	p = 0.8577	p = 0.9996
TreatmentATB_FMT	0.1976	0.1203	0.3905
	p = 0.4268	p = 0.6081	p = 0.9964
genotypeHD:TreatmentOnly ATB	−0.1764	−0.1169	0.2442
	p = 0.5900	p = 0.7061	p = 0.9983
genotypeHD:TreatmentATB_FMT	0.0438	−0.0483	150.3674
	p = 0.8999	p = 0.8875	p = 0.2201
Constant	0.3428+	0.5447**	0.8442
	p = 0.0529	p = 0.0032	p = 0.9876
Observations	35	34	33
Log Likelihood	−19.8067	−17.7358	−174.8050
Akaike Inf. Crit.	55.6134	51.4717	365.6099
Bayesian Inf. Crit.	66.5518	62.1293	375.9766
Note:	+ p<0.1; * p<0.05; ** p<0.01; *** p<0.001		

For Review Only

Table S33: Linear mixed model results for IL17A, IL1b and IL21 (Week 20) for males

	Dependent variable:		
	IL17A-20	IL1b-20	IL21-20
	(1)	(2)	(3)
genotypeHD	−0.0107	0.1719	−0.2490
	p = 0.8701	p = 0.7236	p = 0.1524
TreatmentOnly ATB	−0.0146	0.3688	−0.1318
	p = 0.8388	p = 0.4511	p = 0.3863
TreatmentATB_FMT	0.0685	−0.1591	0.0579
	p = 0.3454	p = 0.7535	p = 0.7138
genotypeHD:TreatmentOnly ATB	0.0787	0.3263	0.1306
	p = 0.4319	p = 0.6426	p = 0.5842
genotypeHD:TreatmentATB_FMT	0.0824	0.1651	0.1002
	p = 0.4164	p = 0.8172	p = 0.6710
Constant	0.1448**	0.6860+	0.7783***
	p = 0.0062	p = 0.0581	p = 0.000003
Observations	35	39	30
Log Likelihood	17.3611	−47.8369	−6.1180
Akaike Inf. Crit.	−18.7222	111.6737	28.2359
Bayesian Inf. Crit.	−7.7838	123.6458	37.6604
Note:	+ p<0.1; * p<0.05; ** p<0.01; *** p<0.001		

Table S34: Linear mixed model results for IL22, IL6 and TNFa (Week 20) for males

	Dependent variable:		
	IL22-20	IL6-20	TNFa-20
	(1)	(2)	(3)
genotypeHD	−0.0978	−0.2050	−0.9973
	p = 0.7838	p = 0.7827	p = 0.5521
TreatmentOnly ATB	0.1930	0.0898	−0.7883
	p = 0.5895	p = 0.9038	p = 0.6377
TreatmentATB_FMT	−0.1003	1.0858	−0.8374
	p = 0.7868	p = 0.1762	p = 0.6308
genotypeHD:TreatmentOnly ATB	0.5237	0.3921	0.6315
	p = 0.3159	p = 0.7163	p = 0.7933
genotypeHD:TreatmentATB_FMT	0.3911	−0.3485	4.3945+
	p = 0.4593	p = 0.7500	p = 0.0956
Constant	1.1129***	0.8100	1.7749
	p = 0.0004	p = 0.1356	p = 0.1458
Observations	39	39	38
Log Likelihood	−38.4919	−59.1479	−86.8274
Akaike Inf. Crit.	92.9839	134.2957	189.6548
Bayesian Inf. Crit.	104.9559	146.2678	201.3807
Note:	+ p<0.1; * p<0.05; ** p<0.01; *** p<0.001		

For Review Only
