# Supplementary figures and images for: Faecal microbiota transplant ameliorates gut dysbiosis and cognitive deficits in Huntington’s disease mice
Source: Brain Commun. 2022 Aug 12;4(4):fcac205. doi: 10.1093/braincomms/fcac205 (PMC9400176; doi:10.1093/braincomms/fcac205)

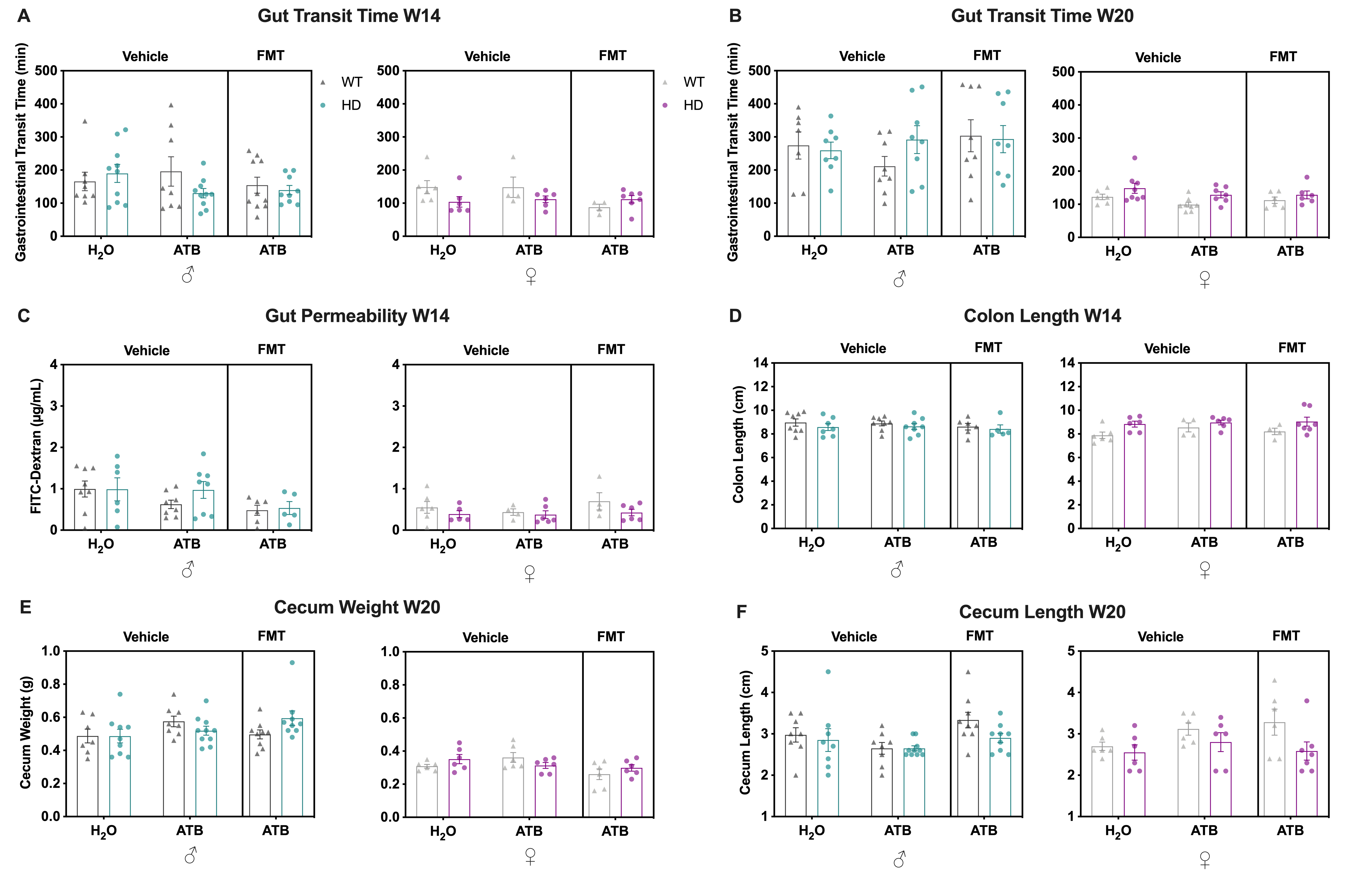

Supplement: fcac205_Supplementary_Data [file fcac205_supplementary_data.zip › Supplementary Figure 1.png]

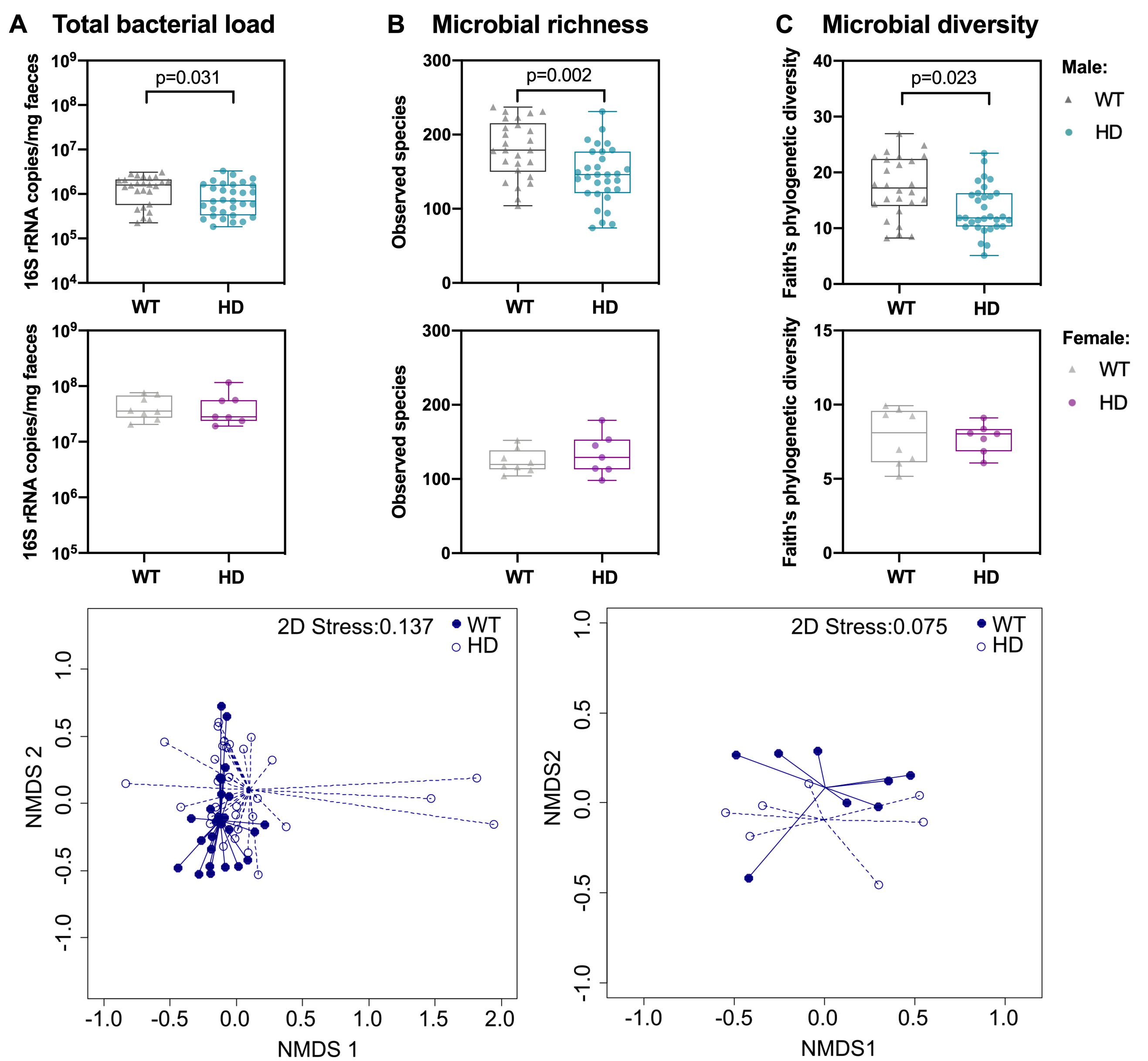

Supplement: fcac205_Supplementary_Data [file fcac205_supplementary_data.zip › Supplementary Figure 2.png]

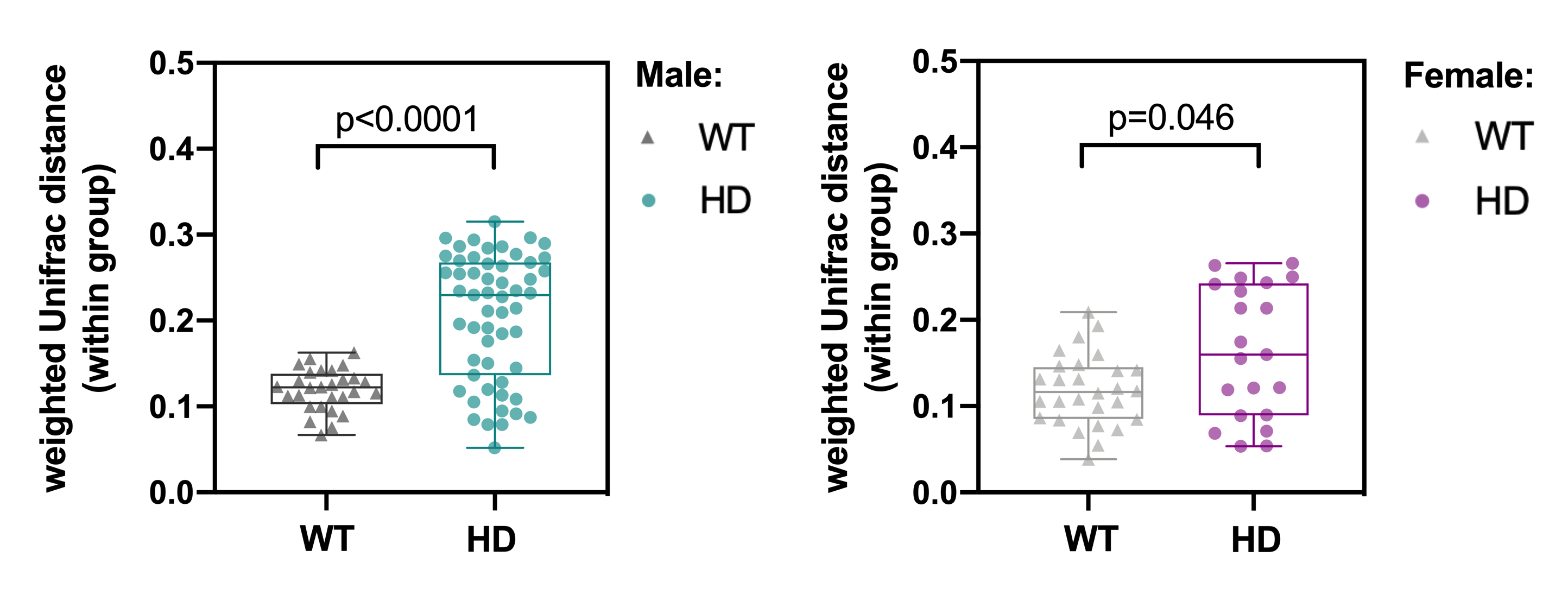

Supplement: fcac205_Supplementary_Data [file fcac205_supplementary_data.zip › Supplementary Figure 3.png]
