# Supplementary material for: Faecal microbiota transplant ameliorates gut dysbiosis and cognitive deficits in Huntington’s disease mice
Source: Brain Commun. 2022 Aug 12;4(4):fcac205. doi: 10.1093/braincomms/fcac205 (PMC9400176; doi:10.1093/braincomms/fcac205)
Supplement: fcac205_Supplementary_Data [file Original-submission-decision-letter.pdf › Original-submission-decision-letter.pdf]

Subject: Brain Communications - Decision on Manuscript ID BRAINCOM-2022-109

Body: 16-May-2022

Dear Tony,

Thank you for your recent submission BRAINCOM-2022-109 entitled "Fecal microbiota transplant ameliorates gut dysbiosis and cognitive deficits in Huntington's disease mice" to Brain Communications.

Your manuscript has been reviewed, and while the reviewer had largely positive comments, they have some constructive critiques that I think you could address in a minor revision of the manuscript. Therefore, I invite you to respond to the reviewer's comments and revise your manuscript. The reviewer's comments are included at the bottom of this message.

In addition to the reviewers' comments, please also address our Scientific Editor's comments added below to comply with our requirements for presentation of scientific data at Brain Communications.

Please format your revised manuscript according to our Instructions to Authors https://academic.oup.com/braincomms/pages/General_Instructions – a summary of key points is attached to help with this.

To revise your manuscript, click the link below or log into https://mc.manuscriptcentral.com/braincom and enter your Author Centre, where you will find your manuscript title listed under "Manuscripts with Decisions." Under "Actions," click on "Create a Revision." Your manuscript number will be appended to denote a revision.

*** PLEASE NOTE: This is a two-step process. After clicking on the link, you will be directed to a webpage to confirm. ***

https://mc.manuscriptcentral.com/braincom?URL_MASK=18d8875073344eba9a0626a58428f44c

Please upload a revised manuscript with the changes you have made shown either in coloured text or using "track changes".

When submitting your revised manuscript, you will be asked to respond to the reviewer(s) comments. Please be as specific as possible in your response to all of the comments.

The editorial team at Brain Communications also aims to increase transparency in scientific publishing and to demonstrate the value that peer review brings to the scientific process. Please therefore consider whether you are willing to have reviewer comments on your paper and your response published as supplementary data with the paper, if it is accepted. This is entirely optional and will only be done if both the authors and reviewers agree. When submitting your revision you will have the opportunity to update the answer you entered previously.

IMPORTANT: Your original files are available to you when you upload your revised manuscript. Please delete any redundant files before completing the submission.

Because we are trying to facilitate timely publication of manuscripts submitted to Brain Communications, we would appreciate your revised manuscript as soon as possible. If it is not possible for you to resubmit within 3 months, the option to submit a revision will expire, and we would then consider your revised paper as a new submission. However, if you need a little more time, please get in touch and we can extend this deadline for you.

Once again, thank you for submitting your manuscript to Brain Communications and I look forward to receiving your revision.

Best wishes

Professor Tara Spires-Jones
Founding Editor, Brain Communications

Reviewer(s)' Comments to Author:

Reviewer: 1

Comments to the Author
The huntingtin gene is ubiquitously expressed and several disease-related phenotypes affecting peripheral tissues and organs have been described. This includes the gastrointestinal system and evidence for gut microbiome disruption has been reported. The microbiota-gutbrain axis provides a potential pathway via which changes in the gut could modulate brain function. In this paper, the authors explore the potential of the gut microbiome as a therapeutic target. They have used fecal microbiota transplant (FMT) from wildtype (WT) to R6/1 HD mice and investigated whether this might modulate motor and cognitive behaviors, showing some improvement in the cognition of female HD mice. They demonstrated that gut dysfunction is a characteristic of the HD phenotype and that this was not modulated by treatment. They found that the FMT engraftment of WT microbiotica was less efficient in male HD mice which may be due to the more pronounced changes of the structure, composition and instability of the gut microbial community in male HD mice.

Overall, this is a comprehensive and well-designed study that presents a great deal of data. The results are complex and we are presented with a lot of p-values that are not that easy to interpret. But the authors are correct in stating that this study will inform future microbiotica-targeted therapies for HD.

There are some minor issues to be addressed:

Huntingtin does not need to be italicised

Materials and Methods

Page 4, line 37: the inbred strain sub-strains and suppliers are not given for the mice.

Page 6, line 6: ATB is not defined

Page 7, line 8: when being tested on a rotarod, mice sometime cling on to the rotating rod and turn with it rather than walking. Did this occur, and if so, how was it scored.

Page 7, line 31: did the mouse have to clasp for the full 30 sec, or just at some point during this time?

Page 9, line 10: CS and US are not defined

Page 11, line 13: the title of this section refers to both SCFA and BCFA, but only SCFAs are mentioned in the protocol.

Page 14, line 25: data are processed…..

Page 14, line 50: CFC is not defined

Results

Page 15, line 44: the following sentence does not make sense:
‘Overall, in males, ATB decreased body weight ($p<0.0001$), but increased body weight ($p=0.0002$)

and decreased food intake in HD mice ($p<0.0001$).'

Fig. 1A: GTT is not defined

Fig. 1G: the labelling on this figure is illegible and it is not clear what treatment group these panels represent. The labelling on Supplementary Fig. 1 is also illegible.

Comments from the Scientific Editor:

-Fig1-e , Fig2d,e , Fig3a,b: please, clarify in the legend if each line represents a single data point.

-Fig1c-e: please, report in the legend the number of mice/cage.

-Fig1f,h , Fig2b,d,e , Fig3, Fig4 , Fig5b, Fig6, Fig7 , Supp Fig2, Supp3a-c and Supp Fig4: please, clarify in the legend if each data point represents either a mouse or a cage.

-Please supply a graphical abstract. A graphical abstract is a visual representation of the central finding or methodology of the paper. It will be displayed in the table of contents. Authors should strive to make the graphical abstract informative, interesting, visually appealing, and straightforward (no caption needed, no abbreviations except gene names). Please upload as a separate high resolution image file (tiff preferred). The technical requirements for the graphical abstracts are:
--size of the submitted image: 1200 pixels square at 300dpi
--font of the text: Arial 12-16 point (smaller fonts will not be legible online)

-Please add an Abbreviated Summary (up to 50 words) of your paper that captures the main purpose and conclusions of the work. The summary should state the main results written in the third person ('[1st author surname] et al. report that …') and conclusion. This summary will be used in the contents list online and should not contain any abbreviations other than those on the accepted abbreviations list and accepted gene/protein names, making it accessible to the non-specialist. Please upload it as a Word document. Allowed abbreviations: AIDS; ANOVA; AMPA; ATP; A,T,C,G; CNS; CSF; CT; DNA; ECG; EEG; EMG; GABA; HIV; MRI; NMDA; PET; PCR; RNA.

-Please include the page numbers corresponding to the items listed in the checklist.
