# Supplementary material for: Faecal microbiota transplant ameliorates gut dysbiosis and cognitive deficits in Huntington’s disease mice
Source: Brain Commun. 2022 Aug 12;4(4):fcac205. doi: 10.1093/braincomms/fcac205 (PMC9400176; doi:10.1093/braincomms/fcac205)
Supplement: fcac205_Supplementary_Data [file Response_to_reviewers_comments_original.pdf › Response_to_reviewers_comments_original.pdf]

We greatly appreciate this constructive review of our manuscript. We have comprehensively addressed each and every Reviewer and Scientific Editor comment, as detailed below. In the extensively revised manuscript, which we now attach, we have highlighted all of our changes.

Reviewer: 1

There are some minor issues to be addressed:

Huntingtin does not need to be italicised

RESPONSE: Done

Materials and Methods

Page 4, line 37: the inbred strain sub-strains and suppliers are not given for the mice.

RESPONSE: This information was added as follows:
Male R6/1 hemizygous mice (originally from Jackson Laboratories, USA) after back-crossing onto the CBA background for more than 10 generations (generating the CBAxC57Bl/6 strain background) were crossed with female CBAxC57Bl/6 mice to generate male and female wild-type (WT) and R6/1 (HD) littermates.

Page 6, line 6: ATB is not defined

RESPONSE: Done

Page 7, line 8: when being tested on a rotarod, mice sometime cling on to the rotating rod and turn with it rather than walking. Did this occur, and if so, how was it scored.

RESPONSE: This explanation was added as follows:
Mice that clung to the rotating rod or changed orientation during the test, not turning back and/or getting back to the upright walk for 3 consecutive turns, were removed from the apparatus, and the maximum score was assigned.

Page 7, line 31: did the mouse have to clasp for the full 30 sec, or just at some point during this time?

RESPONSE: This information was clarified as follows:
Mice are suspended by their tails for 30 seconds per session, while observing the clasping action of the paws during this time.

Page 9, line 10: CS and US are not defined

RESPONSE: Done

Page 11, line 13: the title of this section refers to both SCFA and BCFA, but only SCFAs are mentioned in the protocol.

RESPONSE: Done

[8]Page 14, line 25: data are processed…..

Page 14, line 50: CFC is not defined

RESPONSE: Done

Results

Page 15, line 44: the following sentence does not make sense:
'Overall, in males, ATB decreased body weight ($p<0.0001$), but increased body weight ($p=0.0002$) and decreased food intake in HD mice ($p<0.0001$).'

RESPONSE: We restructured the sentence as follows:
In males, ATB decreased body weight ($p<0.0001$). In HD mice, ATB increased body weight ($p=0.0002$) and decreased food intake ($p<0.0001$).

Fig. 1A: GTT is not defined

RESPONSE: Done

Fig. 1G: the labelling on this figure is illegible and it is not clear what treatment group these panels represent. The labelling on Supplementary Fig. 1 is also illegible.

RESPONSE: We have re-plotted the figures clarifying the groups and increasing the label fonts. Please note that Supp Fig 1 does not exist anymore since was incorporated it to the updated Fig1G.

Comments from the Scientific Editor:

Fig1-e , Fig2d,e , Fig3a,b: please, clarify in the legend if each line represents a single data point.

RESPONSE: Done

Fig1c-e: please, report in the legend the number of mice/cage.

RESPONSE: Done

Fig1f,h , Fig2b,d,e , Fig3, Fig4 , Fig5b, Fig6, Fig7 , Supp Fig2, Supp3a-c and Supp Fig4: please, clarify in the legend if each data point represents either a mouse or a cage.

RESPONSE: Done

Please supply a graphical abstract. A graphical abstract is a visual representation of the central finding or methodology of the paper. It will be displayed in the table of contents. Authors should strive to make the graphical abstract informative, interesting, visually appealing, and straightforward (no caption needed, no abbreviations except gene names). Please upload as a separate high resolution image file (tiff preferred). The technical requirements for the graphical abstracts are:
  --size of the submitted image: 1200 pixels square at 300dpi
  --font of the text: Arial 12-16 point (smaller fonts will not be legible online)

RESPONSE: It has been uploaded.

Please add an Abbreviated Summary (up to 50 words) of your paper that captures the main purpose and conclusions of the work. The summary should state the main results written in the third person ('[1st author surname] et al. report that …') and conclusion. This summary will be used in the contents list online and should not contain any abbreviations other than those on the accepted abbreviations list and accepted gene/protein names, making it accessible to the non-specialist. Please upload it as a Word document. Allowed abbreviations: AIDS; ANOVA; AMPA; ATP; A,T,C,G; CNS; CSF; CT; DNA; ECG; EEG; EMG; GABA; HIV; MRI; NMDA; PET; PCR; RNA.

RESPONSE: It has been uploaded,

Please include the page numbers corresponding to the items listed in the checklist.

RESPONSE: We have addressed all items in the checklist as follows.

Main text file – attached.

Abstract – page 1 of main manuscript doc.

Keywords – page 2 of main manuscript doc.

Materials and methods – starting page 4 of main manuscript and includes statistical analysis paragraph and data availability paragraph.

Funding section - page 25 of main manuscript and includes names of funding bodies in full

Competing interests - page 25 of main manuscript

References – starts page 26 of main manuscript

Supplementary Tables - uploaded as separate file.

Figure legends – start page 30 of main manuscript

Figures – uploaded as separate high-resolution image files.

Supplementary figures – uploaded as separate files.

Graphical abstract - uploaded as a separate image file.

Abbreviated summary – uploaded as a separate Word file.
