# Supplementary material for: Faecal microbiota transplant ameliorates gut dysbiosis and cognitive deficits in Huntington’s disease mice
Source: Brain Commun. 2022 Aug 12;4(4):fcac205. doi: 10.1093/braincomms/fcac205 (PMC9400176; doi:10.1093/braincomms/fcac205)
Supplement: fcac205_Supplementary_Data [file Response_to_reviewers_comments_revision_1.pdf › Response_to_reviewers_comments_revision_1.pdf]

Reviewer(s)' Comments to Author:

-Please include the page numbers corresponding to the items listed in the checklist.

We have now added the page numbers as requested.
